# Targeted degradation of hepatic KEAP1 mitigates drug-induced liver injury *via* dual boosting NRF2 and PGAM5 signaling

**DOI:** 10.1016/j.redox.2026.104296

**Published:** 2026-07-11

**Authors:** Yanyan Deng, Leizhi Xu, Xiaoting Niu, Jingjing Li, Yuan Xiong, Guanghao Zhu, Zhiyi Lu, Chuting Xu, Xuerui Wang, Pu Wang, Jian Huang, Zhangping Xiao, Frank J. Gonzalez, Lili Ji, Caixia Sun, Ping Wang, Guangbo Ge

**Affiliations:** a State Key Laboratory of Discovery and Utilization of Functional Components in Traditional Chinese Medicine, Shanghai Frontiers Science Center of TCM Chemical Biology, Institute of Interdisciplinary Integrative Medicine Research, Shanghai University of Traditional Chinese Medicine, Shanghai, 201203, China; b Pharmacology and Toxicology Division, Shanghai Institute of Food and Drug Control, Shanghai, 201203, China; c The MOE Key Laboratory for Standardization of Chinese Medicines, Shanghai Key Laboratory of Compound Chinese Medicines and The SATCM Key Laboratory for New Resources and Quality Evaluation of Chinese Medicines, Institute of Chinese Materia Medica, Shanghai University of Traditional Chinese Medicine, Shanghai, 201203, China; d Cancer Innovation Laboratory, Center for Cancer Research, National Cancer Institute, National Institutes of Health, Bethesda, MD, 20892, USA

**Keywords:** KEAP1 degraders, PROTACs, Drug-induced liver injury (DILI), KEAP1-NRF2 axis, Mitochondrial quality control (MQC)

## Abstract

Oxidative stress and impaired mitochondrial homeostasis are critical drivers of drug-induced liver injury (DILI), both of which are negatively regulated by Kelch-like ECH-associated protein 1 (KEAP1). In this study, a dual-action strategy was adapted to develop a novel KEAP1 degrader with high liver exposure for mitigating DILI through concurrent activation of the NRF2 and PGAM5 signaling pathways. Following screening of a natural product library using complementary KEAP1 thermal shift and NRF2 luciferase reporter assays, cardamonin (**CAD**) was identified as a natural KEAP1 binder. A series of **CAD**-derived proteolysis-targeting chimeras (PROTACs) was subsequently designed and synthesized, leading to the discovery of compound **8L** that effectively degraded KEAP1 in hepatocytes. *In vivo*, **8L** exhibited marked preferential distribution in the liver, a favorable safety profile, and significant hepatoprotective effects in both acetaminophen- and cisplatin-induced liver injury mouse models. Notably, targeted degradation of hepatic KEAP1 by **8L** concurrently activated the NRF2-mediated antioxidative program and the PGAM5-regulated mitochondrial integrity program, which cooperatively restored mitochondrial homeostasis and counteracted oxidative stress. Collectively, a liver-preferential KEAP1 degrader was developed to mitigate DILI by dual activation of the NRF2 and PGAM5 pathways, offering a promising therapeutic agent and more in-depth mechanistic insights into KEAP1-targeted degradation for enhanced anti-DILI therapy.

## Graphical abstract

A novel KEAP1 degrader, **8L**, with high hepatic exposure, was devoloped for activating both NRF2 and PGAM5 *via* a dual-action strategy, offering an innovative and promising therapeutic strategy for DILI.

**Figure:**
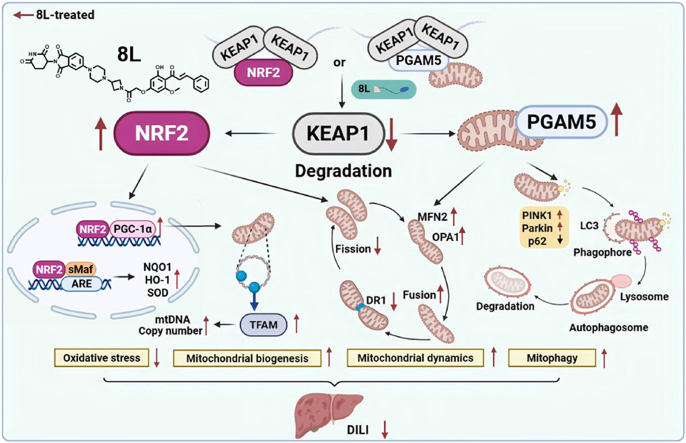

## 1 Introduction

As the primary organ responsible for xenobiotic metabolism, the liver is prone to generating excessive reactive oxygen species (ROS) during drug biotransformation [1,2], making it particularly susceptible to damage from therapeutic agents [3], phytochemicals [4], and toxins [5]. Sustained oxidative stress impairs mitochondrial integrity and function, ultimately triggering hepatocyte apoptosis and mediating drug-induced liver injury (DILI) [6,7]. As the most frequent and clinically consequential form of hepatotoxicity, DILI results from exposure to medications, herbal remedies, or other xenobiotics [8]. Notably, DILI has emerged as a leading cause of acute liver failure (accounting for approximately 50% of cases) and one of the main reasons for drug withdrawals from the market [9]. Over the past few decades, although significant advances have been made in elucidating the molecular mechanisms and key pathogenic events of DILI triggered by various medications, effective therapeutic options against DILI remain strikingly limited [1,10]. To the best of our knowledge, current clinical management is primarily limited to discontinuing the causative agents or administering N-acetylcysteine (NAC) [11], thus underscoring the urgent need for novel, mechanism-based treatments.

Targeting the pivotal pathogenic events in DILI (such as oxidative stress and dysregulated mitochondrial quality control) has emerged as a promising therapeutic strategy. Among all identified key targets or pathways, the KEAP1-NRF2 signaling pathway represents a critical cytoprotective mechanism against both oxidative stress and mitochondrial dysfunction [12,13], making it a compelling therapeutic target for mitigating DILI [14]. The regulatory function of this pathway primarily depends on KEAP1, which serves as a substrate adaptor within the Cul3-RBX1 E3 ubiquitin ligase complex, orchestrating the ubiquitination and degradation of various substrates [15,16]. Notably, recent studies have identified phosphoglycerate mutase family member 5 (PGAM5) as a novel substrate of the KEAP1-dependent ubiquitin ligase complex [17,18]. At the subcellular level, PGAM5 anchors the KEAP1-NRF2 complex to the mitochondrial outer membrane *via* its N-terminal mitochondrial targeting sequence, forming a stable PGAM5-KEAP1-NRF2 ternary complex [19]. Within this complex, KEAP1 plays a crucial bridging role: on one hand, it mediates the cytoplasmic retention of NRF2, preventing its nuclear translocation and subsequent activation of downstream antioxidant genes [20,21]; on the other hand, it promotes the degradation of PGAM5, thereby suppressing its function in mitochondrial quality control [[22], [23], [24]]. In DILI, disruption of the bridging function of KEAP1 within this complex releases both NRF2 and PGAM5, allowing each to exert its protective functions. On one hand, NRF2 orchestrates protective responses by upregulating a suite of antioxidative and detoxification enzymes [[25], [26], [27]]; on the other hand, PGAM5 acts at the mitochondrial level to promote homeostasis restoration, including enhancing PINK1/Parkin-mediated mitophagy. In parallel, both factors contribute to promoting mitochondrial biogenesis and maintaining mitochondrial dynamics [28].

To develop efficacious hepatoprotective agents, previous studies have primarily focused on the KEAP1-NRF2 axis. However, the potential role of the NRF2-KEAP1-PGAM5 signaling network has not been systematically explored and deserves further investigation [28,29]. The most previously reported NRF2 agonists act as covalent KEAP1 modifiers, which are limited by off-target effects, inadequate liver exposure, consequent narrow therapeutic windows and safety concerns [30]. In striking contrast, proteolysis targeting chimeras (PROTACs) technique represents a paradigm-shifting therapeutic strategy that enables enzymatic degradation of the protein of interest (POI) [31,32]. This innovative approach circumvents the limitations of traditional agonists while offering sustained efficacy and enhanced specificity through complete removal of the POI [33,34]. Furthermore, structural optimization of PROTACs may generate organ-specific agents that facilitate organ-selective degradation of the POI, thereby enabling precision therapeutic interventions while minimizing systemic side effects [[35], [36], [37]]. Therefore, KEAP1-targeted degraders represent a mechanism-based intervention strategy that concurrently relieves the suppression of NRF2 and PGAM5, synergistically enhancing antioxidant capacity and restoring mitochondrial homeostasis, thereby effectively mitigating DILI.

In this study, a dual-action strategy was adapted to devise a novel mechanistically-elucidated and liver-enriched KEAP1-PROTAC that mitigates DILI through co-activating both NRF2 and PGAM5 signaling pathways. Initially, cardamonin (**CAD**) was identified as a natural KEAP1 binder through phenotypic screening of a natural product library using complementary KEAP1 thermal shift and NRF2 luciferase reporter assays. Subsequently, a series of **CAD**-derived PROTACs were rationally designed and chemically synthesized *via* introducing various linkers coupling with pomalidomide (a widely used binder of the E3 ubiquitin ligase) at C-4 phenolic group of **CAD**. Among these candidates, compound **8L** demonstrated the most potent KEAP1-degrading activity in hepatocytes. Further investigation revealed that **8L** demonstrates high liver exposure and possesses excellent degradation specificity towards KEAP1, prompting its selection as a lead candidate for evaluating hepatoprotective efficacy. A comprehensive study of *in vitro* and *in vivo* assays confirmed the **8L** exhibits impressive performance against DILI. Mechanistic studies further elucidated **8L** mediated KEAP1 proteasomal degradation and revealed its dual regulatory role in activating the NRF2 and PGAM5 signaling axes.

## 2 Materials and methods

### 2.1 Chemicals and reagents

Cisplatin (CDDP) and acetaminophen (APAP) were obtained from Shanghai Nature Standard (Shanghai, China). Chloroquine (CQ), cycloheximide (CHX), and MG-132 were purchased from MedChemExpress (New Jersey, USA). Assay kits for lactate dehydrogenase (LDH), superoxide dismutase (SOD), malondialdehyde (MDA), GSH/GSSG, and ATP were provided by Merck (Darmstadt, Germany). Antibodies against TFAM (ab307302), PGC-1α(ab77210), MFN2 (ab124773), Parkin (ab77924), PGAM5 (ab126534), DRP1 (ab184247), KEAP1 (ab139729), LC3-II (ab192890) and P62 (ab109012) were supplied by Abcam (Massachusetts, USA). The PINK1 (#6946), NRF2 (#12721) and β-actin (#4967) antibody was sourced from Cell Signaling Technology (Massachusetts, USA). Apoptosis detection kit, mitochondrial permeability transition pore (MPTP) assay kit and MitoSOX Red Mitochondrial Superoxide Indicator (M36008) was procured from Thermo Fisher Scientific (Massachusetts, USA). Additional reagents were provided by MedChemExpress (New Jersey, USA). PAGE quick preparation kits (GF1820) were obtained from Genefist Life Science (Guangzhou, China).

### 2.2 Synthesis of **CAD** derivatives

The synthesis commenced with site-specific modification of **CAD** using tert-butyl bromoacetate, followed by deprotection with 4 M hydrochloric acid to yield key intermediate **3**. Subsequently, intermediate **3** was coupled with pomalidomide derivatives through diverse linker strategies. Specifically, fluoro-thalidomide reacts with N-Boc-protected diamines in the presence of N, N-diisopropylethylamine (DIEA) as a base in DMSO. After that, the Boc protecting group was removed under acidic conditions to generate intermediate **7**. These intermediates are ultimately coupled with key intermediate **3** through the formation of an amide bond. This multi-step synthetic scheme successfully yields a series of PROTAC molecules (**8A**-**8M**). The synthetic details are presented in Supplementary Scheme S1-3.

### 2.3 Experimental animal protocols

All animal experiments were conducted in accordance with the National Institutes of Health Guide for the Care and Use of Laboratory Animals and were approved by the Animal Care Committee of Shanghai University of Traditional Chinese Medicine (Approval No. PZSHUTCM2311130003). Male C57BL/6J mice (8–10 weeks old) were obtained from Shanghai Jihui and maintained under standard conditions (22 ± 1 °C, 40–60% humidity, 12 h light/dark cycle) with free access to food and water. For hepatoprotective assessment, the mice (n = 8 per group) were randomly divided into 12 groups:(1) Ctrl-1 (vehicle control-1), (2) CDDP (20 mg/kg), (3) CDDP + **CAD** (20 mg/kg each), (4) CDDP + **8L** low (2.5 mg/kg), (5) CDDP + **8L** medium (5 mg/kg), (6) CDDP + **8L** high (10 mg/kg), (7) Ctrl-2 (vehicle control-2), (8) APAP (500 mg/kg), (9) APAP + **CAD** (20 mg/kg each), (10) APAP + **8L** low (2.5 mg/kg), (11) APAP + **8L** medium (5 mg/kg), (12) APAP + **8L** high (10 mg/kg).

### 2.4 Safety assessment and tolerability test of **8L**

Male C57BL/6J mice (n = 10, 20-22 g) were acclimatized for one week under standard conditions (22 ± 2 °C, 55 ± 5% humidity). Following acclimatization, the mice received daily intraperitoneal injections of either **8L** (50 mg/kg) or saline for 14 days. Body weight and general health were recorded throughout the study. At the endpoint, blood was collected for biochemical analysis, and major organs (heart, liver, spleen, lungs, kidneys, intestine, and brain) were harvested for histological assessment using H&E staining.

### 2.5 Metabolic stability assays

The metabolic stability of test compounds (10 μM) was evaluated in Phase I and II reaction systems. For Phase I, human liver microsomes (HLMs, 0.1 mg/mL) were incubated in 0.1 M PBS (pH 7.4) with 4 mM MgCl_2_. Reactions were started by adding 1 mM NADPH and stopped with ice-cold acetonitrile at timed points from 0 to 45 min. Phase II metabolism was assessed in Tris-HCl buffer (50 mM, pH 7.4) containing 0.5 mg/mg protein Brij58, 5 mM MgCl_2_, and 2 mM UDPGA. All samples were analyzed by HPLC-UV (LC-20A, Kyoto, Japan).

### 2.6 Cell viability and lactate dehydrogenase (LDH) release assays

HepaRG cells were seeded in 96-well plates at 1.0 × 10^4^ cells per well and allowed to adhere overnight. After pretreatment with **8L** or **CAD** (1.25 and 2.5 μM) for 1 h, the cells were co-treated with CDDP or APAP for 24 h. Cell viability and LDH release were then measured using established methods.

### 2.7 Oxidative stress biomarker quantification

Following centrifugation of liver homogenates (10,000 × g, 15 min, 4 °C), the supernatant fractions were analyzed to determine MDA, GSH, and SOD concentrations using standardized commercial kits.

### 2.8 Total ROS and mitochondrial ROS detection

The levels of total and mitochondrial ROS in HepaRG cells were evaluated by DCFH-DA kit and MitoSOX Red Mitochondrial Superoxide Indicator, respectively. After intervention with drugs, the cells were reacted with a 5 μM MitoSOX working solution at 37 °C for 30 min in the dark, and then washed with PBS and then incubated with DAPI for 10 min. Microscopically detected fluorescence was digitized and analyzed for intensity values using ImageJ.

### 2.9 Mitochondrial function assays

Mitochondrial membrane potential (MMP) and mitochondrial permeability transition pore (MPTP) opening were assessed using JC-1 and Calcein AM fluorescence, respectively, analyzed by flow cytometry and fluorescence microscopy. Data analysis was performed with FlowJo and ImageJ software.

### 2.10 ATP measurement

ATP levels in HepaRG cells and tissues were determined using an enhanced ATP assay kit according to the manufacturer's instructions. The ATP concentration was quantified based on a standard curve generated using a Spectramax M4 microplate reader (Molecular Devices, USA). Results were normalized to the protein content of each sample, as measured by a bicinchoninic acid (BCA) protein assay kit.

### 2.11 Mitochondrial autophagosome assay

HepaRG cells were seeded in 12-well plates for detection of mitochondrial autophagosomes using a Mitophagy Detection Kit according to the manufacturer's instructions. The mitophagy dye accumulates and binds to intact mitochondria, exhibiting weak fluorescence under basal conditions. Upon induction of mitophagy, damaged mitochondria fuse with lysosomes, resulting in significantly enhanced fluorescence. Mitochondrial-lysosomal fusion events were visualized by fluorescence microscopy.

### 2.12 Mice liver-specific KEAP1 knockout experiment

The efficacy of **8L** to mitigate APAP-induced liver injury was also investigated in Hep^cre^-*KEAP1*^*flox/flox*^ mice. Fifteen Hep^cre^-*KEAP1*^*flox/flox*^ mice were divided into three groups (n = 5 per group): Hep^cre^-*KEAP1*^*flox/flox*^ control, Hep^cre^-*KEAP1*^*flox/flox*^ APAP model (500 mg/kg) and Hep^cre^-*KEAP1*^*flox/flox*^ APAP (500 mg/kg) + **8L** (10 mg/kg). APAP and **8L** treatment protocols were identical with those used in the above-mentioned animal experiments.

### 2.13 Statistical analyses

All statistical analyses were conducted using GraphPad Prism 8.0 (La Jolla, CA, USA). Data are presented as mean ± standard deviation (SD). Group comparisons were analyzed using unpaired Student’Hs t-tests, one-way ANOVA, or two-way ANOVA, as appropriate. Statistical significance was defined as *∗P < 0.05, ∗∗P < 0.01, ∗∗∗P < 0.001.*

## 3 Results

### 3.1 Design and synthesis of the degrader candidates of KEAP1

**Fig. 1:**
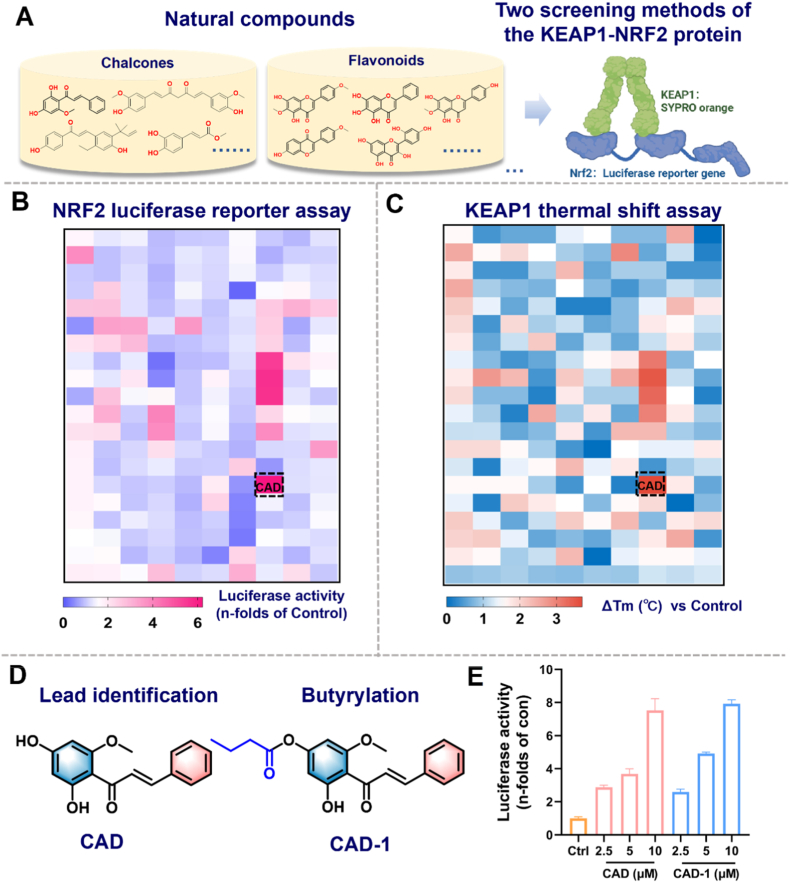
**Discovery of cardamonin (CAD) as a precursor for constructing degraders of KEAP1 and a potent NRF2 agonist. (A)** An in-house natural product library. **(B)** Screening of the NRF2 agonists by NRF2 luciferase reporter assay. **(C)** Screening of the KEAP1 binders by thermal shift assay. **(D)** The structure of CAD and its butyrylated derivative CAD-1. **(E)** The Nrf2 luciferase reporter activity induced by different concentrations of **CAD** and its derivative **CAD-1**.

Firstly, two complementary assays, including a cell-based NRF2 luciferase reporter assay to evaluate NRF2 agonistic activity, and a SYPRO Orange-based KEAP1 thermal shift assay to assess direct KEAP1 binding affinity, were used to identify novel KEAP1 binder from an in-house natural product library (>200 natural compounds). As shown in Fig. 1A-C, **CAD**, a naturally occurring chalcone, showing a 6.22-fold increase in NRF2 luciferase reporter assay (Fig. 1B and Table S1) and significant KEAP1 stabilization ability (as evidenced by a thermal shift (ΔTm) of +3.7 °C, Fig. 1C and Table S1). Subsequent surface plasmon resonance (SPR) analysis confirmed the binding affinity of **CAD** to KEAP1, showing a *K*_D_ value of 8.79 μM (Fig. S1A). Since the C-4 phenolic group of **CAD** is readily modifiable, a butyryl group was attached to this site. NRF2 luciferase reporter assays showed that this modification did not significantly alter the agonistic activity relative to **CAD**, suggesting that the C-4 position is suitable for further chemical derivatization (Fig. 1E). These findings suggest that **CAD** directly bind to KEAP1 and this agent bears a readily accessible modification site (C-4 phenolic group), supporting its utility as a scaffold for the development of KEAP1-targeting PROTACs.

**Fig. 2:**
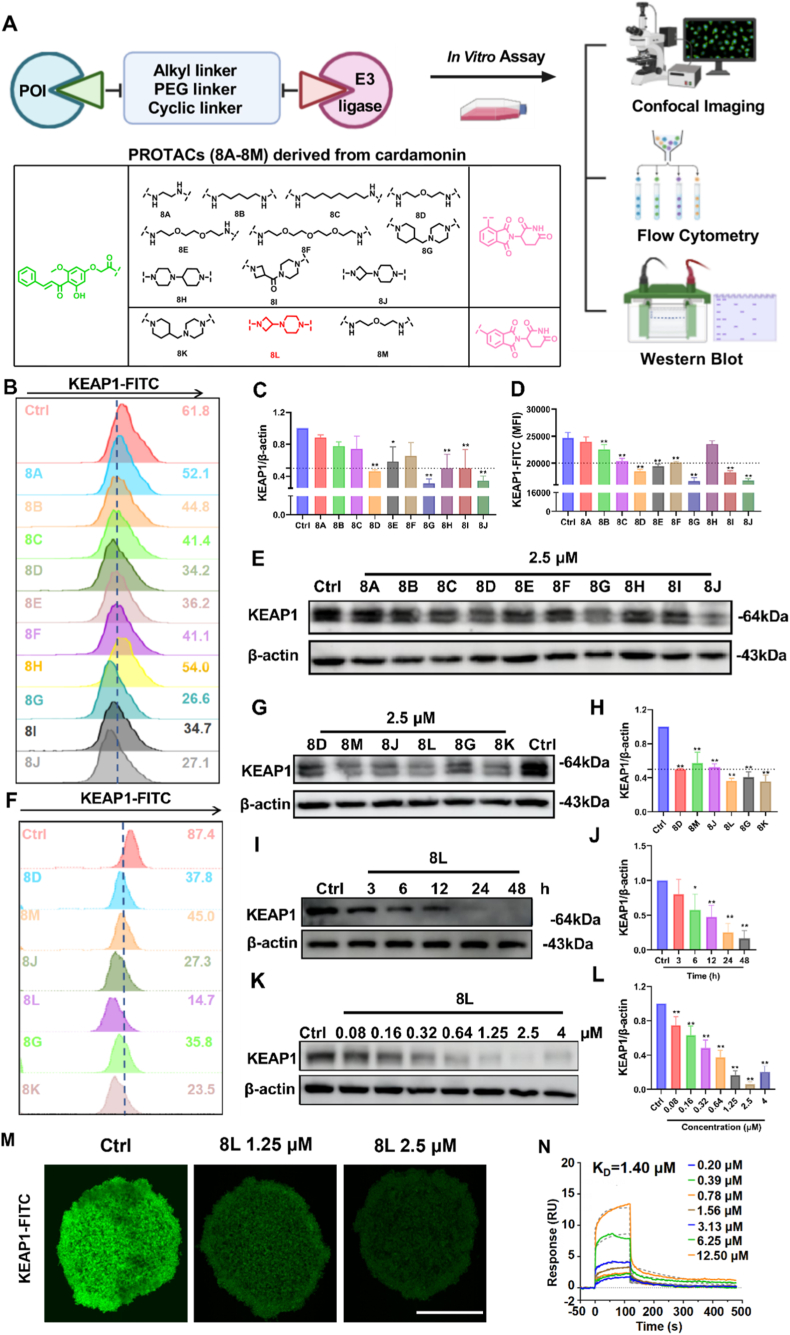
**Rational design of CAD-derived PROTACs and their KEAP1-degradation effect. (A)** Schematic of KEAP1 degrader design and discovery. **(B** and **C)** HepaRG cells were treated with various KEAP1-degrader candidates (**8A**-**J**), and the protein levels of KEAP1 were detected by flow cytometry, n = 3. **(D** and **E)** HepaRG cells were treated with various degrader candidates (**8A**-**J**) and the protein levels of KEAP1 were detected by Western blotting (WB), n = 3. **(F)** HepaRG cells were treated with the optimized degraders and the protein levels of KEAP1 were detected by flow cytometry. **(G** and **H)** HepaRG cells were treated with the optimized degraders and the protein levels of KEAP1 were detected by WB, n = 3. **(I** and **J)** HepaRG cells were treated with **8L** (2.5 μM) for 3, 6, 12, 24, and 48 h, n = 3. **(K** and **L)** HepaRG cells were treated with **8L** at different concentrations for 48 h, n = 3. (**M**) Immunofluorescence staining for KEAP1 (Green) in HepaRG 3D spheroids upon addition of **8L**, scale bar = 25 μm, n = 5. (**N**) Concentration-dependent SPR sensorgrams and equilibrium binding responses for **8L** binding to immobilized KEAP1. Note: Data are presented as mean ± SD, *∗P <* 0.05, *∗∗P <* 0.01.

Guided by docking simulations of the **CAD**-KEAP1 complex, a suite of **CAD**-derived PROTACs were then designed and synthesized, *via* introducing a variety of alkyl, polyethylene glycol, and nitrogen-containing heterocyclic linkers with different lengths at C-4 phenolic group of **CAD** coupling with pomalidomide, a widely used binder of the E3 ubiquitin ligase (Fig. 2A). In brief, **CAD** was initially functionalized with a carboxylic acid at the C-4 phenolic group to enable linker attachment (Scheme S1). Concurrently, a suite of E3 ligase binder featuring diverse linkers were synthesized *via* nucleophilic substitution of fluor-thalidomide with various N-Boc-protected diamines using DIEA as the base. The BOC protecting group was then removed under acidic conditions to get intermediates **7A-7J**, which were subsequently coupled with **CAD** derivative **3** to yield PROTACs **8A**-**8J** (Scheme S2). Considering that targeted *meta*-substitution of the Phthalimide Ring will minimize off-target degradation in Pomalidomide-Based PROTACs [38,39], PROTACs **8K**-**8M** were intentionally designed and synthesized for exploring the impact of different conjugation sites of E3 binder on KEAP1 degradation efficiency (Scheme S3).

### 3.2 **8L** shows high KEAP1-degradation effect in HepaRG cells

The KEAP1 degradation effects of the synthetic PROTAC candidates (**8A-8M**) were then tested using an integrated phenotypic assay including flow cytometry, confocal microscopy, and western blotting (WB) (Fig. 2A). The results demonstrated that the PROTACs with a short-chain polyethylene glycol linker (**8D**) or with nitrogen-containing heterocyclic linkers (**8G** and **8J)** exhibited high KEAP1 degradation potency, as evidenced by both flow cytometric quantification (Fig. 2B and C) and cross-validation through WB analysis (Fig. 2D and E). By contrast, other PROTACs with alkyl linkers (**8A**-**8C)** showed relatively weak KEAP1 degradation efficiency. To investigate the influence of substitution position on the phenyl ring of pomalidomide, three derivatives including **8K**, **8L**, and **8M,** were synthesized *via* substituting at the C-5 position of the E3 binder. Among them, the *meta*-substituted analog **8L** exhibited the most robust KEAP1 degradation efficacy (Fig. 2F–H and S1D). Meanwhile, no detectable cytotoxicity was observed for these PROTACs at 10 μM after 72 h of treatment in HepaRG cells (Fig. S1B and S1C).

To characterize the degradation profile of PROTAC **8L** on KEAP1, time- and dose-dependent KEAP1-depredating effects of **8L** were investigated in HepaRG cells. As shown in Fig. 2I–L and S1E, **8L**-induced KEAP1 degradation was gradually enhanced with increasing time and doses, showing a DC_50_ value of 227.2 nM and D_max_ of 90.5% at 2.5 μM in HepaRG cells. The degradation efficacy of **8L** was further validated across multiple hepatocyte cell lines, including the human hepatocellular carcinoma lines HepG2 and Huh7, as well as the mouse hepatocyte line AML-12. In AML-12 cells, **8L** induced robust, dose- and time-dependent degradation of KEAP1 protein, with the optimal effect observed at 5 μM, achieving 79% reduction after 48 h of treatment (Fig. S2A–2D). Similarly, **8L** (0.5 μM) exhibited potent degradation in AML-12 (87%) (Fig. S2E–2H) and Huh7 cells (90%) (Fig. S2I–2L).

To better mimic physiological liver tissue architecture, three-dimensional HepaRG spheroid cultures were established. **8L** retained its potent degradation capacity within this biomimetic model, displaying clear dose-response relationships (Fig. 2M and S1F**).** In addition, SPR analysis demonstrated that **8L** directly bound to KEAP1, with a dissociation constant (*K*_*D*_) of 1.40 μM, exhibiting a 6.28-fold higher binding affinity compared to **CAD** (Fig. 2N). These findings collectively confirm that **8L** directly engages KEAP1 and induces potent, consistent degradation in hepatocytes across species and culture models, supporting its potential as a promising KEAP1 degrader for further pharmacodynamic investigation.

### 3.3 **8L** degrades KEAP1 through the ubiquitin-proteasome system

**Fig. 3:**
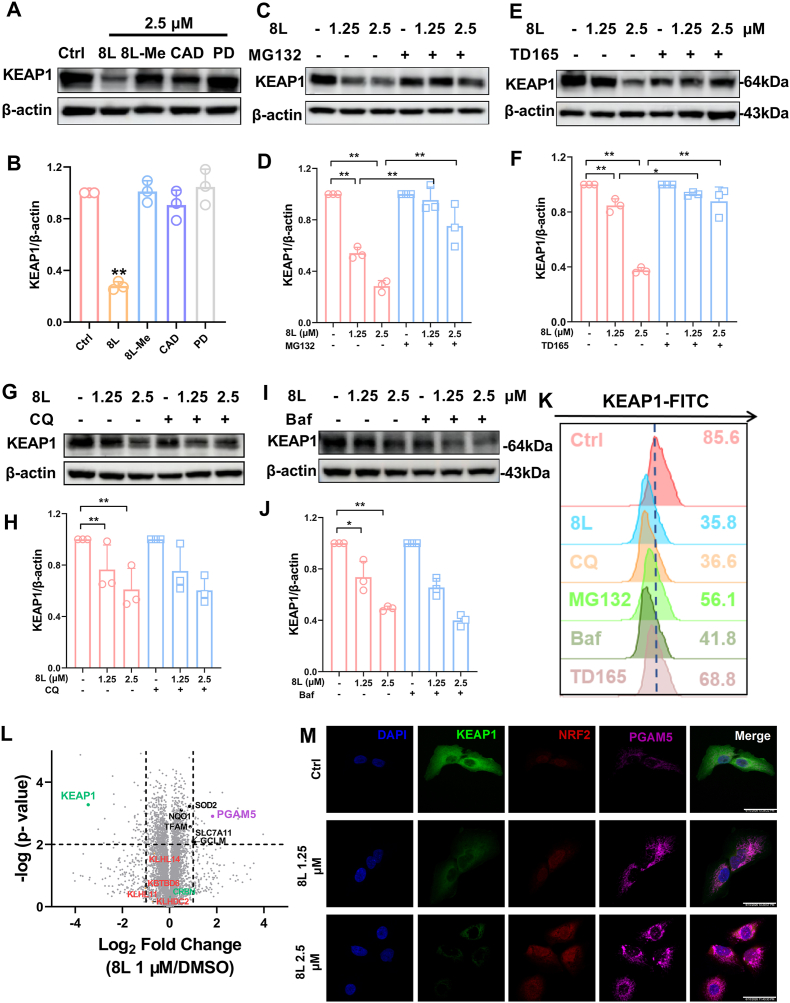
**Mechanism of PROTAC 8L-mediated KEAP1 degradation. (A** and **B)** WB analysis of KEAP1 and β-actin in HepaRG cells treated with 2.5 μM **8L**, **8L**-Me, **CAD**, or PD for 48 h and the relative levels of KEAP1. **(C and D)** HepaRG cells were treated with **8L** (1.25 or 2.5 μM) with or without proteasome inhibitor MG132 (10 μM). **(E** and **F)** HepaRG cells were treated with **8L** (1.25 or 2.5 μM) with or without CRBN degrader TD-165 (1 μM). **(G** and **H)** HepaRG cells were treated with **8L** (1.25 or 2.5 μM) with or without autophagy inhibitor CQ (20 μM). (**I** and **J**) HepaRG cells were treated with **8L** (1.25 or 2.5 μM) with or without autophagy inhibitor Baf (10 μM). **(K)** HepaRG cells were treated with **8L**, MG132, TD165, CQ and Baf, while the KEAP1 protein levels were detected by flow cytometry. **(L)** Volcano plots showing differentially expressed proteins of HepaRG cells following treatment with **8L** (1 μM). **(M)** Representative images of KEAP1 (green), NRF2 (red), and PGAM5 (purple) in HepaRG cells treated with different concentrations of **8L**, scale bar = 20 μm. Data are presented as mean ± SD, *∗P <* 0.05, *∗∗P <* 0.01, n = 3.

PROTAC-mediated target degradation requires a functional ubiquitin-proteasome system [40]. To elucidate the underlying mechanism of **8L**-induced KEAP1 depletion, a series of orthogonal pharmacological and genetic validations were performed. First, a warhead dependency experiment was conducted. As illustrated in Fig. 3A and B, 8L significantly reduced the protein levels of KEAP1 in HepaRG cells, whereas no noticeable KEAP1 degradation was observed upon exposure to either **8L**-Me (a methylated derivative at the secondary amine position of the thalidomide moiety of **8L**), or the parent binder **CAD**, or pomalidomide. These observations suggested that ternary complex formation was an essential process for **8L**-induced KEAP1 degradation. Notably, co-treatment with the proteasome inhibitor MG132 completely abrogated **8L**-induced KEAP1 degradation, confirming that the effect was dependent on ubiquitin-proteasome system (Fig. 3C and D). Furthermore, competition with TD-165, a cereblon-targeting PROTAC, partially rescued KEAP1 protein levels, demonstrating that **8L**-mediated degradation was cereblon-dependent (Fig. 3E and F).

Given that KEAP1 could also be degraded *via* autophagy, potentially through p62-mediated recruitment to autophagosomes [41], we next investigated whether autophagy was involved in **8L**-induced KEAP1 degradation. To test this, HepaRG cells were pre-treated with chloroquine (CQ) or bafilomycin A1 (Baf), the agent suppress early and late stages of autophagy, respectively [42,43]. The results showed that neither CQ or Baf prevented KEAP1 degradation (Fig. 3G-J), suggesting that p62-mediated autophagy was not involved in **8L**-induced KEAP1 degradation. These findings were further supported by flow cytometric analysis (Fig. 3K and S3A). In contrast, co-immunoprecipitation assays showed that **8L** significantly enhanced KEAP1 ubiquitination (Fig. S2M), confirming that **8L** promoted CRBN-mediated ubiquitination of KEAP1 and its subsequent proteasomal degradation.

Subsequently, label-free quantitative proteomics technology was employed to evaluate the specificity of **8L**-mediated KEAP1 degradation in hepatocytes. As shown in Fig. 3L and 8L specifically reduced the protein levels of KEAP1, leaving other Kelch domain-containing proteins (including KBTBD6, KLHDC2, KLHL14, KLHL11) unchanged. Notably, the protein levels of CRBN, another key component of the E3 ligase system, were unaltered. WB analysis verified that CRBN protein levels remained unchanged following treatment with 2.5 μM **8L** over time (Fig. S3C). The proteomic analysis also revealed a coordinated upregulation of NRF2 downstream targets (NQO1, GCLC, SLC7A11, TFAM, SOD2), which was further supported by qRT-PCR analysis showing that the mRNA levels of *HO-1*, *NQO1*, *GCLC*, and *GCLM* in HepaRG cells were correspondingly increased upon treatment with varying concentrations of **8L** (Fig. S3D). Notably, PGAM5, the another known KEAP1-associated protein, was also found to be upregulated in the proteomic dataset [19]. Immunofluorescence imaging confirmed the dose-dependent KEAP1 depletion and concomitant upregulation of both NRF2 and PGAM5 (Fig. 3M and S3B). These findings clearly demonstrate that **8L** selectively depletes KEAP1 *via* the ubiquitin-proteasome pathway, leading to the simultaneous activation of NRF2 and PGAM5 signaling pathways in hepatocytes.

### 3.4 **8L** significantly mitigates drug-induced hepatocyte injury

**Fig. 4:**
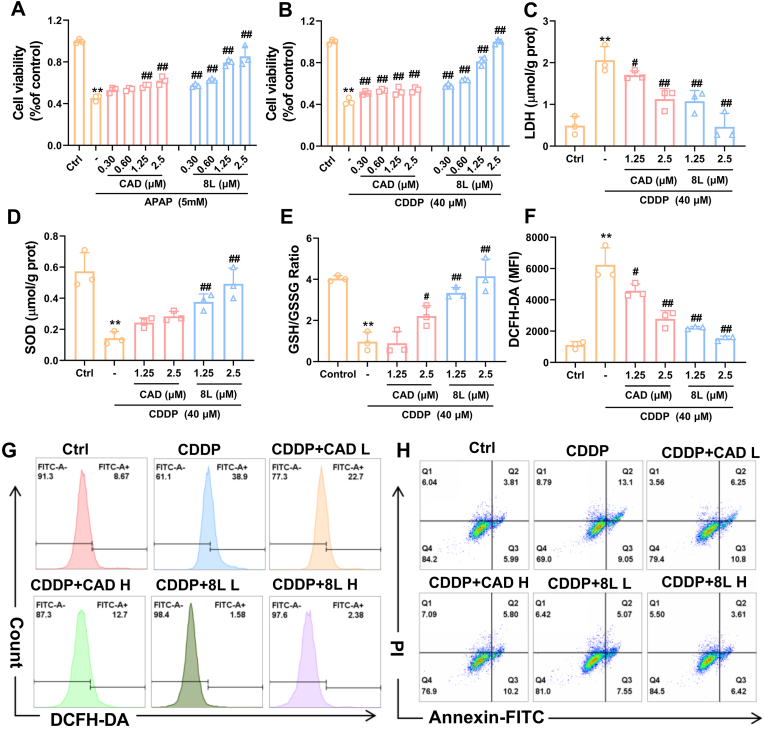
**8L mitigates drug-induced cellular oxidative stress and cell damage in HepaRG cells.** HepaRG cells are pre-treated with or without **8L** or **CAD** for 24 h, and then treated with APAP (10 mM) or CDDP (40 μM) for additional 24 h. **(A** and **B)** Assessment of cell viabilities using the CCK-8 assay, Date in A and B, n = 3. **(C**–**E)** Cellular levels of LDH, GSH/GSSG, and SOD were quantified using commercially available assay kits, n = 3. **(F** and **G)** The levels of ROS in HepaRG cells are measured by flow cytometry, n = 3. **(H)** The percentage of apoptosis cells in different groups are monitored by flow cytometry, n = 3. Note: **CDDP** (40 μM); **CAD** L (1.25 μM); **CAD** H (2.5 μM); **8L** L (1.25 μM); **8L** H (2.5 μM). Data are presented as mean ± SD, *∗P* < 0.05, *∗∗P* < 0.01 *vs.* control group; ^*#*^*P* < 0.05, ^*##*^*P* < 0.01 *vs.* CDDP group.

Considering that oxidative stress represents a pivotal driver in DILI pathogenesis, we subsequently investigate the protective efficacy of **8L** and **CAD** against drug-induced hepatocyte injury using CDDP and APAP as clinically prevalent hepatotoxicants [44]. As shown in Fig. 4A and B, 8L demonstrated superior efficacy in mitigating both CDDP and APAP-induced hepatocyte injury when compared to **CAD**. Building on prior evidence of KEAP1 degraders for alleviating APAP-induced liver injury [33], we further validated the protective effects of **8L** against CDDP-induced hepatocyte injury. As expected, **8L** markedly suppressed LDH leakage, while strongly enhanced the antioxidative defenses *via* elevating the GSH/GSSG ratio and SOD activity (Fig. 4C-E), and suppressing intracellular ROS accumulation in CDDP-treated hepatocytes, as revealed by flow cytometry (Fig. 4F and G), which was corroborated by parallel reductions in apoptotic signaling (Fig. 4H and S4A). These findings demonstrate that **8L** shows robust hepatoprotective effects against drug-induced hepatocyte injury by attenuating oxidative stress, enhancing antioxidant capacity. *3.5* ***8L*** *repairs the MQC system in CDDP-challenged hepatocytes by boosting NRF2 and PGAM5 pathways.*

**Fig. 5:**
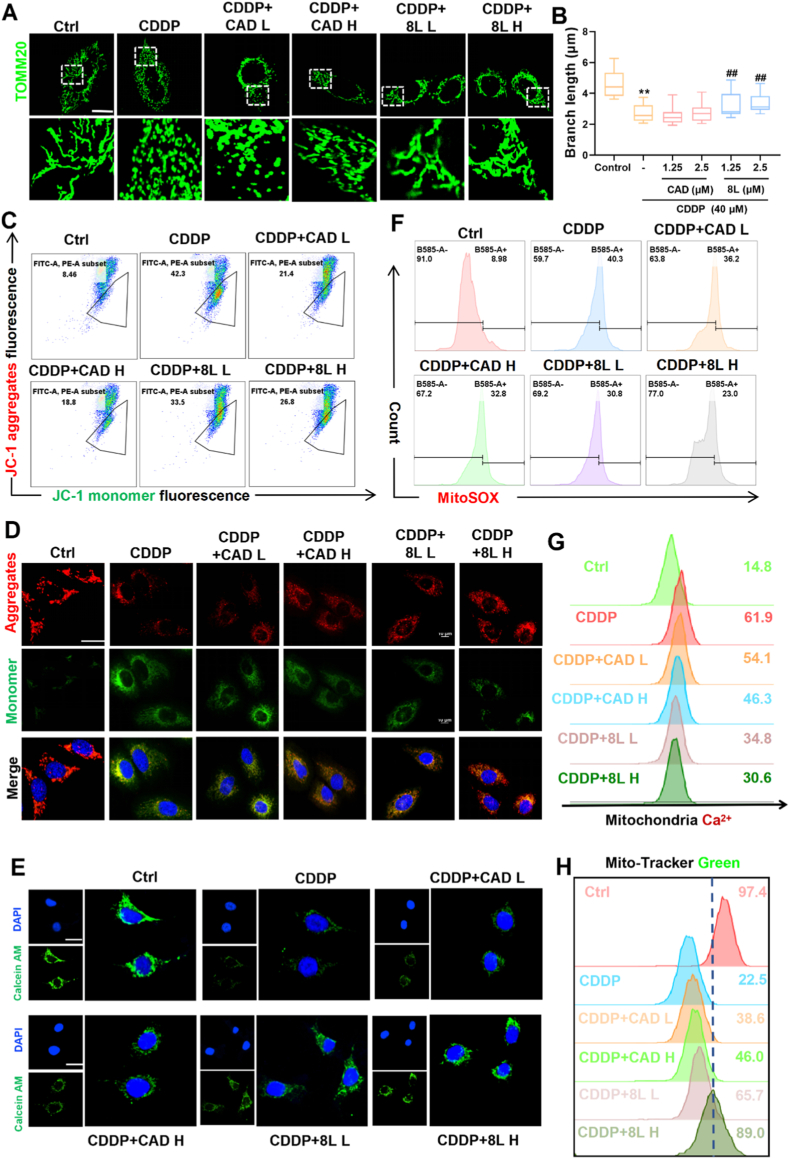
**8L mitigates drug-induced hepatocyte injury by activating the MQC system. (A** and **B)** Representative IF images of TOMM20 (green) in HepaRG cells, scale bar = 10 μm. **(C)** The mitochondrial membrane potentials among different groups were measured by flow cytometry using a JC-1 probe, n = 3. **(D)** Mitochondrial membrane potential was measured in different groups *via* JC-1-based confocal imaging, scale bar = 25 μm, n = 3. **(E)** Representative fluorescence images of calcein AM/Co^2+^ quencher staining, scale bar = 25 μm, n = 3. **(F)** The mtROS levels are examined by flow cytometry with MitoSOX in HepaRG cells, n = 3. **(G)** The Ca^2+^ levels among different groups, which are examined by flow cytometry, n = 3. **(H)** The mitochondrial mass in HepaRG cells by mitoTracker green staining, n = 3. Note: **CDDP** (40 μM); **CAD** L (1.25 μM); **CAD** H (2.5 μM); **8L** L (1.25 μM); **8L** H (2.5 μM). Data are presented as mean ± SD, *∗P* < 0.05, *∗∗P* < 0.01 *vs.* control group; ^*#*^*P* < 0.05, ^*##*^*P* < 0.01 *vs.* CDDP group.

Given the established role of impaired mitochondrial quality control (MQC) in DILI pathogenesis [45], we subsequently investigated whether **8L** could restore mitochondrial integrity in CDDP-challenged hepatocytes. Unlike the elongated networks in control hepatocytes, mitochondria in CDDP-treated hepatocytes became fragmented and punctate, while this damage could be markedly ameliorated by **8L** (Fig. 5A and B). Furthermore, **8L** was significantly more effective than **CAD** in reversing CDDP-induced mitochondria fragmentation. Functional assays demonstrated that **8L** preserved mitochondrial membrane potential (Fig. 5C and D and S5A), suppressed mitochondrial permeability transition pore opening (Fig. 5E), and reduced the leakage of both mtROS (Fig. 5F and S5D**)** and Ca^2+^ (Fig. 5G and S5C) in CDDP-challenged hepatocytes. It was also found that **8L** significantly restored CDDP-induced mitochondrial dysfunction by rescuing mtDNA synthesis (Fig. S5E), alleviating ATP depletion (Fig. S5F), and improving mitochondrial quality (Fig. 5H and S5B). This functional recovery was accompanied by enhanced mitophagy flux, evidenced by increased autophagosome formation (red) and mitochondrial-lysosomal colocalization (green) in HepaRG cells (Fig. S5G and S5H**)**. These findings clearly demonstrate that **8L** effectively counteracts CDDP-induced hepatocyte injury through a multi-faceted enhancement of MQC.

**Fig. 6:**
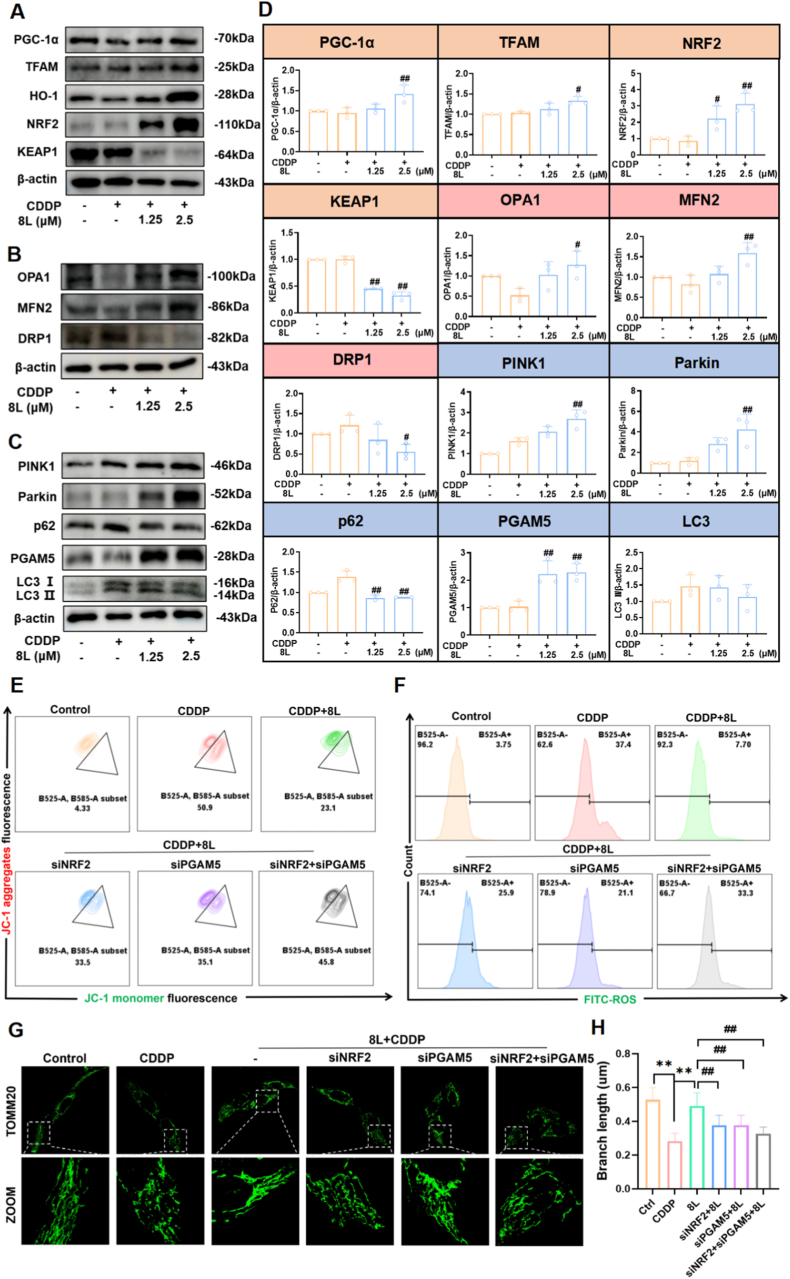
**8L mitigates drug-induced hepatocyte injury by boosting both NRF2 and PGAM5 signaling pathways. (A**–**C)** The protein levels of NRF2, PGC-1α, TFAM, HO-1, KEAP1, PINK1, Parkin, P62, PGAM5, LC3 Ⅰ/Ⅱ, MFN2, OPA1 and Drp1 in HepaRG cells, n = 3. **(D)** The relative protein levels of NRF2, PGC-1α, TFAM, HO-1, KEAP1, PINK1, Parkin, P62, PGAM5, LC3 Ⅰ/Ⅱ, MFN2, OPA1 and Drp1, n = 3. **(E)** Mitochondrial membrane potential was measured in different groups *via* JC-1-based flow cytometry, **8L** (2.5 μM), n = 3. **(F)** The levels of ROS in HepaRG cells are measured by flow cytometry, **8L** (2.5 μM), n = 3. (**G-H**) Representative IF images of TOMM20 (green) in HepaRG cells, scale bar = 10 μm, **8L** (2.5 μM). Note: **CDDP** (40 μM), data are presented as mean ± SD, *∗P* < 0.05, *∗∗P* < 0.01 *vs.* control group; ^*#*^*P* < 0.05, ^*##*^*P* < 0.01 *vs.* CDDP group.

In CDDP-challenged HepaRG cells, **8L** enhanced MQC by depleting KEAP1 and upregulating NRF2 and PGAM5, thereby mitigating CDDP-induced oxidative stress. As expected, **8L** upregulated PGC-1α and TFAM, with a significant effect at the high dose and a limited effect at the low dose, which enhanced mitochondrial biogenesis (Fig. 6A and D), restored fission/fusion balance by suppressing DRP1, as well as rescuing OPA1/MFN2 expression disrupted by CDDP (Fig. 6B and D). The results demonstrate that **8L** activates the PINK1/Parkin signaling pathway through dual upregulation of NRF2 and PGAM5 expression, which not only significantly enhances mitophagy but also effectively eliminates damaged mitochondria while reducing abnormal accumulation of p62/LC3-II (Fig. 6C and D). These findings demonstrate that **8L** counteracts CDDP-challenged mitochondrial impairment by coordinately activating both NRF2- and PGAM5-mediated MQC restoration, offering a multi-targeted therapeutic strategy against DILI.

To further delineate the underlying mechanisms, siRNA-based experiments were performed. Individual knockdown of either NRF2 or PGAM5 partially attenuated, but did not abolish, the protective effects of **8L** against CDDP-induced mitochondrial impairment, including loss of membrane potential (Fig. 6E and S5D) and elevated ROS production (Fig. 6F and S6C). In contrast, dual knockdown of both NRF2 and PGAM5 largely reversed the mitochondrial integrity conferred by **8L** (Fig. 6G and H). These observations suggest that NRF2 and PGAM5 function interdependently to mediate the hepatoprotective and mitochondrial-stabilizing actions of **8L**.

### 3.5 **8L** shows high safety profiles and liver-preferential properties

**Fig. 7:**
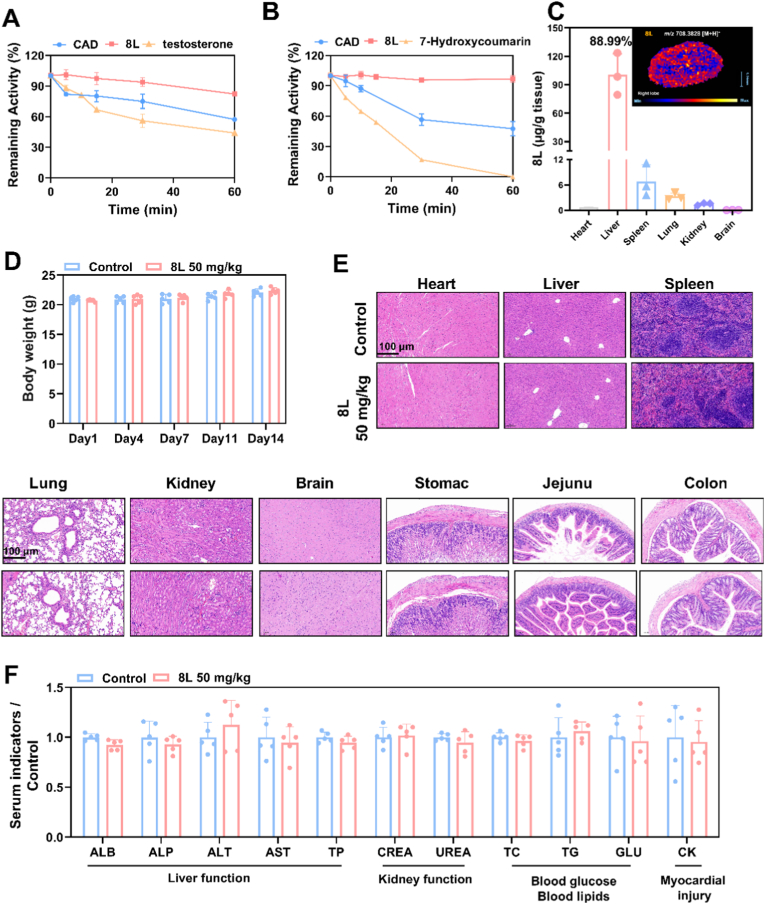
**8L shows good safety profiles and liver-preferential properties. (A)** Metabolic stability of **8L** and **CAD** in phase I metabolic system, testosterone was used as positive control. **(B)** Metabolic stability of **8L** and **CAD** in phase II glucuronidation system, while 7-hydroxycoumarin was used as positive control. **(C)** Tissue distribution of **8L** in various organs of mice following intraperitoneal administration of **8L** (50 mg/kg/day) for 7 consecutive days, n = 3. **(D)** Body weight changes in control and **8L**-treated mice, n = 5. **(E)** Histopathological assessment of organ tissues from mice receiving **8L** (50 mg/kg/day) *via* intraperitoneal injection for 14 days (scale bar = 100 μm). **(F)** The serum biochemical parameters of control and **8L**-treated mice, n = 5.

Favorable druggability and safety profiles are essential prerequisites for drug development [46]. In this work, the metabolic stability of **8L** was examined in human liver microsomes under both phase I (NADPH-supplemented) and phase II (UDPGA-supplemented) metabolic systems. As shown in Fig. 7A and B, 8L exhibited markedly superior metabolic stability compared to **CAD** and the positive control compounds (testosterone and 7-hydroxycoumarin) in both phase I and phase II metabolic systems. These findings suggested that **8L** showed superior metabolic stability. Interestingly, tissue distribution studies revealed that **8L** exhibited distinct hepatic accumulation. Following intraperitoneal administration of **8L** (50 mg/kg), the hepatic exposure levels of **8L** reached approximately 100 μg/g tissue (88.99% of total distribution), which was over 14-fold higher than that in the spleen and other tested organs (Fig. 7C). Consistent with these findings, mass spectrometry imaging further confirmed robust and localized distribution of **8L** within liver tissue (Fig. S7).

**Fig. 8:**
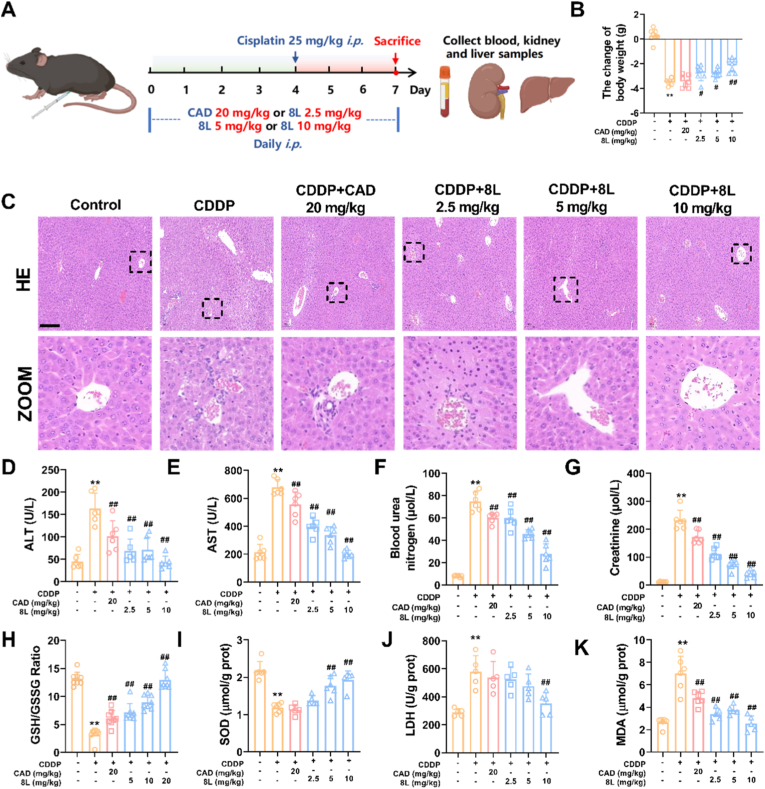
**8L mitigates CDDP-induced DILI in C57BL/6J mice. (A)** A schematic diagram of the animal study. **(B)** The changes in body weight of mice from different groups. **(C)** Histopathological changes of the liver among different groups. Scale bar = 100 μm. **(D**–**G)** Serum levels of ALT, AST, creatinine and blood urea nitrogen among different groups. **(H–K)** The levels of GSH/GSSG, SOD, LDH and MDA in liver tissues collected from different groups. Note: Data are presented as mean ± SD, *∗P* < 0.05, *∗∗P* < 0.01 *vs.* control group; ^*#*^*P* < 0.05, ^*##*^*P* < 0.01 *vs.* CDDP group, n = 8.

Given that preclinical-stage degraders are often limited by excessive toxicity, the safety profile of **8L** was further evaluated in healthy mice. Intraperitoneal administration of **8L** at a high dose (50 mg/kg/day) for 14 consecutive days showed no mortality and negligible changes in body weight (Fig. 7D). Histopathological evaluation of major organs (liver, kidney, heart, etc.) revealed no apparent toxicity (Fig. 7E). Additionally, serum biomarkers including ALT, AST, ALP, and triglycerides, showed no significant alterations (Fig. 7F). The preservation of normal biochemical indices and tissue morphology collectively indicated an excellent safety profile for **8L** in preclinical settings. Collectively, the suitable metabolic stability, liver-preferential distribution, and high safety profiles of **8L** support its continued *in vivo* tests.

### 3.6 **8L** protects against CDDP- and APAP-induced DILI *in vivo*

**Fig. 9:**
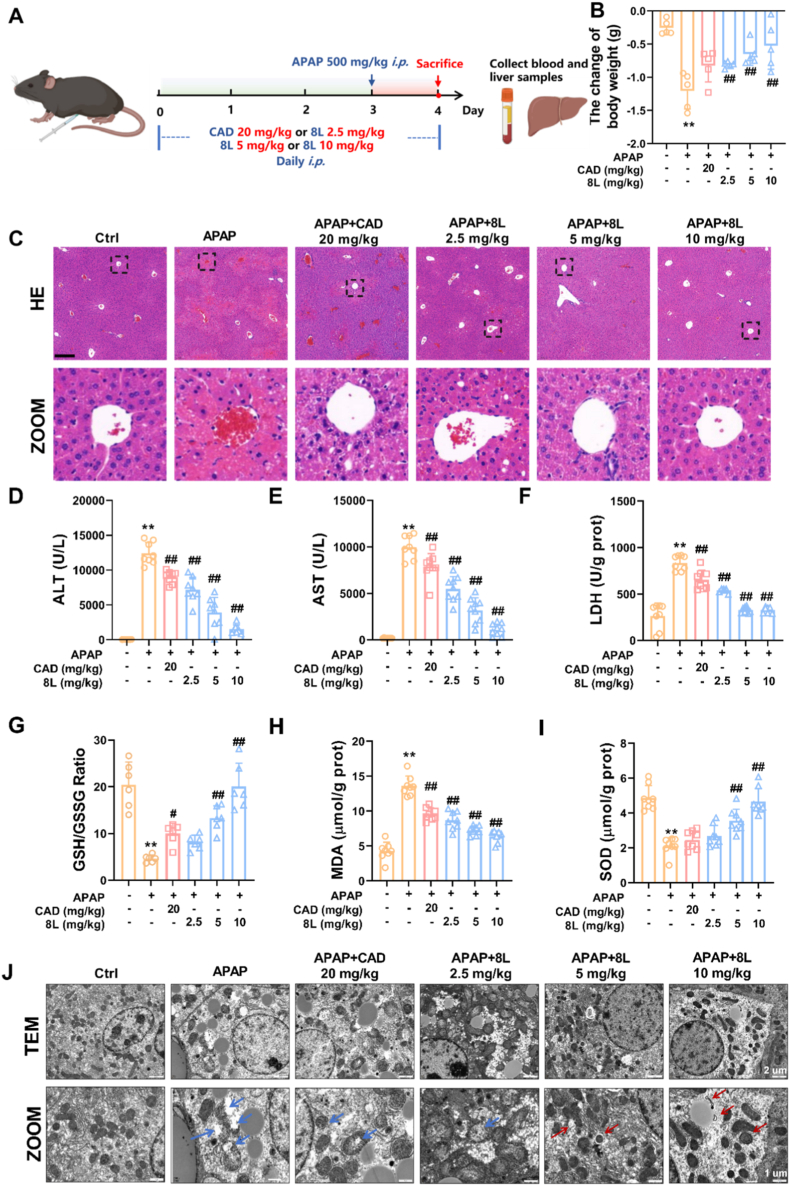
**8L mitigates APAP-induced DILI in C57BL/6J mice. (A)** A schematic diagram of the animal study. **(B)** The body weight of mice from different groups. **(C)** Histopathological changes of the liver among different groups. Scale bar = 100 μm. **(D**–**E)** Serum levels of ALT and AST among different groups. **(F–I)** The levels of LDH, GSH/GSSG, MDA and SOD in liver tissues collected from different groups. **(J)** Representative transmission electron microscopy images of liver tissues from mice in different groups. Blue arrow: Damaged and swollen mitochondria, red arrow: Mitochondria undergoing mitophagy, scale bar = 2 and 1 μm. Note: Data are presented as mean ± SD, *∗P* < 0.05, *∗∗P* < 0.01 *vs.* control group; ^*#*^*P* < 0.05, ^*##*^*P* < 0.01 *vs.* APAP group, n = 8.

Next, the *in vivo* therapeutic effects of **8L** against DILI were tested in both CDDP- and APAP-challenged mouse models at three doses (2.5, 5, and 10 mg/kg/day). As shown in Fig. 8A and B, CDDP-challenged mice exhibited significant body weight loss compared to controls at 72 h post-injection, which could be dose-dependently reversed by **8L**. Histopathological evaluation revealed that CDDP induced marked structural damage in both liver and kidney tissues. In contrast, **8L** dose-dependently preserved tissue architecture and mitigated CDDP-induced histological alterations, including hepatocyte damage, inflammatory cell infiltration, and sinusoidal disorganization in the liver (Fig. 8C), as well as hyaline cast formation, tubular necrosis, and inflammatory cell infiltration in the kidney (Fig. S8B). Biochemical assays showed that CDDP markedly elevate the serum levels of ALT, AST, creatinine, and urea, while **8L** significantly attenuated these hepatotoxic and nephrotoxic signs at all tested doses (Fig. 8D-G). Furthermore, **8L** significantly restored antioxidant defenses, including GSH/GSSG and SOD ratio, in both liver (Fig. 8H and I) and kidney tissues (Fig. S8D and S8F). Consistently, **8L** markedly suppressed CDDP-induced oxidative stress markers, including ROS, LDH, and MDA, in liver (Figs. S8A, 8J and 8K) and kidney tissues (Figs. S8B, S8C and S8E). Consistent with these findings, **8L** conferred broad protective benefits in APAP-induced hepatotoxic mice. As shown in Fig. 9, Fig. 8L significantly mitigated APAP-induced hepatotoxicity by reversing body weight loss (Fig. 9B), ameliorating APAP-induced histopathological liver injury, including hepatocyte swelling and spotty or confluent necrosis (Fig. 9C), suppressing ALT and AST elevation (Fig. 9D and E), reducing oxidative stress (such as the levels of LDH, MDA), and restoring antioxidant defenses (SOD, GSH) (Fig. 9F-I). Moreover, transmission electron microscopy revealed that **8L** treatment induced mitophagy *in vivo*, as evidenced by autophagic vesicles engulfing mitochondria, and effectively rescued APAP-induced ultrastructural damage, including mitochondrial rupture, cristae loss, and reduced mitochondrial number (Fig. 9J). These results clearly demonstrate that **8L** effectively mitigates DILI in both CDDP- and APAP-challenged mouse models by restoring redox homeostasis and preserving hepatic integrity.

### 3.7 **8L** mitigates DILI by boosting both NRF2 and PGAM5 pathways *in vivo*

**Fig. 10:**
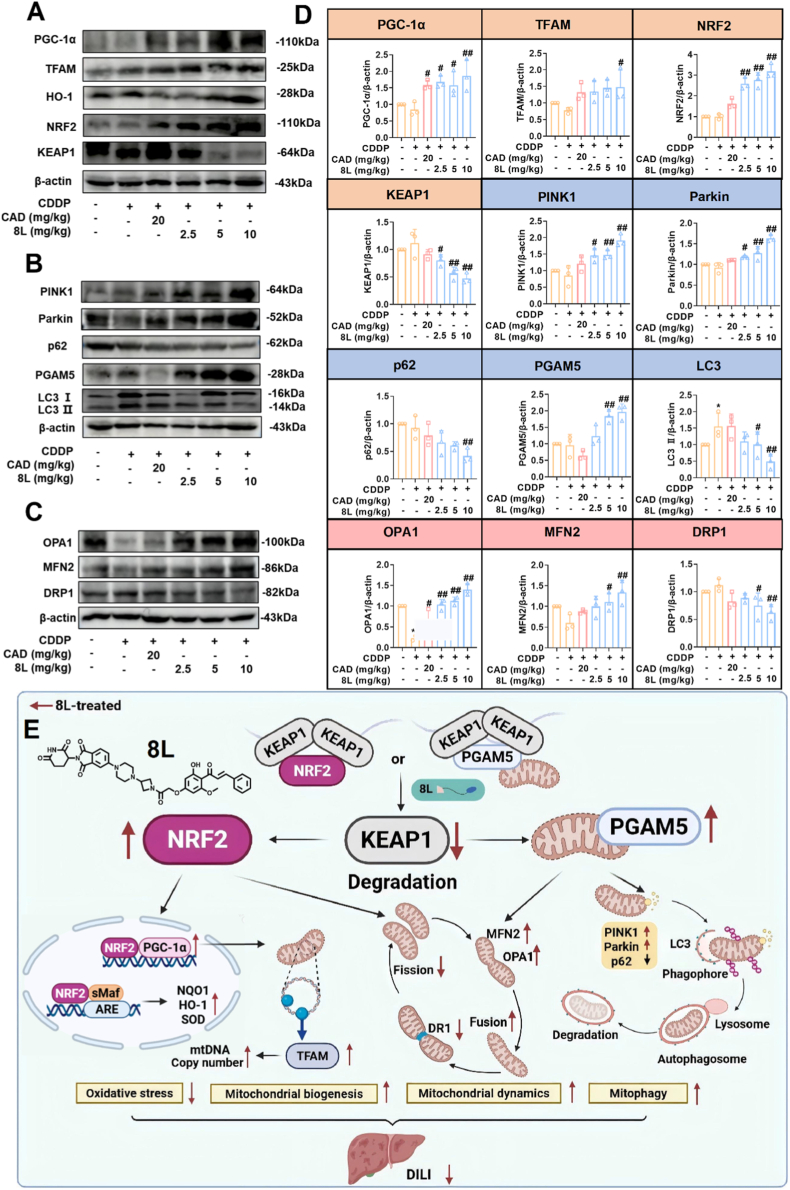
**8L mitigates DILI by boosting both NRF2 and PGAM5 signaling pathways. (A**–**C)** The protein levels of NRF2, PGC-1α, TFAM, HO-1, KEAP1, PINK1, Parkin, P62, PGAM5, LC3 Ⅰ/Ⅱ, MFN2, OPA1 and Drp1 in liver tissue **(D)** The relative levels of NRF2, PGC-1α, TFAM, HO-1, KEAP1, PINK1, Parkin, P62, PGAM5, LC3 Ⅰ/Ⅱ, MFN2, OPA1 and Drp1, n = 3. **(E)** Schematic illustration of the dual mechanisms of **8L** for mitigating drug-induced mitochondrial dysfunction and oxidative stress. Note: Data are presented as mean ± SD, *∗P* < 0.05, *∗∗P* < 0.01 *vs.* control group; ^*#*^*P* < 0.05, ^*##*^*P* < 0.01 *vs.* CDDP group.

To further elucidate the underlying mechanisms of **8L**-mediated hepatoprotection, the key proteins involved in NRF2 and PGAM5 signaling pathways were examined in liver tissues collected from various groups. As shown in Fig. 10A and D, **8L** dose-dependently downregulated KEAP1 while upregulated NRF2/HO-1 and PGAM5. Furthermore, **8L** significantly enhanced the expression of mitochondrial biogenesis markers including PGC-1α and TFAM (Fig. 10A and D). Mitophagy related proteins, such as PINK1, Parkin, p62, and LC3, were also upregulated in a dose-dependent manner consistent with *in vitro* findings (Fig. 10B and D). It was also observed that the mitochondrial dynamics regulators (such as Drp1, OPA1, and MFN2) could be significantly restored by **8L** (Fig. 10C and D). These molecular alterations in mice liver closely mirrored those observed in the cellular model. Collectively, these results demonstrate that **8L** exerts robust protective effects against both CDDP- and APAP-induced liver injury by restoring redox homeostasis and preserving hepatic integrity through coordinated enhancement of mitochondrial biogenesis, mitophagy, and dynamics.

### 3.8 Liver-specific KEAP1 knockout abrogates the hepatoprotective effects of **8L** in DILI mice

To further validate the KEAP1 dependence of the therapeutic effects of **8L** against DILI, we established an APAP-induced DILI model using liver-specific KEAP1 knockout (Hep^cre^-*KEAP1*^*flox/flox*^) mice. The results showed that APAP significantly decreased the body weight and increased serum levels of both ALT and AST in Hep^cre^-*KEAP1*^*flox/flox*^ mice, while **8L** failed to attenuate these hepatoxic indicators (Fig. S10A–S10C). Moreover, APAP could trigger marked hepatocyte swelling and nuclear pyknosis in Hep^cre^-*KEAP1*^*flox/flox*^ mice, while **8L** failed to ameliorate these histopathological changes significantly (Fig. S10D). Consistently, **8L** hardly restore the levels of antioxidant biomarkers (SOD and GSH) in Hep^cre^-*KEAP1*^*flox/flox*^ mice (Fig. S10E and S10F), and could notreduce APAP-induced oxidative damage markers (LDH and MDA) significantly (Fig. S10G and S10H). These findings clearly demonstrate that the hepatoprotective effect of **8L** is strictly dependent on the hepatic KEAP1, suggesting that KEAP1 played a crucial role in **8L**-mediated hepatoprotective effects (Fig. 10E).

## 4 Discussion

Among all drug-induced diseases, DILI represents a major clinical challenge due to its unpredictable onset, complex multifactorial etiology, and lack of effective therapeutics. The KEAP1-NRF2 signaling pathway plays a central role in cellular defense against oxidative stress, making its activation a promising therapeutic strategy for oxidative stress-related disorders, including DILI [47]. Covalent modification on the functional cysteine residues of KEAP1 using electrophilic small-molecule agents is the most common strategy to activate the KEAP1-NRF2 pathway. However, the previously reported covalent agents of KEAP1 offer suffer from off-target effects, poor liver-enriched capability, resulting in a narrow therapeutic window and significant safety concerns. To overcome these limitations, this work utilized a dual-action strategy to we developed a novel mechanistically-elucidated and liver-enriched PROTAC of KEAP1 (termed **8L**), aiming to mitigate DILI *via* dual activation of the NRF2 and PGAM5 signaling pathways.

Unlike conventional small-molecule agents, PROTAC technology represents an emerging breakthrough strategy designed to eliminate the protein of interest, offering some inherent advantages including sustained pharmacology, enhanced specificity, minimal off-target effects, and pronounced liver-targeting capability. In this study, **CAD** was as a naturally occurring KEAP1-binder. However, as a classical electrophilic small molecule, **CAD** could interact with a variety of proteins in living cells [48], raising significant safety concerns. To transform this non-selective binder into a selective degrader of hepatic KEAP1, we utilized **CAD** as a KEAP1-binding binder to develop a series of KEAP1 PROTACs through coupling with pomalidomide (an E3 ligase binder) using a series of rationally designed linkers. It is well-known that linker composition profoundly influences the degradation efficacy and selectivity of PROTACs [49]. In this work, our results clearly demonstrated that **8L** (featured a nitrogen-containing heterocyclic linker attached at the *meta*-position of the E3 binder) showed improved degradation efficiency and high specificity compared to the PROTACs bearing alkyl or PEG linkers. These observations are highly consistent with recent studies [36], which highlight the effects of rigid heterocyclic linkers to enhance proteolytic efficiency and reduce off-target effects. Importantly, **8L**‐induced KEAP1 degradation was confirmed to be CRBN-dependent and proteasome-mediated, which distinct from autophagy-mediated KEAP1 degradation.

The advantages of **8L** over its parent compound (**CAD**) are multifaceted. Unlike **CAD**, **8L** exhibits enhanced KEAP1 binding affinity and provides sustained NRF2 activation. More importantly, **8L** overcomes the fundamental limitation of **CAD**'s single mode of action. As a classic KEAP1 binder, **CAD** only induces transient NRF2 pathway activation. In contrast, **8L** showed exceptional liver-preferential properties and sustained degrading activity towards KEAP1, while **8L**-mediated KEAP1 degradation could simultaneously activate both NRF2 and PGAM5 signaling pathways, thereby offering a broader therapeutic mechanism. These findings suggest that targeted degradation of KEAP1 could synergistically regulate multiple pathways orchestrated by this key protein [50]. Furthermore, the liver expresses a variety of E3 ubiquitin ligases at extremely high levels [51]. creating an ideal environment for **8L** to exert its potent hepatoprotective effects. Compared to conventional small-molecule agents (MW < 500), PROTACs possess substantially higher molecular weights and larger sizes. These properties promote liver accumulation and reduce susceptibility to hepatic metabolism, effectively explains the liver-preferential distribution observed in PROTACs, including **8L**.

Notably, the newly developed KEAP1 degrader **8L** could simultaneously activate NRF2-driven antioxidant transcription and stabilize PGAM5, providing a dual enhancement of redox balance and mitochondrial quality control. Our findings demonstrate that KEAP1 degradation simultaneously upregulates NRF2 and PGAM5, thereby promoting mitophagy, improving mitochondrial dynamics, and restoring bioenergetics, which is in line with recent studies implicating the KEAP1-PGAM5 complex in stress-induced mitophagy [19]. In our research, **8L** is KEAP1-directed PROTAC shown to dually activate both pathways. Notably, dual knockdown of NRF2 and PGAM5 was required to fully reverse **8L**-mediated mitochondrial stabilization and oxidative stress mitigation, underscoring their cooperative function.

The robust *in vivo* efficacy of **8L** in both APAP- and CDDP-induced DILI models, together with its favorable safety profile, strongly supports its translational potential. Current management of DILI is limited to discontinuation of the culprit drug and administration of N-acetylcysteine. However, N-acetylcysteine exerts only partial efficacy in APAP-induced DILI, with no specific therapeutic strategies targeting the core pathology of oxidative stress-mitochondrial injury nor liver-enriched capability [52]. Treatment with **8L** consistently attenuated drug-induced hepatic damage by lowing serum transaminase levels, improving histopathological alterations, and enhancing cellular antioxidant capacity, collectively highlighting its strong therapeutic activity. Furthermore, the marked preferential liver distribution of **8L** ensures high local drug exposure at the site of injury while minimizing distribution to non-target organs, thereby reducing the risk of systemic and off-target toxicities. This tissue-selective distribution profile is particularly beneficial for the chronic or repetitive dosing regimens required in the clinical management of DILI, positioning **8L** as a promising candidate for further drug development.

However, this study has several limitations that merit further investigation. First, the label-free quantitative proteomics study revealed that a few other proteins such as DNAJC14 were also downregulated by **8L**, suggesting uncharacterized mechanism of action potential for **8L**, or possible off-target effects [53]. Second, to advance its translational potential, additional studies are needed to validate the hepatoprotective effects of **8L** and establish dose-response relationships using human-relevant models, such as patient-derived primary hepatocytes. Finally, **8L**'s liver-preferential distribution is advantageous for chronic DILI treatment, making it a promising candidate for clinical development. Evaluating **8L** in other liver injury models, such as alcohol-induced hepatitis or metabolic dysfunction-associated steatohepatitis, could further clarify its broader therapeutic potential.

## 5 Conclusion

In summary, a dual-action strategy was adapted to develop a novel KEAP1 PROTAC for mitigating DILI through targeted degradation of hepatic KEAP1. The PROTAC **8L** was rationally designed by leveraging the identified natural KEAP1 binder (**CAD**) and an optimized linker to achieve selective, proteasome-dependent degradation of KEAP1. Compound **8L** shows exceptional liver-exposure ability and high degradation specificity, enabling both sustained, tunable activation of the NRF2-ARE pathway and concurrent PGAM5 stabilization to restore mitochondrial homeostasis. This dual regulatory mechanism robustly enhances mitochondrial biogenesis, maintains dynamic balance, and promotes efficient mitophagic clearance in hepatocytes. The dual regulation of redox balance and mitochondrial homeostasis endows **8L** as an efficacious hepatoprotective agent against both CDDP- and APAP-induced liver injury across *in vitro* and *in vivo* models. Overall, our findings not only position **8L** as a promising bifunctional therapeutic agent for DILI, but also validate targeted degradation of hepatic KEAP1 as a dual-regulatory strategy to mitigate liver disorders triggered by redox imbalance and mitochondrial dysfunction.

## CRediT authorship contribution statement

**Yanyan Deng:** Conceptualization, Data curation, Formal analysis, Funding acquisition, Investigation. **Leizhi Xu:** Conceptualization, Data curation, Formal analysis, Funding acquisition. **Xiaoting Niu:** Conceptualization, Data curation. **Jingjing Li:** Conceptualization, Data curation. **Yuan Xiong:** Conceptualization. **Guanghao Zhu:** Conceptualization, Data curation. **Zhiyi Lu:** Conceptualization, Data curation. **Chuting Xu:** Conceptualization, Data curation. **Xuerui Wang:** Conceptualization. **Pu Wang:** Data curation. **Jian Huang:** Formal analysis. **Zhangping Xiao:** Conceptualization. **Frank J. Gonzalez:** Formal analysis. **Lili Ji:** Methodology. **Caixia Sun:** Data curation, Formal analysis. **Ping Wang:** Conceptualization, Data curation. **Guangbo Ge:** Conceptualization, Data curation.

## Declaration of competing interest

The authors declare that they have no known competing financial interests or personal relationships that could have appeared to influence the work reported in this paper.

## Data Availability

Data will be made available on request.

## Footnotes

Appendix A Supplementary data to this article can be found online at https://doi.org/10.1016/j.redox.2026.104296.
