# Supplementary material for: Targeted degradation of hepatic KEAP1 mitigates drug-induced liver injury via dual boosting NRF2 and PGAM5 signaling
Source: Redox Biol. 2026 Jul 11;95:104296. doi: 10.1016/j.redox.2026.104296 (PMC13382280; doi:10.1016/j.redox.2026.104296)

**Supplementary information**

*for*

**Targeted Degradation of Hepatic KEAP1 Mitigates Drug-Induced Liver Injury *via* Dual Boosting NRF2 and PGAM5 Signaling**

Yanyan Deng^a,1^, Leizhi Xu^a,1^, Xiaoting Niu^a^, Jingjing Li^a^, Yuan Xiong^a^, Guanghao Zhu^a^, Zhiyi Lu^c^, Chuting Xu^a^, Xuerui Wang^a^, Pu Wang^a^, Jian Huang^b^, Zhangping Xiao^a^, Frank J. Gonzalez^d^, Lili Ji^c,**^, Caixia Sun^a,**^, Ping Wang^a,**^, Guangbo Ge^a,*^

^a^ State Key Laboratory of Discovery and Utilization of Functional Components in Traditional Chinese Medicine, Shanghai Frontiers Science Center of TCM Chemical Biology, Institute of Interdisciplinary Integrative Medicine Research, Shanghai University of Traditional Chinese Medicine, Shanghai, 201203, China.

^b^ Pharmacology and Toxicology Division, Shanghai Institute of Food and Drug Control, Shanghai 201203, China.

^c^ The MOE Key Laboratory for Standardization of Chinese Medicines, Shanghai Key Laboratory of Compound Chinese Medicines and The SATCM Key Laboratory for New Resources and Quality Evaluation of Chinese Medicines, Institute of Chinese Materia Medica, Shanghai University of Traditional Chinese Medicine, Shanghai 201203, China.

^d^ Cancer Innovation Laboratory, Center for Cancer Research, National Cancer Institute, National Institutes of Health, Bethesda, MD 20892, USA.

*Corresponding author:

Prof. Guang-Bo Ge, E-mail: [geguangbo@shutcm.edu.cn](mailto:geguangbo@shutcm.edu.cn)

**Co-corresponding authors:

Prof. Ping Wang, E-mail: [pwang@shutcm.edu.cn](mailto:pwang@shutcm.edu.cn)

Prof. Caixia Sun, E-mail: [caixiasun@shutcm.edu.cn](mailto:caixiasun@shutcm.edu.cn)

Prof. Lili Ji, E-mail: [jilili@shutcm.edu.cn](mailto:jilili@shutcm.edu.cn)

^1^ These authors contributed equally to this work.

**This file contains 9 supplementary figures, 1 table and 3 schemes, as well as experiment methods and the details for chemical synthesis and structural characterizations.**


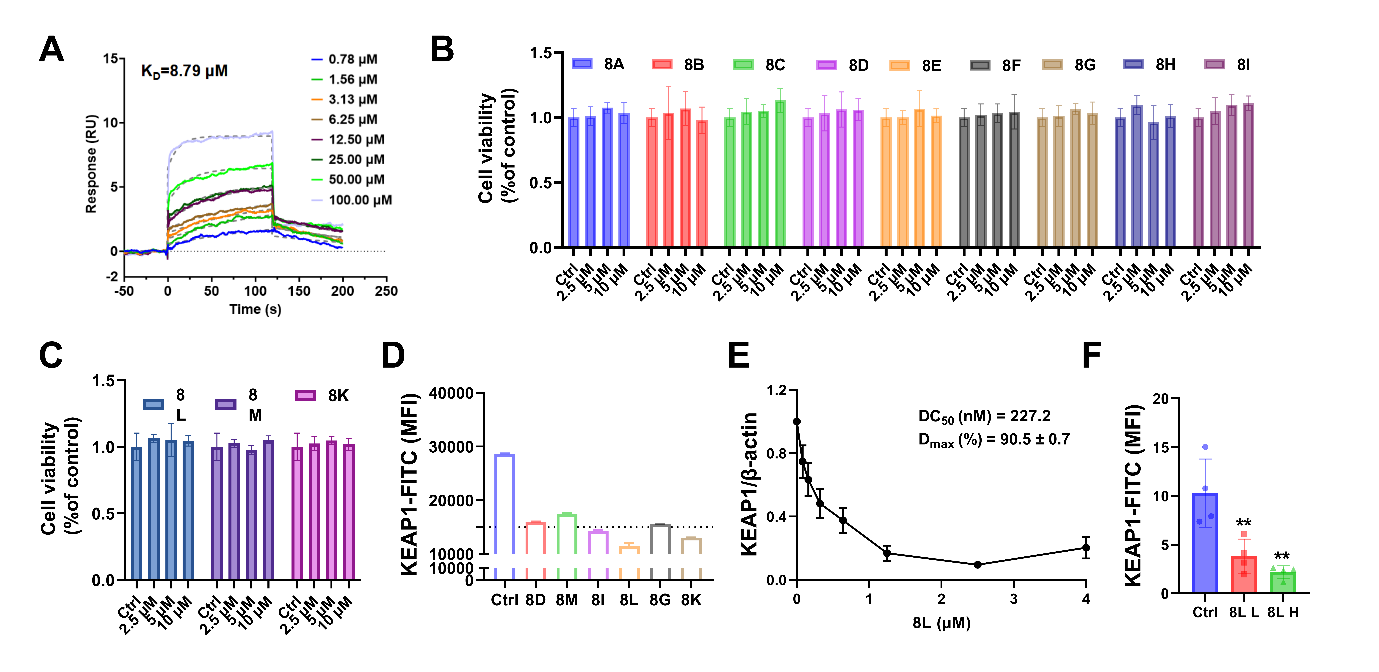


**Figure S1.** (A) Dose-dependent SPR sensorgrams and equilibrium binding responses for **CAD** binding to immobilized KEAP1. **(B)** Cytotoxicity tests of compounds **8A**-**8I** (10 μM, 72 h) in HepaRG cells. **(C)** Cytotoxicity of compounds **8L**-**8M** (10 μM, 72 h) in HepaRG cells. **(D)** The protein levels of KEAP1 were detected by flow cytometry, n=3. **(E)** Western blot (WB) analysis quantified KEAP1 levels in HepaRG cells following **8L** treatment across a concentration range. Densitometry (three experiments) yielded a dose-response curve with DC_50_ and D_max_. **(F)** The mean fluorescence intensity of KEAP1 (Green) in HepaRG 3D spheroids upon addition of **8L**, n=4. Note: Data are presented as mean ± SD, *^*^P*< 0.05, *^**^P*< 0.01 *vs.* control group.

**
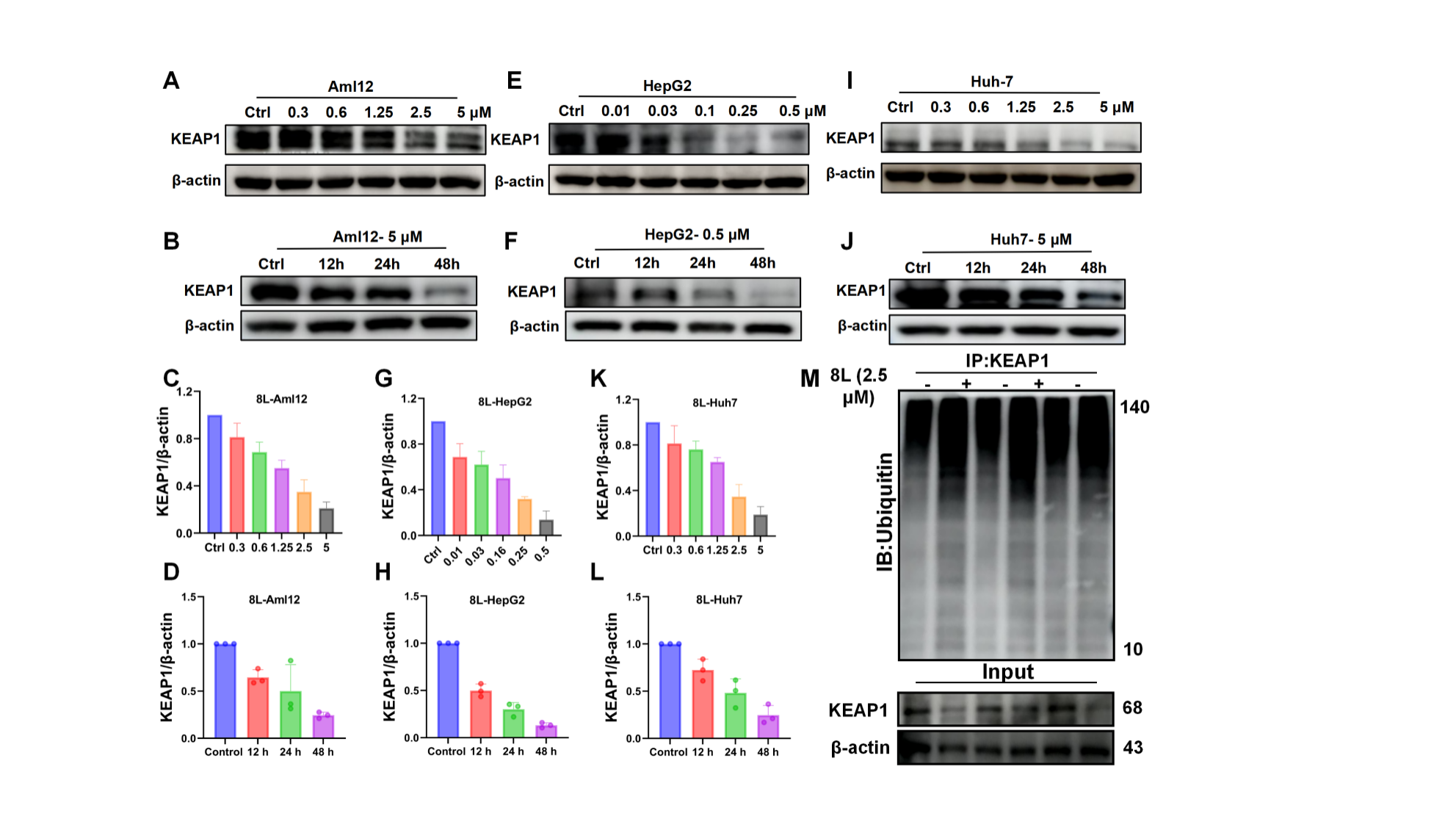
**

**Figure S2. (A** and **C)** The dose-dependent effect on the degradation efficiency of compound **8L** in AML-12 cells and the relative levels of KEAP1. **(B** and **D)** The time-dependent effect on the degradation efficiency of compound **8L** at 5 μM in AML-12 cells and the relative levels of KEAP1. **(E** and **G)** The dose-dependent effect on the degradation efficiency of compound **8L** in HepG2 cells and the relative levels of KEAP1. **(F** and **H)** The time-dependent effect on the degradation efficiency of compound **8L** at 0.5 μM in HepG2 cells. **(I** and **K)** The dose-dependent effect on the degradation efficiency of compound **8L** in Huh-7 cells**. (J** and **L)** The time-dependent effect on the degradation efficiency of compound **8L** at 5 μM in Huh-7 cells. **(M)** The ubiquitization level of KEAP1 treated with or without compound **8L** (5 μM) was detected by Co-IP at 12 h in HepaRG.


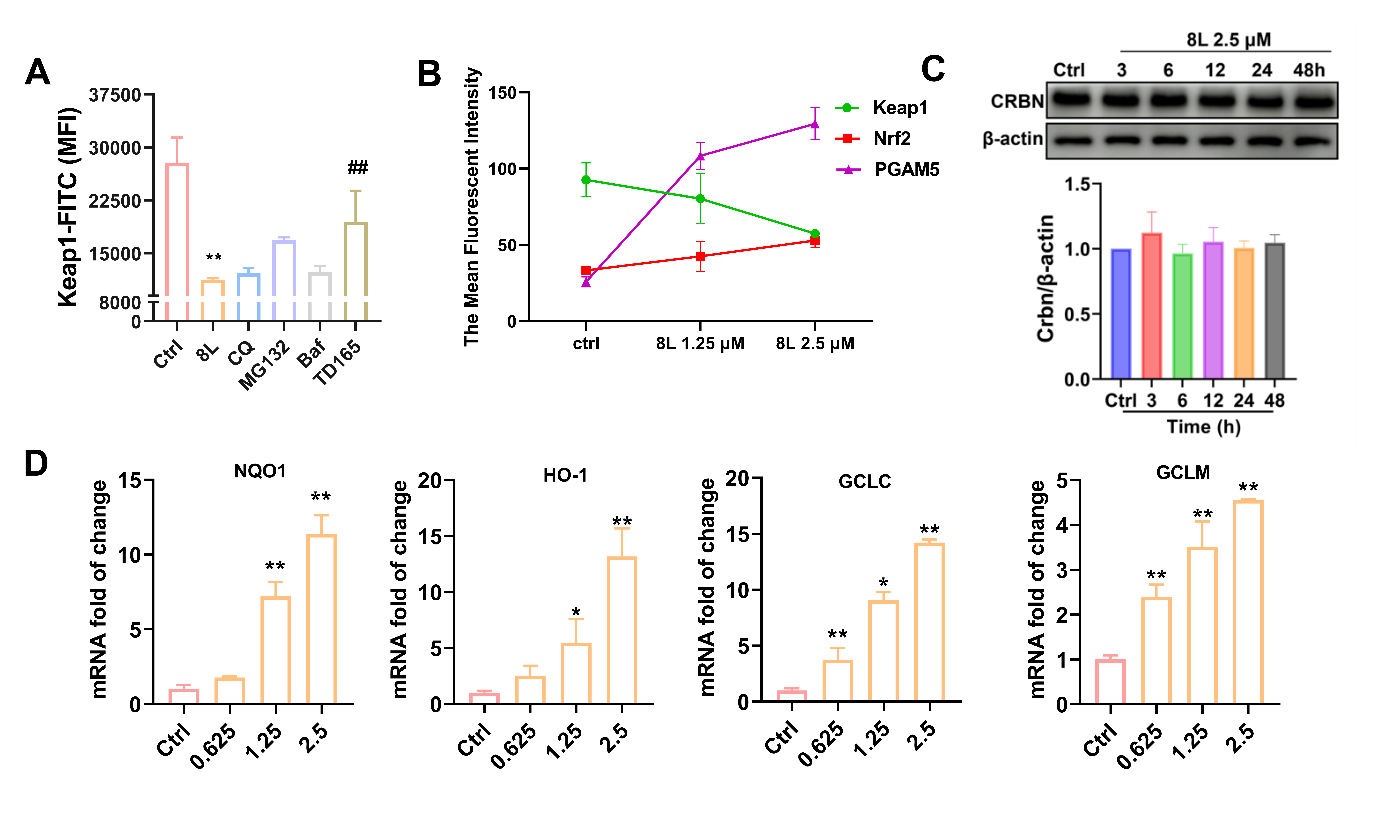


**Figure S3. (A)** The protein levels of KEAP1 were detected by flow cytometry, n = 3. **(B)** The mean fluorescence intensity of KEAP1 (green), NRF2 (red), and PGAM5 (purple) in HepaRG cells treated with different does of **8L**, n = 3. **(C)** The time-dependent extent of CRBN protein by **8L** (2.5 μM) in HepaRG cells, n = 3. **(D)** The mRNA levels of *HO-1*, *NQO1*, *GCLC* and *GCLM* in HepaRG cells were quantified using qRT-PCR, n = 3. Note: Data are presented as mean ± SD, *^*^P*< 0.05, *^**^P*< 0.01 *vs.* control group; *^#^P*< 0.05, *^##^P*< 0.01 *vs.* **8L** group.


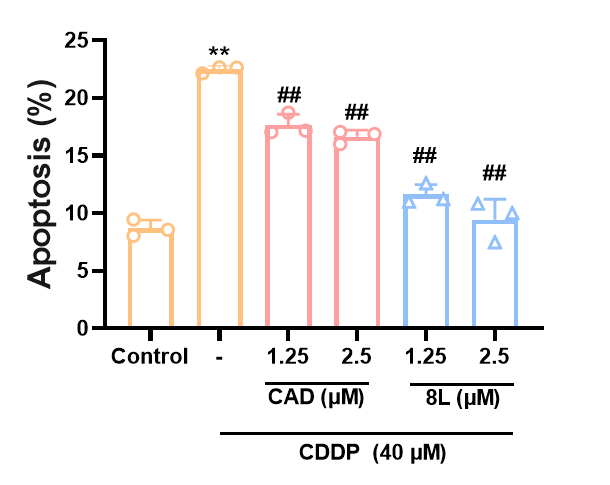


**Figure S4.** The percentage of apoptosis cells in different groups were monitored by flow cytometry. Note: **CDDP** (40 μM), data are presented as mean ± SD, *^*^P*< 0.05, *^**^P*< 0.01 *vs.* control group; *^#^P*< 0.05, *^##^P*< 0.01 *vs.* CDDP group.

**
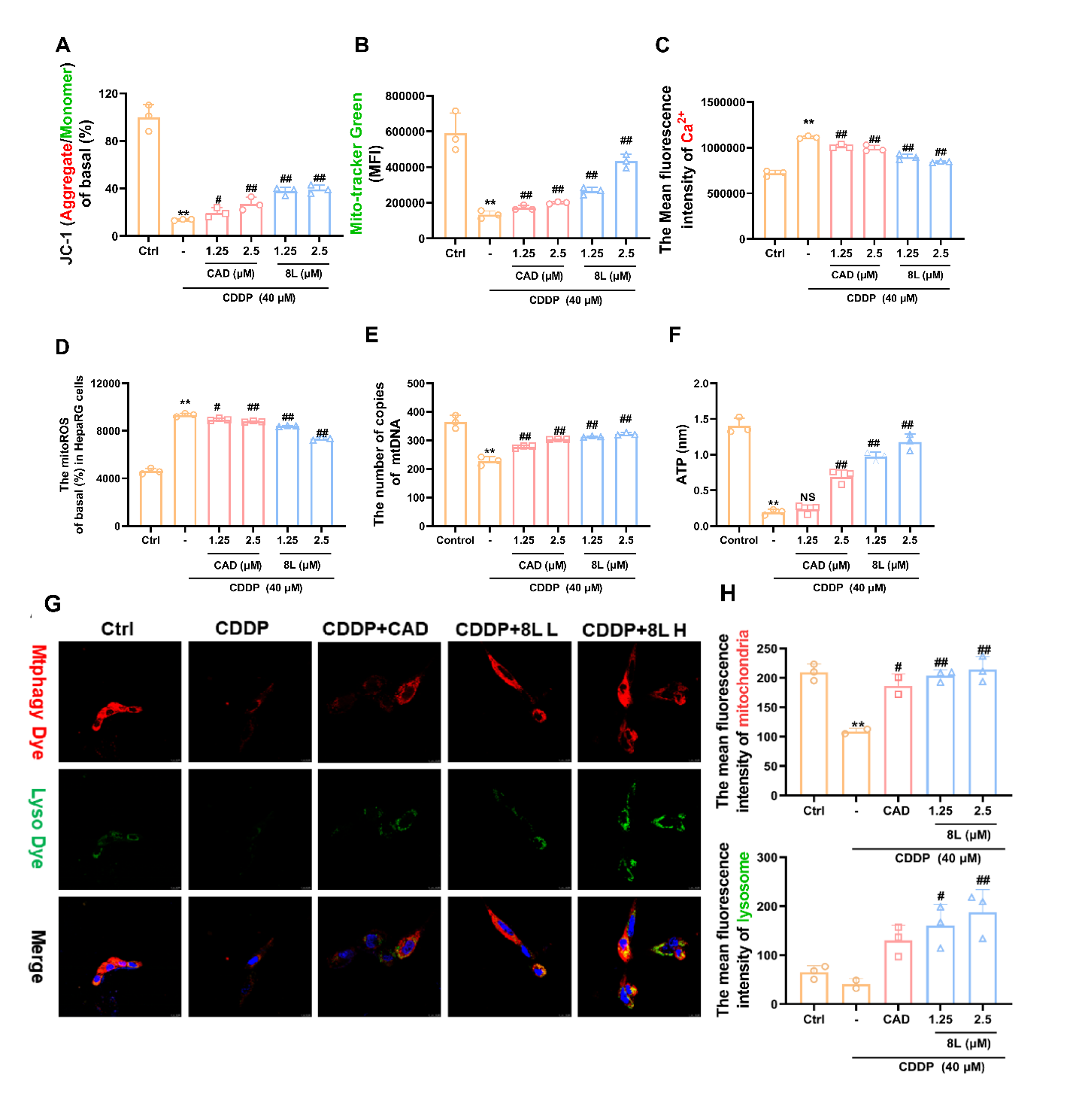
Figure S5.** **(A)** The mitochondrial membrane potentials (ΔΨm) in different groups were monitored by flow cytometry. **(B)** The mitochondrial mass in HepaRG cells were measured by flow cytometry. **(C)** The Ca^2+^ levels among different groups, which were examined by flow cytometry. **(D)** Quantitative analysis of fluorescence intensity of mtROS in HepaRG cells, n = 3. **(E)** The number of copies of mtDNA in HepaRG cells, n = 3. **(F)** Quantification of the relative ATP contents in HepaRG cells, n = 3. **(G)** Mitophagy in HepaRG cells was assessed by fluorescence imaging, where mitophagosomes appeared red and their fusion with lysosomes appeared green, scale bar = 10 μm, n = 3. **(H)** Quantification data of red fluorescence of Mtphagy Dye and green fluorescence of Lsyo Dye. Note: **CDDP** (40 μM), data are presented as mean ± SD, *^*^P*< 0.05, *^**^P*< 0.01 *vs.* control group; *^#^P*< 0.05, *^##^P*< 0.01 *vs.* CDDP group.


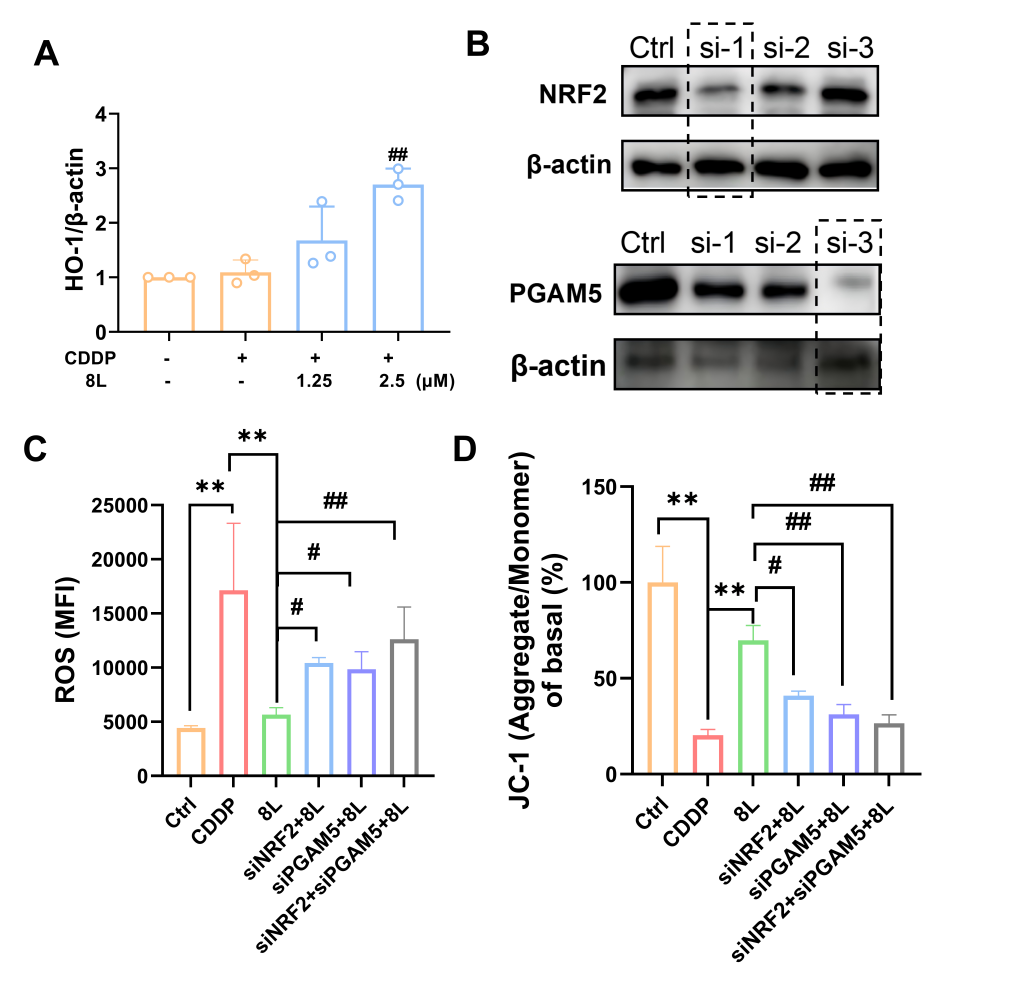


**Figure S6. (A)** The relative protein levels of HO-1 in the cellular model, n = 3, data are presented as mean ± SD, ^##^*P*< 0.01 vs. CDDP group. **(B)** Validation of siRNA-mediated knockdown of NRF2 and PGAM5 in HepaRG cells. **(C)** The levels of ROS in HepaRG cells are measured by flow cytometry, **8L** (2.5 μM), n = 3. **(D)** Mitochondrial membrane potential was measured in different groups *via* JC-1-based flow cytometry, **8L** (2.5 μM), n = 3. Note: **CDDP** (40 μM), data are presented as mean ± SD, ^*^*P*< 0.05, ^**^*P*< 0.01 vs. CDDP group; ^#^*P*< 0.05, ^##^*P*< 0.01 vs. **8L** group.

**
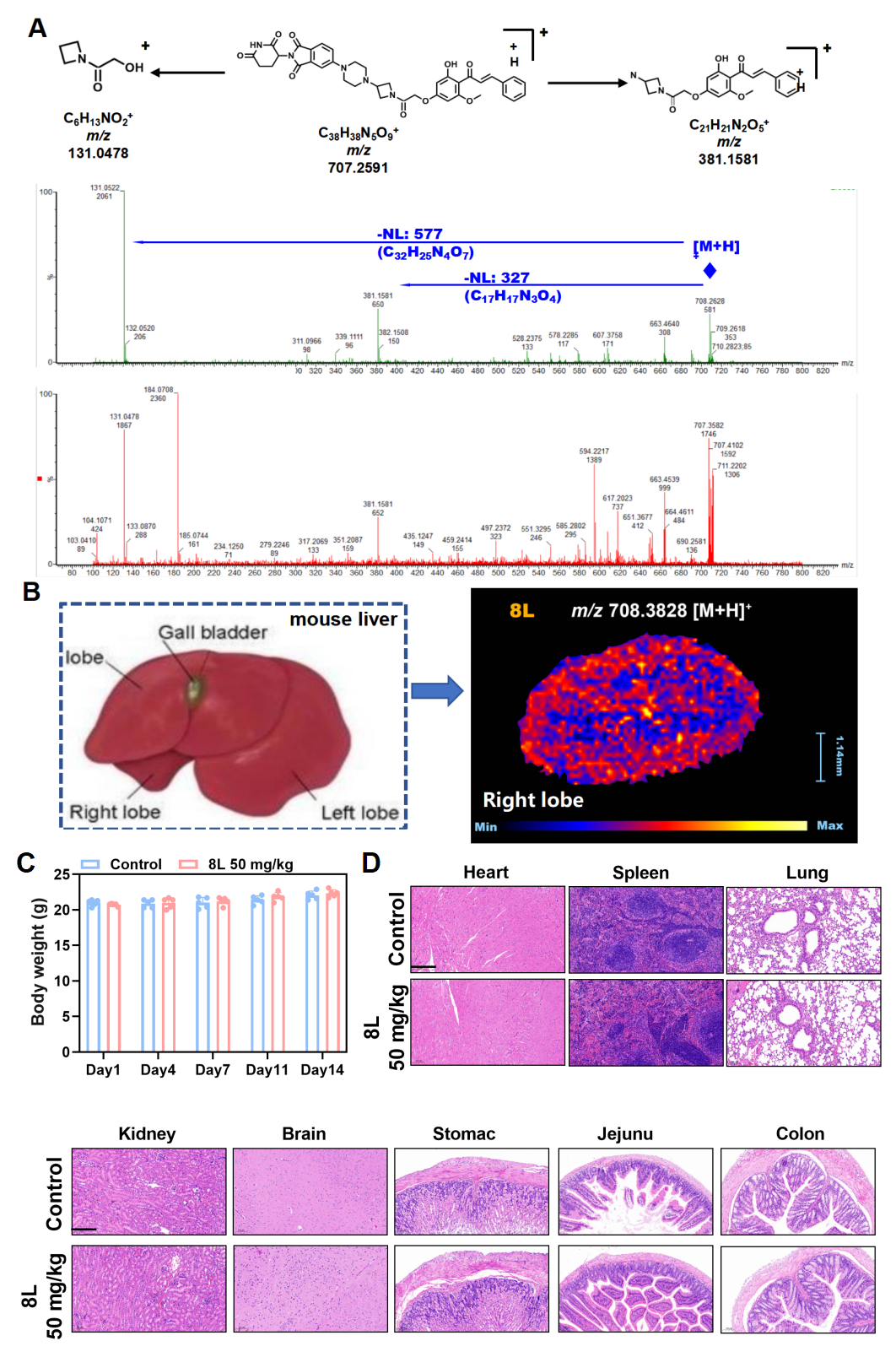
**

**Figure S7. (A)** The MS/MS spectrum of *m/z* 707.2591 acquired from standard reagent with fragment pathway of **8L.** **(B)** The MS/MS spectrum of *m/z* 708.3828 obtained from liver tissue.

**
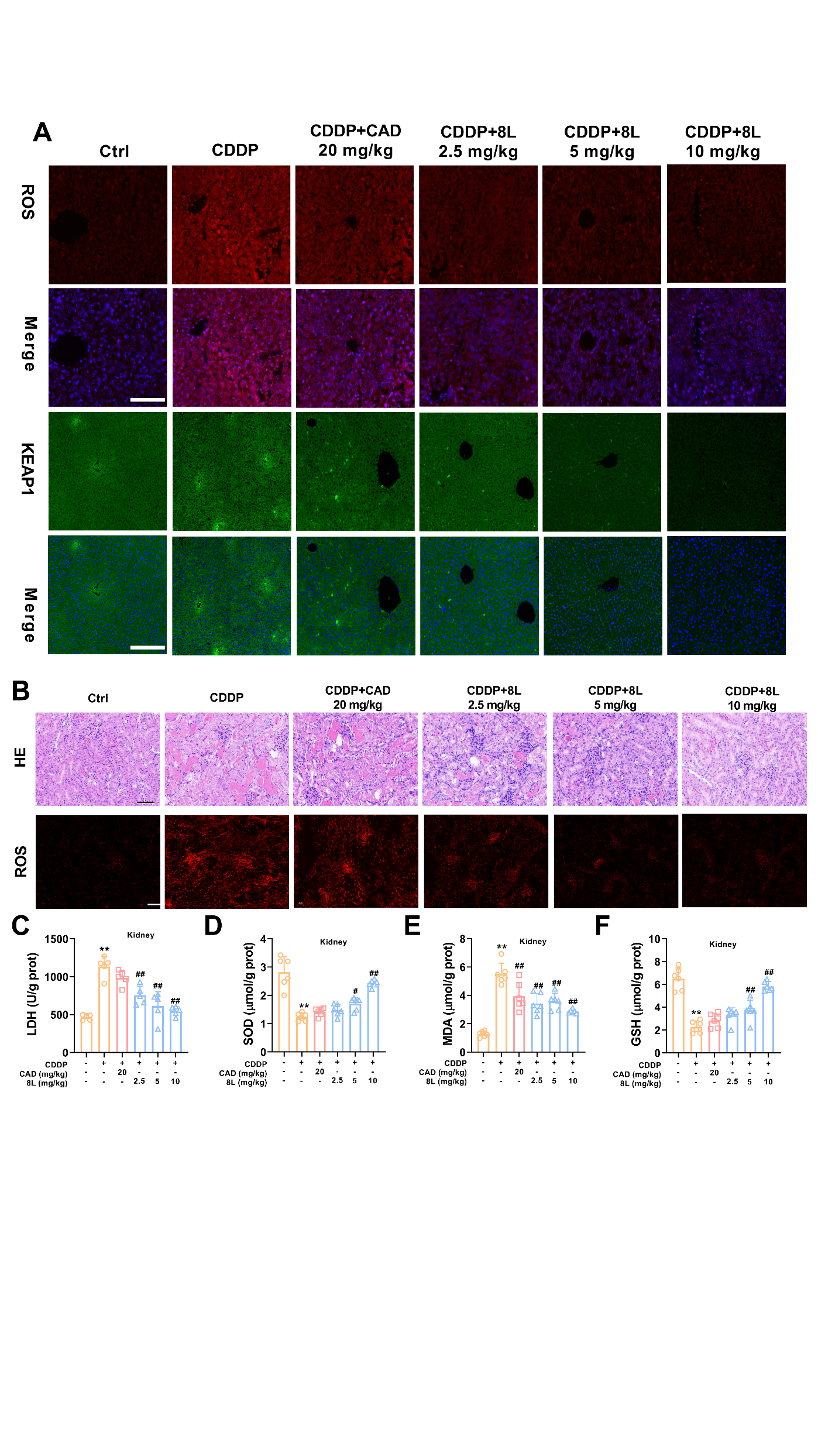
**

**Figure S8.** **8L mitigates CDDP-induced kidney injury. (A)** The levels of intracellular ROS and KEAP1 in liver, and the fluorescence intensity of ROS and KEAP1; Scale bar = 200 μm, n = 5. **(B)** Histopathological changes of the kidney among different groups. Scale bar = 50 μm. **(C-F)** The levels of LDH, GSH, SOD and MDA in kidney tissues collected from different groups. Note: Data are presented as mean ± SD, *^*^P*< 0.05, *^**^P*< 0.01 *vs.* control group; *^#^P*< 0.05, *^##^P*< 0.01 *vs.* CDDP group, n=5.


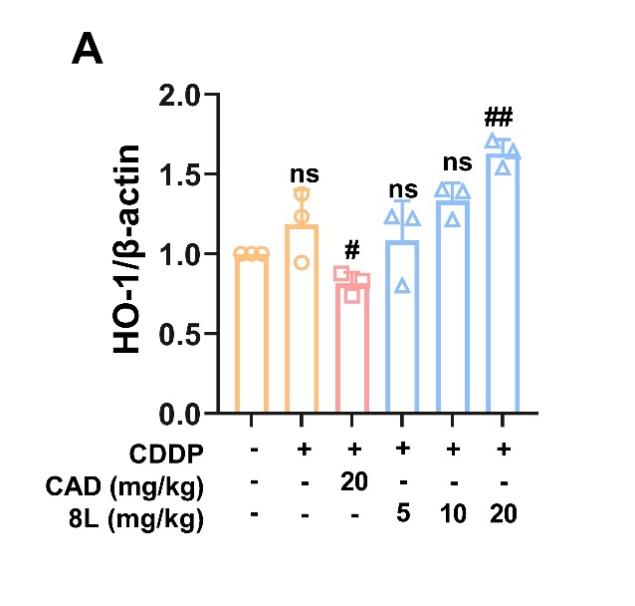


**Figure S9.** The relative protein levels of HO-1 in CDDP-treated liver tissue from different groups, n = 3. Note: Data are presented as mean ± SD, *^*^P*< 0.05, *^**^P*< 0.01 *vs.* control group; *^#^P*< 0.05, *^##^P*< 0.01 *vs.* CDDP group.


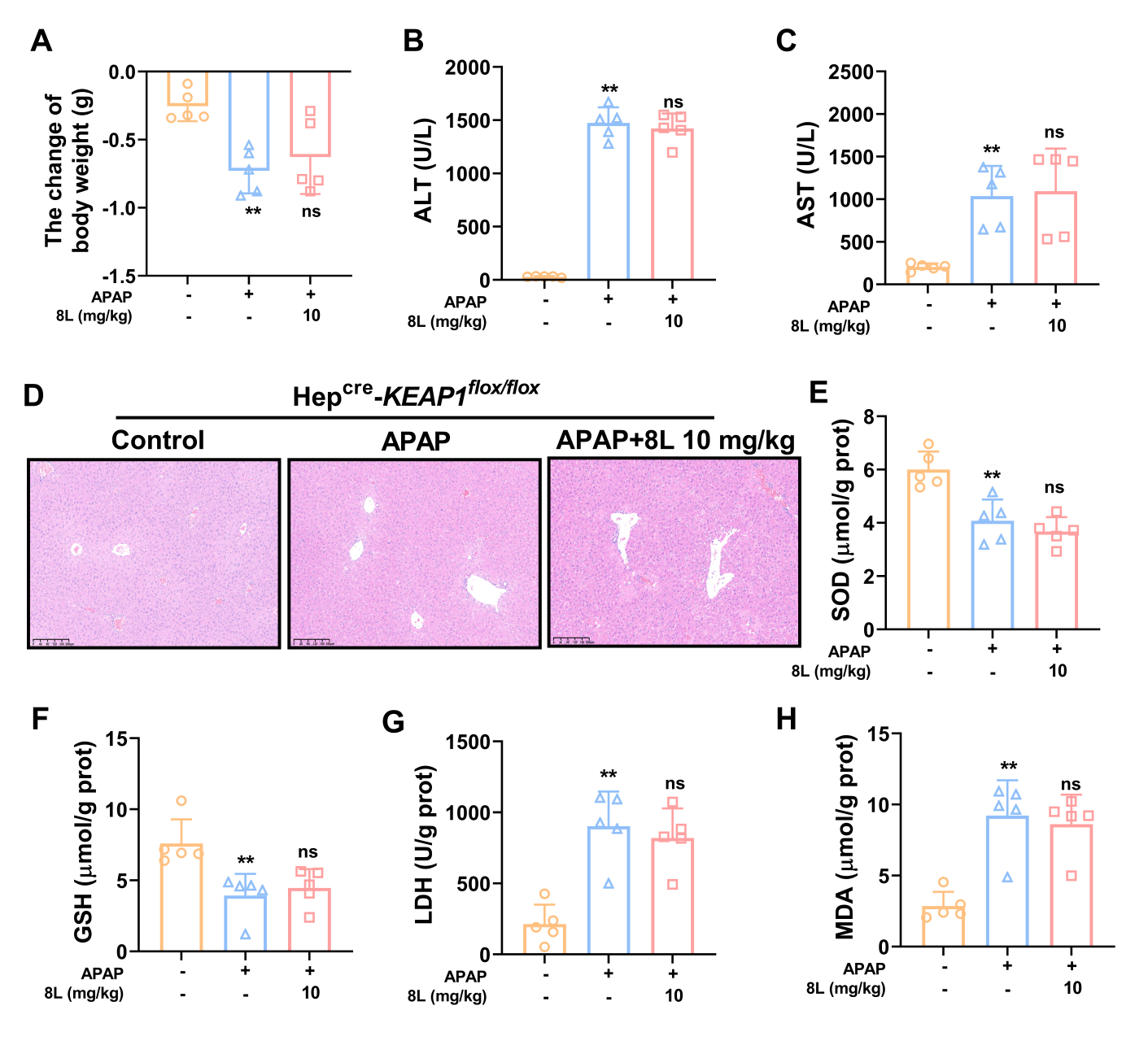


**Figure S10.** **The hepatoprotective effects of 8L on DILI is abolished in Hep^cre^-*KEAP1^flox/flox^* mice. (A)** The changes in body weight of mice from different groups. **(B** and **C)** Serum levels of ALT and AST, among different groups. **(D)** Histopathological changes of the liver among different groups. Scale bar = 200 μm. **(E-H)** The levels of LDH, GSH, SOD and MDA in liver tissues collected from different groups. Note: Data are presented as mean ± SD, *^*^P*< 0.05, *^**^P*< 0.01 *vs.* control group; ns *vs*. APAP group, n = 5.

**Table S1. The NRF2 induction and KEAP1 binding thermal shift potentials of an in-house compound library. Data are presented as mean ± SD (n = 3).**

| **No.** | **Compound** | **CAS** | **^a^Induction potential (fold)** | **^b^ΔTm (℃) *vs* Control** |
| --- | --- | --- | --- | --- |
| 1 | DMSO | -- | 1 | 0 |
| 2 | Sulforaphane (positive control) | 4478-93-7 | 1.71 | 1.55 |
| 3 | **Xanthohumol** | **6754-58-1** | **3.82** | **2.53** |
| 4 | Isorhamnetin | 480-19-3 | 1.01 | 1.75 |
| 5 | Przewaquinone A | 76843-23-7 | 1.04 | 2.53 |
| 6 | Przewaquinone C | 96839-29-1 | 2.69 | 2.14 |
| 7 | Isoastilbin | 54081-48-0 | 0.51 | 1.94 |
| 8 | Dehydromiltirone | 116064-77-8 | 2.06 | 1.75 |
| 9 | Tanshindiol B | 97465-70-8 | 1.29 | 1.36 |
| 10 | Neocryptotanshinone | 109664-02-0 | 1.61 | 1.75 |
| 11 | Cynaroside | 5373-11-5 | 0.74 | 1.94 |
| 12 | 11-hydroxy-sugiol | 88664-08-8 | 1.76 | 1.36 |
| 13 | Ferulic Acid | 1135-24-6 | 2.09 | 1.17 |
| 14 | Methylnissolin | 73340-41-7 | 1.82 | 1.94 |
| 15 | Hematoxylin | 517-28-2 | 1.77 | 1.26 |
| 16 | Butein | 487-52-5 | 1.78 | 1.55 |
| 17 | (3S)-3-Butyl-4,5-dihydro-2-benzofuran-1(3H)-one | 62006-39-7 | 1.78 | 1.36 |
| 18 | Herbacetin 7-rhamnoside | 85571-15-9 | 1.7 | 2.14 |
| 19 | Isoalantolactone | 470-17-7 | 1.5 | 2.04 |
| 20 | (-)-Gallocatechin | 3371-27-5 | 1.47 | 1.75 |
| 21 | p-coumaroylagmatine | 7295-86-5 | 1.86 | 0.85 |
| 22 | Licoisoflavone B | 66056-30-2 | 1.28 | 0.39 |
| 23 | Amentoflavone | 1617-53-4 | 0.93 | 1.56 |
| 24 | Nobiletin | 478-01-3 | 0.93 | 0.39 |
| 25 | Isoflavone | 574-12-9 | 2.52 | 0.97 |
| 26 | Acacetin | 480-44-4 | 2.74 | 1.36 |
| 27 | Oroxylin A | 480-11-5 | 3.4 | 0.39 |
| 28 | Isoliquiritigenin | 961-29-5 | 2.35 | 0.58 |
| 29 | Herbacetin | 527-95-7 | 0.93 | 0.78 |
| 30 | Ginsenoside Rk2 | 364779-14-6 | 1.25 | 2.73 |
| 31 | Brazilin | 474-07-7 | 2.74 | 0.78 |
| 32 | 3',4',7-Trihydroxyisoflavone | 485-63-2 | 3.14 | 0.97 |
| 33 | Calycosin-7-O-β-D-glucoside | 20633-67-4 | 2.16 | 1.17 |
| 34 | 5-Hydroxyflavone | 491-78-1 | 0.96 | 1.94 |
| 35 | Galangin | 548-83-4 | 0.92 | 0.19 |
| 36 | Isovitexin | 29702-25-8 | 1.14 | 1.55 |
| 37 | Morin | 480-16-0 | 1.61 | 0.19 |
| 38 | Cynaroside | 5373-11-5 | 0.99 | 1.36 |
| 39 | Hydroxy safflor yellow A | 78281-02-4 | 1.42 | 2.14 |
| 40 | Genkwanin | 437-64-9 | 1.6 | 1.55 |
| 41 | Carnosol | 5957-80-2 | 1.82 | 0.91 |
| 42 | Glabrone | 60008-02-8 | 1.36 | 0.58 |
| 43 | Hesperidin | 520-26-3 | 1.11 | 1.95 |
| 44 | Naringin | 10236-47-2 | 1.19 | 0.39 |
| 45 | Bavachin | 19879-32-4 | 1.23 | 0.78 |
| 46 | Epimedin A | 110623-72-8 | 1.23 | 0.58 |
| 47 | Epmedin B | 110623-73-9 | 3.33 | 1.95 |
| 48 | Pectolinarigenin | 520-12-7 | 2.87 | 1.17 |
| 49 | Kaempferol | 520-18-3 | 1.29 | 0.39 |
| 50 | Dihydrokaempferol | 480-20-6 | 1.98 | 2.34 |
| 51 | Irisflorentin | 41743-73-1 | 1.43 | 0.78 |
| 52 | Hyperoside | 482-36-0 | 1.04 | 0.19 |
| 53 | Scutellarin | 27740-01-8 | 0.93 | 0.58 |
| 54 | Scutellarein | 529-53-3 | 1.13 | 1.55 |
| 55 | Baicalin | 21967-41-9 | 1.17 | 1.36 |
| 56 | Baicalein | 491-67-8 | 0.99 | 1.55 |
| 57 | Sinensetin | 2306-27-6 | 0.97 | 2.14 |
| 58 | Glycitin | 40246-10-4 | 1.33 | 0.58 |
| 59 | Wogonoside | 51059-44-0 | 1.02 | 1.36 |
| 60 | Isoginkgetin | 548-19-6 | 0.86 | 0.97 |
| 61 | Ampelopsin | 27200-12-0 | 1.47 | 0.98 |
| 62 | Kaempferol 7-O-β-D-glucopyranoside | 16290-07-6 | 0.89 | 0.58 |
| 63 | Vanillic acid | 121-34-6 | 0.91 | -0.19 |
| 64 | 1,2-Benzenedicarboxylic acid | 84-74-2 | 0.8 | 0.97 |
| 65 | Palmitic acid | 57-10-3 | 0.86 | 0.70 |
| 66 | Chrysophanic acid | 481-74-3 | 0.93 | 1.17 |
| 67 | Aspirin | 50-78-2 | 1.23 | 0.97 |
| 68 | Quercitrin | 522-12-3 | 0.87 | 2.14 |
| 69 | Chrysoeriol | 491-71-4 | 0.25 | 0.58 |
| 70 | Shikonine | 517-89-5 | 0.42 | 0.39 |
| 71 | Cilostazol | 73963-72-1 | 1.66 | 0.19 |
| 72 | Rosmarinic acid | 20283-92-5 | 3.98 | 2.53 |
| 73 | Echinatin | 34221-41-5 | 3.81 | 0.78 |
| 74 | Apigenin | 485-72-3 | 2.16 | 0.97 |
| 75 | Eriodictyol | 552-58-9 | 1.31 | 1.55 |
| 76 | Homoeriodictyol | 69097-98-9 | 1.05 | 0.97 |
| 77 | Magnoflorine | 2141-09-5 | 1.14 | 1.75 |
| 78 | Luteolin 7-diglucuronide | 96400-45-2 | 1.18 | 1.17 |
| 79 | Robinin | 301-19-9 | 1.14 | 0.58 |
| 80 | Tamarixetin | 603-61-2 | 1.08 | 1.17 |
| 81 | Tamarixetin 7-β-D-glucopyranoside | 16290-09-8 | 2.81 | 0.83 |
| 82 | Glabridin | 59870-68-7 | 1.01 | 1.36 |
| 83 | Bergaptol | 486-60-2 | 1.26 | 0.97 |
| 84 | Cirsimarin | 13020-19-4 | 1.24 | 2.34 |
| 85 | Chrysin 7-O-neohesperidoside | 35775-46-3 | 1.41 | -0.39 |
| 86 | Ombuoside | 20188-85-6 | 1.19 | 0.19 |
| 87 | Isorhamnetin-3-O-beta-D-Glucoside | 5041-82-7 | 3.58 | 1.17 |
| 88 | 6-Methoxykaempferol | 32520-55-1 | 0.94 | 0.58 |
| 89 | Quercetin 3-O-sambubioside | 83048-35-5 | 0.88 | 1.17 |
| 90 | Fortunellin | 20633-93-6 | 0.98 | 2.14 |
| 91 | Clerodendrin | 119738-57-7 | 0.98 | 1.75 |
| 92 | Prunin | 529-55-5 | 1.06 | 0.97 |
| 93 | Notoginsenoside Ft1 | 155683-00-4 | 0.99 | 1.75 |
| 94 | Ginsenoside Rg3 | 14197-60-5 | 1.16 | 0.39 |
| 95 | 20(R)-Ginsenoside Rg3 | 38243-03-7 | 1.02 | 2.33 |
| 96 | Ginsenoside Rk1 | 494753-69-4 | 0.87 | 0.39 |
| 97 | Ginsenoside Rg5 | 186763-78-0 | 0.79 | -0.19 |
| 98 | 20(S)-Ginsenoside Rh2 | 7821-33-2 | 0.91 | 2.14 |
| 99 | 20(R)-Ginsenoside Rh2 | 112246-15-8 | 0.93 | 0.78 |
| 100 | Chlorogenic acid | 327-97-9 | 0.84 | 2.33 |
| 101 | Ginsenoside Rh4 | 174721-08-5 | 1.09 | 0.80 |
| 102 | Glycyrrhizic acid | 1405-86-3 | 1.03 | -0.58 |
| 103 | Gypenoside XVII | 80321-69-3 | 1.16 | 0.78 |
| 104 | Notoginsenoside Fe | 88105-29-7 | 1.14 | 0.58 |
| 105 | Ginsenoside Rd2 | 83480-64-2 | 1.12 | 0.78 |
| 106 | Notoginsenoside Fc | 88122-52-5 | 0.97 | 0.19 |
| 107 | Ginsenoside F2 | 62025-49-4 | 1.02 | 0.58 |
| 108 | Notoginsenoside R1 | 80418-24-2 | 1.06 | 1.17 |
| 109 | Ginsenoside Rg1 | 22427-39-0 | 0.91 | 1.56 |
| 110 | Coniferyl ferulate | 63644-62-2 | 1.78 | 1.17 |
| 111 | Ginsenoside Re | 52286-59-6 | 1.38 | 0.97 |
| 112 | Senkyunolide | 63038-10-8 | 1.46 | 1.75 |
| 113 | Butylbenzene | 104-51-8 | 1.44 | 0.39 |
| 114 | Ligustilide | 4431-01-0 | 1.03 | 0.00 |
| 115 | Senkyunolide G | 94530-85-5 | 1.05 | 1.36 |
| 116 | Geniposide | 24512-63-8 | 1.91 | -0.19 |
| 117 | Geniposidic acid | 27741-01-1 | 2.38 | 0.78 |
| 118 | Licoflavone C | 72357-31-4 | 1.06 | 1.55 |
| 119 | Semilicoisoflavone B | 129280-33-7 | 1 | 0.58 |
| 120 | glycycoumarin | 94805-82-0 | 0.39 | 0.00 |
| 121 | Licoricone | 51847-92-8 | 1.18 | 0.93 |
| 122 | Licoisoflavone A | 66056-19-7 | 1.45 | 0.78 |
| 123 | Glycyrrhetinic acid | 471-53-4 | 1.44 | 2.92 |
| 124 | Coniferyl ferulate | 63644-62-2 | 1.4 | 0.97 |
| 125 | -)-Epigallocatechin (EGC) | 970-74-1 | 0.13 | 0.39 |
| 126 | Oridonin | 28957-04-2 | 1.25 | 0.78 |
| 127 | Anhydroicaritin | 38226-86-7 | 1.2 | 0.78 |
| 128 | 3,4-Dihydroxybenzaldehyde | 139-85-5 | 1.25 | 0.58 |
| 129 | Senkyunolide H | 94596-27-7 | 1.2 | 1.75 |
| 130 | Senkyunolide E | 94530-83-3 | 1.1 | 2.53 |
| 131 | Carnosol | 5957-80-2 | 1.02 | 0.97 |
| 132 | Gallic acid | 149-91-7 | 0.98 | 0.58 |
| 133 | Cinnamic acid | 140-10-3 | 0.86 | 2.34 |
| 134 | Z-Ligustilide | 81944-09-4 | 0.8 | -0.58 |
| 135 | Lithocholic acid | 434-13-9 | 2.51 | 1.75 |
| 136 | Proanthocyanidins | 20347-71-1 | 0.54 | 0.19 |
| 137 | Tanshinone IIB | 17397-93-2 | 0.4 | 1.55 |
| 138 | Tanshinone I | 568-73-0 | 0.38 | 2.33 |
| 139 | Cilostazol | 73963-72-1 | 0.3 | 1.17 |
| 140 | Cryptotanshinone | 35825-57-1 | 2.35 | 1.36 |
| 141 | Shikonine | 517-89-5 | 3.14 | 0.90 |
| 142 | Diosmetin | 520-34-3 | 1.01 | 0.78 |
| 143 | Ethyl 3-(3,4-dihydroxyphenyl) acrylate | 102-37-4 | 0.77 | 0.58 |
| 144 | 3,3',4',5,6,7-Hexahydroxyflavone | 90-18-6 | 1.03 | 0.97 |
| 145 | Tanshinone IIA sodium sulfonate | 69659-80-9 | 1.46 | 0.78 |
| 146 | Isosilybin A | 142796-21-2 | 2.59 | 1.36 |
| 147 | Eriocitrin | 13463-28-0 | 2.5 | 1.75 |
| 148 | Methyl caffeate acid | 3843-74-1 | 2.4 | 1.17 |
| 149 | **Miltirone** | **82209-72-1** | **5.4** | **3.12** |
| 150 | **Hispidulin** | **546-43-0** | **5.65** | **3.51** |
| 151 | **Formononetin** | **520-36-5** | **5.63** | **3.31** |
| 152 | Andropanoside | 27210-57-7 | 2.3 | 3.00 |
| 153 | Alantolactone | 1447-88-7 | 3.82 | 2.34 |
| 154 | Caffeic acid | 501-16-6 | 1.15 | 1.36 |
| 155 | 15,16-Dihydrotanshindiol C | 19309-14-9 | 0.56 | 0.39 |
| 156 | **Cardamonin** | **891854-96-9** | **6.22** | **3.70** |
| 157 | Deoxyneocryptotanshinone | 27468-20-8 | 1.38 | 1.94 |
| 158 | Liquiritigenin | 578-86-9 | 0.95 | 0.78 |
| 159 | Oxypeucedanin | 737-52-0 | 1.48 | 1.75 |
| 160 | Homoeriodictyol | 69097-98-9 | 1.18 | 1.55 |
| 161 | Farrerol | 24211-30-1 | 1.31 | 1.22 |
| 162 | Silymarin | 22888-70-6 | 0.85 | 2.53 |
| 163 | Nebadensin | 152743-19-6 | 1.07 | 1.17 |
| 164 | Neoliquiritin | 5188-75-5 | 1.29 | 0.39 |
| 165 | Quercetin | 117-39-5 | 1.68 | 1.17 |
| 166 | Isosinensetin | 17290-70-9 | 3.12 | 2.33 |
| 167 | Rosmanol | 80225-53-2 | 0.71 | 1.55 |
| 168 | Neoisoliquiritin | 59122-93-9 | 0.9 | 1.75 |
| 169 | Scutellarein 7-O-diglucuronide | 547743-34-0 | 2.08 | 0.78 |
| 170 | 3'-Hydroxy Puerarin | 117060-54-5 | 1.14 | 1.17 |
| 171 | Levistilide A | 88182-33-6 | 1.27 | -0.19 |
| 172 | Angelicide | 92935-94-9 | 1.54 | 1.75 |
| 173 | 3-n-Butylphthalide | 6066-49-5 | 1.23 | 0.97 |
| 174 | Senkyunolide I | 94596-28-8 | 1.16 | 1.36 |
| 175 | Epicatechin | 490-46-0 | 1.19 | 0.97 |
| 176 | 8,8''-Bibaicalein | 135309-02-3 | 1.2 | 1.75 |
| 177 | Protocatechuic acid | 99-50-3 | 0.93 | 0.00 |
| 178 | Cirsimarin | 13020-19-4 | 1.06 | 1.75 |
| 179 | Neochlorogenic acid | 906-33-2 | 1.25 | 2.53 |
| 180 | Licoflavonol | 60197-60-6 | 0.83 | 1.55 |
| 181 | Narcissoside | 604-80-8 | 2.2 | 1.14 |
| 182 | Rhoifolin | 17306-46-6 | 1.24 | 0.00 |
| 183 | Isorhoifolin | 552-57-8 | 1.09 | 0.19 |
| 184 | Sanchinoside Rh1 | 63223-86-9 | 1.19 | 0.39 |
| 185 | Caffeic acid phenethyl ester | 104594-70-9 | 1.24 | 0.97 |
| 186 | Nicotiflorin | 17650-84-9 | 2.6 | 1.75 |
| 187 | Petanin chloride | 69915-09-9 | 1.28 | 1.17 |
| 188 | Tetramethylkaempferol | 16692-52-7 | 1.47 | 0.97 |
| 189 | Nicotiflorin | 17650-84-9 | 1.76 | -1.17 |
| 190 | Narirutin | 14259-46-2 | 1.48 | 0.58 |
| 191 | Genkwanin | 437-64-9 | 1 | 0.19 |
| 192 | Tilianin | 4291-60-5 | 1.42 | 0.97 |
| 193 | Scrophulein | 6601-62-3 | 1.26 | 0.78 |
| 194 | Axillarin | 5188-73-8 | 3.54 | 1.75 |
| 195 | Eriodictyol-7-O-glucoside | 38965-51-4 | 1.28 | 1.17 |
| 196 | Neoeriocitrin | 13241-32-2 | 1.27 | 0.58 |
| 197 | Eriocitrin | 13463-28-0 | 1.39 | 0.78 |
| 198 | Lonicerin | 25694-72-8 | 1.46 | 1.94 |
| 199 | Vanillin | 121-33-5 | 1.17 | 0.39 |
| 200 | Licoricone | 51847-92-8 | 1.23 | 1.36 |
| 201 | Xanthotoxin | 298-81-7 | 1 | 1.24 |

^a^treated with 10 μM natural compounds for 24 h, ^b^treated with 100 μM natural compounds for 1 h

**Supplementary experimental and methods**

*Transmission Electron Microscopy (TEM) Sample*

Liver tissues (approximately 1 mm³) were fixed in 2.5% glutaraldehyde solution at 4 °C for 24 h. The fixative was then removed, and the samples were rinsed three times with 0.1 M PBS (pH 7.4) for 15 min each. Subsequently, the samples were post‑fixed with 1% osmium tetroxide solution for 1-2 h. After careful removal of the osmium solution, the samples were washed three times with phosphate buffer (15 min each). Dehydration was carried out using a graded ethanol series (30%, 50%, 70%, 80%, 90%, and 95%) for 10 min per step, followed by treatment with absolute ethanol for 20 min. Finally, the samples were incubated in acetone for 20 min. For embedding, the samples were first infiltrated with a mixture of embedding resin and acetone (1:1, v/v) for 1 h, then with a mixture of resin and acetone (3:1, v/v) for 3 h, followed by pure embedding resin overnight. After complete infiltration, the samples were embedded and polymerized at 60 °C overnight. Ultrathin sections (70-90 nm) were cut using an ultramicrotome (LEICA UC7, Germany). The sections were stained with uranyl acetate (saturated in 50% ethanol) for 8-15 min and with lead citrate for 8-10 min, respectively, and then examined under a transmission electron microscope (Hitachi H‑7650).

*Mitochondrial DNA copy number assay*

Genomic DNA was extracted from HepaRG cells using the QIAamp® DNA Trace Kit (QIAGEN, Germany). Mitochondrial DNA copy number was quantified by qRT-PCR with SYBR® Premix Ex Taq™ II (Takara), and the ratio of mitochondrial ND1 to nuclear GAPDH was determined using the 2^⁻ΔΔCt^ method.

*Histological staining methods for the liver*

Liver tissues were fixed in 4% paraformaldehyde, embedded in paraffin wax, and stained in 4 μm sections. H&E staining was performed by deparaffinisation, haematoxylin (5 minutes)/eosin (2 minutes) staining, dehydration and mounting, in order to assess general histopathology.

*Western Blotting*

Proteins from HepaRG cells and liver tissues were extracted using RIPA buffer supplemented with protease and phosphatase inhibitors. RIPA buffer and PAGE quick preparation kits (GF1820) were obtained from Genefist Life Science (Shanghai, China). Total protein (20-40 μg) was separated by SDS-PAGE and transferred to PVDF membranes. Membranes were blocked with 5% non-fat milk and probed with primary antibodies (anti-NRF2, 1:1000; anti-KEAP1, 1:1000; anti-HO-1, 1:1000; anti-PGAM5, 1:1000; anti-β-actin, 1:5000) overnight at 4°C, followed by incubation with HRP-conjugated secondary antibodies (1:5000, Cell Signaling Technology) for 1 h at room temperature. Protein bands were visualized using ECL substrate.

*Cell Experiments*

The HepaRG cell line was passaged and maintained in William’s E (Gibco) supplemented with 10% fetal calf serum (Gibco), 100 µg/mL Streptomycin (Gibco), 100 U/mL Penicillin (Gibco), 2 mM GlutaMax (Gibco), 5 µg/mL insulin (Sigma-Aldrich, Taufkirchen, Germany), and 50 µM hydrocortisone hemisuccinate (Sigma-Aldrich). HepG2 and Huh7 cells (ATCC) were cultured in DMEM medium supplemented with 10% FBS at 37°C under 5% CO_2_. Aml12 cells (ATCC) were cultured in DMEM/F12 medium supplemented with 10% FBS at 37°C under 5% CO_2_.

*Oxidative stress biomarker quantification*

Cells and liver tissue homogenates were centrifuged (10,000 × g, 15 min, 4°C), and supernatants were analyzed for MDA, reduced GSH, and SOD levels using commercial assay kits.

*NRF2 luciferase reporter assay*

293T-NRF2-Luc reporter cells (1×10⁴ cells/well) were treated with 10 μM natural compounds for 24 h. Cells were then lysed, and luciferase activity was measured using Steady-Glo® Reagent (Beyotime Biotechnology). Luciferase activity was quantified *via* Spectramax M4 (Molecular devices, USA).

*Protein Thermal Shift Assay (PTS) for KEAP1*

We established a protein thermal shift assay in 96-well full-skirted white PCR plates (Monad, MQ50701S). The reaction system with a total volume of 10 μL contained 0.5 μL of either DMSO or DMSO-dissolved compounds (final DMSO concentration 5% *v/v*) to achieve a final compound concentration of 100 μM, 5 μM SMUO-KEAP1 protein (final concentration), and 10× SYPRO Orange dye (Sigma, S5692). The plates were centrifuged at 1000 rpm for 30 seconds followed by incubation at room temperature with orbital shaking for 1 minute. The plates were then allowed to incubate at room temperature for an additional 1 hour to ensure sufficient compound-protein interaction. Fluorescence signals were monitored and recorded during temperature ramping from 25°C to 95°C using a Quant Studio 5 real-time PCR system. The thermal denaturation temperature (Tm) values were determined by curve fitting analysis using Protein Thermal Shift Software v1.4.

*Quantitative real-time PCR (RT-qPCR)*

Total RNA was reverse-transcribed into complementary DNA (cDNA) using a commercial reverse transcription kit. Quantitative real-time PCR (RT-qPCR) analysis was subsequently performed on a fluorescence-based detection platform with SYBR Green Master Mix (Roche, USA). Gene expression levels of *HO-1*, *NQO1*, *GCLC*, and*GCLM* were quantified relative to the endogenous reference gene *GAPDH*, with data analysis conducted *via* the comparative threshold cycle (2^−△△Ct^) method. Sequence-specific primer pairs used for amplification are provided in Supplementary Table 2.

*Proteomics*

HepaRG cells were treated with DMSO or 1 μM of **8L** for 48 h. Then collect cells, take 100 μg of the sample for enzymatic digestion. Add 8 M GdmCl (guanidine hydrochloride) to achieve a final concentration of 6 M GdmCl for protein denaturation. Add TCEP to a final concentration of 10 mM and CAA to a final concentration of 40 mM, then heat to 95°C for 5 minutes, followed by cooling on ice for 15 minutes to complete the reduction and alkylation process. Transfer the protein sample to a 10 kDa, 500 μL ultrafiltration tube and centrifuge at 25°C, 14,000 g until the ultrafiltration membrane at the bottom of the tube is just exposed. Add 100 μL of 100 mM ammonium bicarbonate solution and centrifuge at 25°C, 14,000 g until the ultrafiltration membrane at the bottom of the tube is just exposed again; repeat this step twice. Subsequently, replace the collection tube and add 80 μL of 100 mM ammonium bicarbonate solution to the ultrafiltration tube. Add Trypsin (Promega) at a mass ratio of 1:25 (Trypsin:protein) and incubate overnight at 37°C for digestion. After digestion, centrifuge at 25°C, 14,000 g for 20 minutes to collect the flow-through. Wash twice by adding 50 μL of 10 mM ammonium bicarbonate solution and centrifuging at 14,000 g for 20 minutes each time. Combine the collected flow-through solutions, lyophilize, and resuspend the sample in 0.1 % TFA. Desalt using HLB cartridges, lyophilize, and load onto the mass spectrometer (480 mass spectrometer) for analysis.

Unless otherwise indicated, the DIA samples were analyzed on an Orbitrap Exploris 480 coupled with a *nano*-flow LC system (Ultimate 3000 RSLC microsystem, Dionex). Next, 1 μg peptides were separated on a commercial 15 cm × 1 mm i.d. column (ACQUITY UPLC Peptide CSH C18 Column, 130 Å, 1.7 μm; Waters). Binary buffers (A, 0.1 % FA; B, 80 % ACN and 0.1 % FA) were used. Peptides were separated by linear gradients from 6 % B to 32 % B for 80 min followed by a linear increase to 45 % B in 17 min at the flow of 50 μL min^-1^. FAIMS (high-field asymmetric waveform ion mobility spectrometry) parameters were set as follows: compensation voltage, -45 V and total carrier gas flow, 4 L min^-1^. Full MS scans were acquired at 120,000 resolution (*m/z* = 200) spanning from *m/z* 350 to 1,400 with the automatic gain control (AGC) target set to 3 × 10^6^ and a maximum injection time of 45 ms. MS/MS scans were acquired in a DIA mode with a resolution of 30,000 (*m/z* = 200). A total of 24 DIA segments were acquired ranging from *m/z* 400 to 1,000 at a resolution of 30,000 (AGC target of 2 × 10^6^ and auto maximum injection time) with the normalized collision energy of 30 %. The first mass was fixed at *m/z* of 300.

DIA spectra were analyzed with DIA-NN (1.9.1). The spectral library was generated by deep learning-based spectra, RTs and IMs prediction in the DIA-NN algorithm using the reviewed Human UniProt Database containing 20421 proteins (down loaded in 2025). Trypsin was specified as the digestion enzyme, allowing up to two missed cleavages. The mass accuracy for both precursor and MS1 was 10 ppm, with a precursor FDR threshold of 1 %. Default settings were used for all other parameters. When the analyses were finished, the quantitative analysis was performed based on the intensity values provided in the report.pg_matrix file.

*Assessment of siRNA Knockdown*

siRNA (provided by GenePharma) were used for gene function analysis in HepaRG cells. The cells were cultured in William’s E medium supplemented with 10% fetal bovine serum (FBS), insulin, and hydrocortisone. Transfection was performed when cells reached 60-70% confluency. Using Lipofectamine™ 3000 (Thermo Fisher Scientific) as the transfection reagent, 50 pmol of siRNA and 1.5 μL of Lipo3000 were separately diluted in Opti‑MEM, combined, and incubated at room temperature for 15 min to allow complex formation. The original culture medium was then removed, and fresh medium was added to the cells. The siRNA‑Lipo3000 complexes were added dropwise to the culture plates with gentle mixing. Transfection was carried out at 37 °C under 5% CO₂ for 6 h, after which the medium was replaced with complete culture medium. Cells were harvested 48 h post‑transfection, and knockdown efficiency was assessed by WB.

**Supplementary Scheme S1-3.
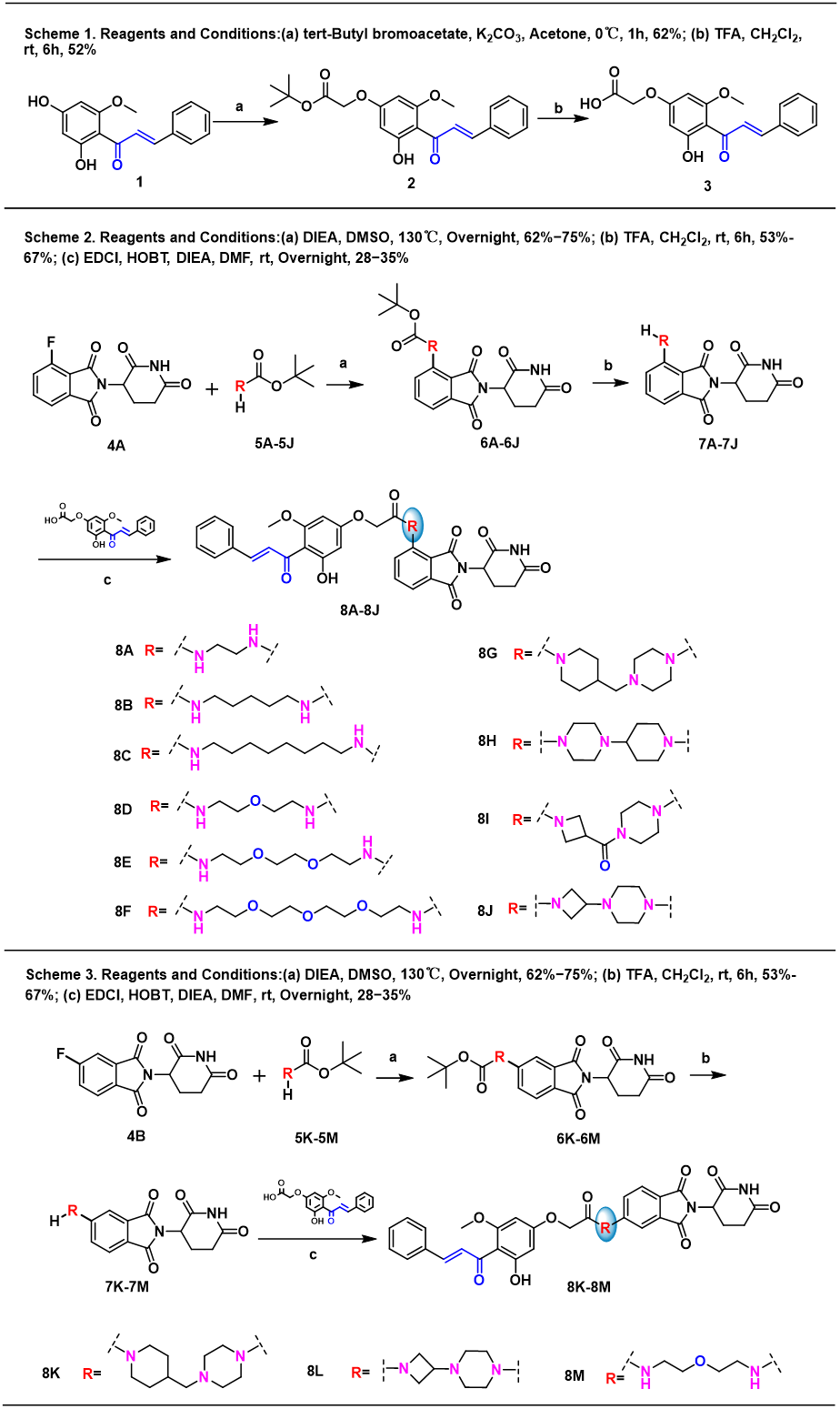
**

**Synthetic methods**

**General Procedure for the Preparation of Key Intermediate 3.** **CAD** (500 mg, 1.0 eq) was dissolved in DMF (10 mL), followed by the addition of tert-butyl bromoacetate (270 μL, 1.0 eq) and potassium carbonate (510 mg, 2.0 eq). The reaction was stirred at 0°C for 1 h. Upon completion, the mixture was diluted with equal volumes of dichloromethane and water, extracted, and separated. The organic layer was washed with saturated sodium chloride solution, dried over anhydrous sodium sulfate, and concentrated under reduced pressure using a rotary evaporator. The crude product was purified by silica gel column chromatography to afford intermediate 2 in 62% yield. Intermediate 2 was then treated with trifluoroacetic acid/dichloromethane (1:2, v/v) at room temperature for 6 h. After rotary evaporation, the residue was recrystallized with acetonitrile to yield key intermediate 3 (52% yield).

**General Procedure for the Preparation of Intermediate 7.**

2-(2,6-Dioxopiperidin-3-yl)-4-fluoroisoindole-1,3-dione (1.0 eq) was dissolved in DMSO, followed by the addition of N-Boc-diamines bearing various alkyl, alkoxy, or rigid linkers (1.2 eq) and N, N-diisopropylethylamine (DIEA, 5.0 eq). The reaction was heated at 130°C overnight. After cooling to room temperature, the mixture was diluted with equal volumes of dichloromethane and water, extracted, and separated. The organic layer was washed with saturated sodium chloride solution, dried over anhydrous sodium sulfate, and concentrated under reduced pressure. Purification via silica gel column chromatography furnished intermediate 6 (62–75% yield). Subsequent treatment with trifluoroacetic acid/dichloromethane (1:2, v/v) at room temperature for 6 h, followed by rotary evaporation and recrystallization with acetonitrile, yielded intermediate 7 (53–67% yield).

**General Procedure for the Preparation of PROTACs 8A-8M.** Key intermediate 3 (1.0 eq), 1-ethyl-(3-dimethylaminopropyl) carbodiimide hydrochloride (EDCI, 2.0 eq), 1-hydroxybenzotriazole (HOBT, 2.0 eq), and DIEA (2.0 eq) were dissolved in DMF and stirred at room temperature for 10 min. Intermediate 7 (1.2 eq) was then added, and the reaction was stirred at room temperature overnight. The crude product was purified by column chromatography to afford yellow solid powders of PROTACs 8A-8M (28–35% yield).

**8A:** yellow solid, 42 mg, ^1^H NMR (600 MHz, DMSO-*d*_6_) *δ* 13.10 (s, 1H), 11.10 (s, 1H), 8.39 (t, *J* = 5.6 Hz, 1H), 7.73 (d, *J* = 3.6 Hz, 1H), 7.72 (s, 1H), 7.69 (s, 1H), 7.63 (d, *J* = 15.7 Hz, 1H), 7.61 – 7.57 (m, 1H), 7.46 (s, 1H), 7.46 (s, 2H), 7.21 (d, *J* = 8.6 Hz, 1H), 7.03 (d, *J* = 7.0 Hz, 1H), 6.78 (t, *J* = 6.0 Hz, 1H), 6.23 (d, *J* = 2.0 Hz, 1H), 6.12 (d, *J* = 2.0 Hz, 1H), 5.06 (dd, *J* = 12.8, 5.4 Hz, 1H), 4.57 (d, *J* = 7.8 Hz, 2H), 3.89 (s, 3H), 3.46 – 3.43 (m, 2H), 3.38 (d, *J* = 5.9 Hz, 2H), 2.89 (s, 1H), 2.74 (s, 1H), 2.59 (d, *J* = 18.0 Hz, 1H), 2.03 (dd, *J* = 9.1, 3.7 Hz, 1H). ^13^C NMR (101 MHz, DMSO-*d*_6_) *δ* 192.9, 173.2, 170.5, 168.0, 167.7, 165.0, 163.9, 162.7, 162.0, 146.7, 143.0, 136.6, 135.1, 132.6, 130.9, 129.5, 128.9, 127.9, 117.6, 111.0, 109.7, 107.4, 95.2, 91.9, 67.4, 56.6, 48.9, 41.8, 38.3, 31.4, 26.8. HRMS (ESI): C_33_H_30_N_4_O_9_ [M+H]^+^, *m/z*: calculated: 627.2086, found: 627.1976.

**8B:** yellow solid, 30 mg, ^1^H NMR (400 MHz, DMSO-*d*_6_) *δ* 13.19 (d, *J* = 7.9 Hz, 1H), 11.11 (s, 1H), 8.16 (t, *J* = 5.7 Hz, 1H), 7.74 (dd, *J* = 7.5, 3.9 Hz, 2H), 7.69 (d, *J* = 21.6 Hz, 2H), 7.61 – 7.55 (m, 1H), 7.48 (s, 1H), 7.46 (d, *J* = 2.4 Hz, 2H), 7.09 (d, *J* = 8.6 Hz, 1H), 7.03 (d, *J* = 7.0 Hz, 1H), 6.54 (t, *J* = 5.7 Hz, 1H), 6.26 (d, *J* = 2.0 Hz, 1H), 6.13 (d, *J* = 2.1 Hz, 1H), 5.07 (dd, *J* = 12.9, 5.4 Hz, 1H), 4.59 (d, *J* = 9.4 Hz, 2H), 3.91 (s, 3H), 3.29 (dd, *J* = 13.0, 6.5 Hz, 2H), 3.18 (q, *J* = 6.5 Hz, 2H), 2.88 (dd, *J* = 17.4, 5.5 Hz, 1H), 2.65 – 2.54 (m, 2H), 2.03 (dd, *J* = 11.5, 3.9 Hz, 1H), 1.59 (dd, *J* = 14.4, 7.1 Hz, 2H), 1.51 (dd, *J* = 14.2, 7.1 Hz, 2H), 1.36 (d, *J* = 7.0 Hz, 2H). ^13^C NMR (101 MHz, DMSO-*d*_6_) *δ* 192.9, 173.3, 170.5, 169.4, 167.7, 167.2, 165.2, 164.1, 162.1, 146.8, 143.0, 136.7, 135.2, 132.6, 130.9, 129.5, 128.9, 127.9, 117.6, 110.8, 109.5, 107.4, 95.2, 92.1, 67.5, 56.7, 49.0, 42.3, 38.6, 31.4, 29.2, 28.8, 24.1, 22.6. HRMS (ESI): C_36_H_36_N_4_O_9_ [M+H]^+^, *m/z*: calculated: 669.2556, found: 669.2427.

**8C:** yellow solid, 44 mg, ^1^H NMR (400 MHz, DMSO-*d*_6_) *δ* 13.19 (s, 1H), 11.11 (s, 1H), 8.12 (t, *J* = 5.8 Hz, 1H), 7.74 (d, *J* = 8.0 Hz, 1H), 7.72 (d, *J* = 4.2 Hz, 2H), 7.64 (d, *J* = 15.7 Hz, 1H), 7.60 – 7.54 (m, 1H), 7.48 – 7.43 (m, 3H), 7.07 (d, *J* = 8.6 Hz, 1H), 7.01 (d, *J* = 7.0 Hz, 1H), 6.51 (t, *J* = 5.8 Hz, 1H), 6.23 (dd, *J* = 8.2, 2.3 Hz, 1H), 6.11 (d, *J* = 2.2 Hz, 1H), 5.06 (dd, *J* = 12.8, 5.4 Hz, 1H), 4.59 – 4.51 (m, 2H), 3.90 (s, 3H), 3.26 (dd, *J* = 13.3, 6.7 Hz, 2H), 3.13 (dd, *J* = 12.9, 6.6 Hz, 2H), 2.90 – 2.83 (m, 1H), 2.60 (d, *J* = 13.7 Hz, 1H), 2.58 – 2.53 (m, 1H), 2.03 (dd, *J* = 8.9, 3.7 Hz, 1H), 1.55 (dd, *J* = 13.6, 6.8 Hz, 2H), 1.49 – 1.39 (m, 2H), 1.27 (d, *J* = 10.7 Hz, 8H). ^13^C NMR (101 MHz, DMSO-*d*_6_) *δ* 192.9, 173.2, 170.5, 169.4, 167.7, 167.1, 165.2, 164.1, 162.1, 146.8, 143.0, 136.7, 135.1, 132.6, 130.9, 129.5, 128.9, 127.9, 117.6, 110.8, 109.4, 107.3, 95.2, 92.0, 67.5, 56.7, 48.9, 42.2, 38.7, 31.4, 29.5, 29.2, 29.1, 26.7, 22.6, 22.5, 14.4. HRMS (ESI): C_39_H_42_N_4_O_9_ [M+H]^+^, *m/z*: calculated: 711.3025, found: 711.2895.

**8D:** yellow solid, 35 mg, ^1^H NMR (400 MHz, DMSO-*d*_6_) *δ* 13.17 (s, 1H), 11.11 (s, 1H), 8.19 (t, *J* = 5.7 Hz, 1H), 7.75 – 7.73 (m, 2H), 7.71 (s, 1H), 7.66 (s, 1H), 7.61 – 7.56 (m, 1H), 7.48 (s, 1H), 7.46 (d, *J* = 2.3 Hz, 2H), 7.15 (d, *J* = 8.6 Hz, 1H), 7.05 (d, *J* = 7.0 Hz, 1H), 6.62 (t, *J* = 5.6 Hz, 1H), 6.24 (d, *J* = 2.1 Hz, 1H), 6.12 (d, *J* = 2.1 Hz, 1H), 5.06 (dd, *J* = 12.9, 5.3 Hz, 1H), 4.59 (s, 2H), 3.90 (s, 3H), 3.64 (t, *J* = 5.3 Hz, 2H), 3.54 (t, *J* = 5.8 Hz, 2H), 3.48 (t, *J* = 5.3 Hz, 2H), 2.94 – 2.81 (m, 1H), 2.63 – 2.53 (m, 2H), 2.06 – 2.01 (m, 1H). ^13^C NMR (101 MHz, DMSO-*d*_6_) *δ* 192.9, 173.2, 170.5, 169.4, 167.7, 167.5, 165.2, 164.1, 162.1, 146.8, 142.9, 136.7, 135.2, 132.5, 130.9, 129.5, 128.9, 127.9, 117.9, 111.1, 109.7, 107.4, 95.2, 92.0, 69.1, 69.0, 67.4, 56.7, 49.0, 42.1, 38.7, 31.4, 22.6. HRMS (ESI): C_35_H_34_N_4_O_10_ [M+H]^+^, *m/z*: calculated: 671.2348, found: 671.2233.

**8E:** yellow solid, 52 mg, ^1^H NMR (600 MHz, DMSO-*d*_6_) *δ* 13.15 (s, 1H), 11.09 (s, 1H), 8.14 (t, *J* = 5.5 Hz, 1H), 7.73 (s, 1H), 7.72 (s, 1H), 7.70 (s, 1H), 7.63 (d, *J* = 15.7 Hz, 1H), 7.57 (t, *J* = 7.8 Hz, 1H), 7.46 (d, *J* = 4.2 Hz, 3H), 7.12 (d, *J* = 8.6 Hz, 1H), 7.03 (d, *J* = 7.0 Hz, 1H), 6.60 (t, *J* = 5.5 Hz, 1H), 6.23 (d, *J* = 7.8 Hz, 1H), 6.11 (s, 1H), 5.06 (dd, *J* = 12.8, 5.4 Hz, 1H), 4.56 (d, *J* = 8.8 Hz, 2H), 3.89 (s, 3H), 3.62 (t, *J* = 5.3 Hz, 2H), 3.56 (dd, *J* = 14.8, 4.8 Hz, 4H), 3.47 (dt, *J* = 15.6, 5.6 Hz, 4H), 3.32 – 3.29 (m, 2H), 2.87 (dd, *J* = 17.5, 4.8 Hz, 1H), 2.59 (d, *J* = 18.6 Hz, 1H), 2.57 – 2.52 (m, 1H), 2.06 – 2.00 (m, 1H). ^13^C NMR (101 MHz, DMSO-*d*_6_) *δ* 192.9, 173.2, 170.5, 169.4, 167.7, 167.4, 165.1, 164.0, 162.1, 146.8, 143.0, 136.6, 135.2, 132.5, 130.9, 129.5, 128.9, 127.9, 117.8, 111.1, 109.7, 107.3, 95.1, 92.0, 70.1, 70.0, 69.3, 67.3, 56.6, 49.0, 42.1, 38.8, 31.4, 22.6, 14.4. HRMS (ESI): C_37_H_38_N_4_O_11_ [M+H]^+^, *m/z*: calculated: 715.2610, found: 715.2477.

**8F:** yellow solid, 37 mg, ^1^H NMR (600 MHz, DMSO-*d*_6_) *δ* 13.15 (s, 1H), 11.10 (s, 1H), 8.15 (t, *J* = 5.6 Hz, 1H), 7.72 (dd, *J* = 9.9, 5.8 Hz, 3H), 7.64 (d, *J* = 15.7 Hz, 1H), 7.59 – 7.55 (m, 1H), 7.48 – 7.44 (m, 3H), 7.13 (d, *J* = 8.6 Hz, 1H), 7.04 (d, *J* = 7.0 Hz, 1H), 6.59 (t, *J* = 5.6 Hz, 1H), 6.24 (d, *J* = 2.0 Hz, 1H), 6.11 (d, *J* = 2.1 Hz, 1H), 5.06 (dd, *J* = 12.8, 5.4 Hz, 1H), 4.57 (s, 2H), 3.90 (s, 3H), 3.61 (t, *J* = 5.4 Hz, 2H), 3.58 – 3.53 (m, 4H), 3.51 (q, *J* = 7.4 Hz, 4H), 3.46 (t, *J* = 5.7 Hz, 4H), 3.30 (dd, *J* = 11.6, 5.8 Hz, 2H), 2.92 – 2.84 (m, 1H), 2.59 (d, *J* = 17.9 Hz, 1H), 2.55 (dd, *J* = 13.1, 4.3 Hz, 1H), 2.03 (dd, *J* = 9.1, 3.6 Hz, 1H). ^13^C NMR (101 MHz, DMSO-*d*_6_) *δ* 192.9, 173.2, 170.5, 169.4, 167.7, 167.4, 165.2, 164.0, 162.1, 146.8, 143.0, 136.6, 135.2, 132.5, 130.9, 129.5, 128.9, 127.9, 117.8, 111.1, 109.6, 107.3, 95.1, 92.0, 70.2, 70.0, 69.3, 69.2, 67.3, 56.7, 49.0, 42.1, 38.8, 31.4, 22.6, 22.5, 14.4. HRMS (ESI): C_39_H_42_N_4_O_12_ [M+H]^+^, *m/z*: calculated: 759.2872, found: 759.2740.

**8G:** yellow solid, 48 mg, ^1^H NMR (600 MHz, DMSO-*d*_6_) *δ* 13.33 (s, 1H), 11.09 (s, 1H), 7.77 (d, *J* = 15.7 Hz, 1H), 7.73 (dd, *J* = 8.6, 3.6 Hz, 2H), 7.70 (d, *J* = 7.7 Hz, 1H), 7.67 – 7.64 (m, 1H), 7.46 (d, *J* = 6.2 Hz, 3H), 7.36 (d, *J* = 7.1 Hz, 1H), 7.34 (d, *J* = 8.5 Hz, 1H), 6.19 (d, *J* = 2.0 Hz, 1H), 6.11 (d, *J* = 2.1 Hz, 1H), 5.10 (dd, *J* = 12.8, 5.5 Hz, 1H), 4.93 (q, *J* = 14.6 Hz, 2H), 4.33 (d, *J* = 12.6 Hz, 1H), 3.91 (s, 3H), 3.83 (d, *J* = 12.8 Hz, 1H), 3.31 (s, 4H), 3.05 (t, *J* = 12.2 Hz, 1H), 2.92 – 2.84 (m, 1H), 2.64 (t, *J* = 10.0 Hz, 1H), 2.63 – 2.57 (m, 2H), 2.55 (d, *J* = 3.0 Hz, 4H), 2.22 (d, *J* = 6.9 Hz, 2H), 2.07 – 2.00 (m, 1H), 1.85 (s, 1H), 1.78 (s, 2H), 1.19 – 1.10 (m, 1H), 0.97 (d, *J* = 10.1 Hz, 1H). ^13^C NMR (101 MHz, DMSO-*d*_6_) *δ* 192.7, 173.2, 170.4, 167.5, 166.7, 165.6, 165.2, 164.9, 162.2, 150.1, 142.8, 136.3, 135.2, 134.1, 130.9, 129.5, 128.9, 127.9, 124.1, 116.9, 115.2, 107.0, 95.1, 92.1, 66.5, 64.0, 56.7, 53.5, 51.0, 49.2, 44.6, 41.8, 33.0, 31.4, 30.5, 22.5, 14.4. HRMS (ESI): C_41_H_43_N_5_O_9_ [M+H]^+^, *m/z*: calculated: 750.3134, found: 750.2296.

**8H:** yellow solid, 62 mg, ^1^H NMR (600 MHz, DMSO-*d*_6_) *δ* 13.31 (s, 1H), 11.10 (s, 1H), 7.76 (d, *J* = 15.7 Hz, 1H), 7.75 – 7.72 (m, 2H), 7.69 (d, *J* = 7.8 Hz, 1H), 7.67 (s, 1H), 7.46 (s, 1H), 7.46 (d, *J* = 1.2 Hz, 2H), 7.34 (d, *J* = 2.7 Hz, 1H), 7.33 (s, 1H), 6.20 (d, *J* = 2.0 Hz, 1H), 6.12 (d, *J* = 2.1 Hz, 1H), 5.10 (dd, *J* = 12.8, 5.5 Hz, 1H), 4.94 (s, 2H), 3.90 (d, *J* = 7.0 Hz, 3H), 3.75 (d, *J* = 10.8 Hz, 2H), 3.47 (s, 4H), 3.17 (d, *J* = 5.2 Hz, 1H), 2.90 (d, *J* = 5.1 Hz, 1H), 2.88 (dd, *J* = 17.7, 7.1 Hz, 3H), 2.61 (d, *J* = 7.3 Hz, 1H), 2.57 (d, *J* = 22.5 Hz, 3H), 2.48 (s, 1H), 2.06 – 2.01 (m, 1H), 1.87 (d, *J* = 10.9 Hz, 2H), 1.65 (d, *J* = 11.2 Hz, 2H). ^13^C NMR (101 MHz, DMSO-*d*_6_) *δ* 192.8, 173.3, 170.5, 167.5, 166.7, 165.5, 165.4, 164.8, 162.2, 150.2, 142.8, 136.2, 135.2, 134.1, 130.9, 129.5, 128.9, 127.9, 124.4, 116.8, 115.0, 107.0, 95.1, 92.1, 66.3, 60.9, 56.7, 50.8, 49.3, 49.2, 49.0, 48.9, 45.0, 42.2, 40.6, 40.4, 40.2, 39.9, 39.7, 39.5, 39.3, 31.4, 28.3, 22.5. HRMS (ESI): C_40_H_41_N_5_O_9_ [M+H]^+^, *m/z*: calculated: 736.2978, found: 736.2842.

**8I:** yellow solid, 53 mg, ^1^H NMR (600 MHz, DMSO-*d*_6_) *δ* 13.18 (s, 1H), 11.10 (s, 1H), 7.75 – 7.71 (m, 4H), 7.64 (d, *J* = 15.7 Hz, 1H), 7.49 – 7.44 (m, 3H), 7.41 (d, *J* = 7.1 Hz, 1H), 7.36 (d, *J* = 8.4 Hz, 1H), 6.21 (d, *J* = 2.2 Hz, 1H), 6.11 (d, *J* = 2.2 Hz, 1H), 5.12 (dd, *J* = 12.9, 5.5 Hz, 1H), 4.72 (s, 2H), 4.44 (dd, *J* = 12.2, 6.1 Hz, 2H), 4.15 (t, *J* = 9.3 Hz, 1H), 4.09 – 4.04 (m, 1H), 3.90 (s, 3H), 3.84 (dt, *J* = 15.1, 7.6 Hz, 1H), 3.74 – 3.65 (m, 2H), 3.50 (d, *J* = 4.1 Hz, 2H), 3.32 – 3.24 (m, 4H), 2.89 (ddd, *J* = 17.1, 14.1, 5.4 Hz, 1H), 2.64 – 2.58 (m, 1H), 2.54 (dd, *J* = 13.7, 4.5 Hz, 1H), 2.04 (dd, *J* = 9.1, 3.7 Hz, 1H). ^13^C NMR (151 MHz, DMSO-*d*_6_) *δ* 192.9, 173.2, 170.4, 169.7, 167.4, 167.2, 166.8, 165.2, 164.2, 162.2, 149.8, 142.9, 136.4, 135.2, 134.0, 130.9, 129.5, 128.9, 127.9, 124.3, 117.4, 115.7, 107.3, 94.9, 91.9, 65.8, 56.7, 52.5, 51.3, 50.6, 49.3, 45.0, 41.8, 40.5, 31.52, 31.4, 22.5. HRMS (ESI): C_39_H_37_N_5_O_10_ [M+H]^+^, *m/z*: calculated: 736.2614, found: 736.2478.

**8J:** yellow solid, 49 mg, ^1^H NMR (600 MHz, DMSO-*d*_6_) *δ* 13.12 (s, 1H), 11.09 (s, 1H), 7.71 (dd, *J* = 14.5, 9.5 Hz, 4H), 7.62 (d, *J* = 15.7 Hz, 1H), 7.45 (d, *J* = 4.5 Hz, 3H), 7.36 (t, *J* = 8.2 Hz, 2H), 6.21 (d, *J* = 1.6 Hz, 1H), 6.13 (s, 1H), 5.09 (dd, *J* = 12.8, 5.4 Hz, 1H), 4.73 (s, 2H), 4.31 (t, *J* = 8.1 Hz, 1H), 4.21 – 4.13 (m, 1H), 4.02 – 3.95 (m, 1H), 3.90 (s, 3H), 3.82 (dd, *J* = 9.6, 4.7 Hz, 1H), 3.33 (s, 4H), 3.30 – 3.24 (m, 1H), 2.94 – 2.83 (m, 1H), 2.62 – 2.56 (m, 2H), 2.52 (d, *J* = 11.5 Hz, 4H), 2.07 – 2.00 (m, 1H). ^13^C NMR (151 MHz, DMSO-*d*_6_) *δ* 192.9, 173.2, 170.4, 167.5, 167.3, 166.7, 165.0, 164.2, 162.0, 150.0, 142.5, 136.3, 135.2, 134.1, 130.9, 129.5, 128.9, 128.0, 124.2, 117.0, 115.3, 107.4, 94.9, 91.9, 65.8, 56.7, 54.6, 54.1, 52.2, 50.6, 49.4, 49.2, 40.5, 40.4, 40.2, 40.1, 40.0, 39.8, 39.7, 39.5, 31.4, 22.5. HRMS (ESI): C_38_H_37_N_5_O_9_ [M+H]^+^, *m/z*: calculated: 708.2665, found: 708.2659.

**8K:** yellow solid, 53 mg, ^1^H NMR (600 MHz, DMSO-*d*_6_) *δ* 13.34 (s, 1H), 11.09 (s, 1H), 7.77 (d, *J* = 15.7 Hz, 1H), 7.73 (dd, *J* = 7.1, 2.1 Hz, 2H), 7.69 – 7.64 (m, 2H), 7.48 – 7.46 (m, 1H), 7.45 (t, *J* = 5.3 Hz, 2H), 7.34 (s, 1H), 7.26 (dd, *J* = 8.6, 1.9 Hz, 1H), 6.19 (d, *J* = 2.2 Hz, 1H), 6.11 (d, *J* = 2.2 Hz, 1H), 5.08 (dd, *J* = 12.8, 5.4 Hz, 1H), 4.93 (q, *J* = 14.6 Hz, 2H), 4.33 (d, *J* = 12.4 Hz, 1H), 3.91 (s, 3H), 3.83 (d, *J* = 13.5 Hz, 1H), 3.44 (s, 4H), 3.06 (t, *J* = 12.5 Hz, 1H), 2.92 – 2.84 (m, 1H), 2.65 (t, *J* = 12.1 Hz, 1H), 2.62 – 2.57 (m, 1H), 2.55 (dd, *J* = 13.1, 8.7 Hz, 1H), 2.49 – 2.46 (m, 2H), 2.20 (d, *J* = 7.1 Hz, 2H), 2.06 – 2.00 (m, 1H), 1.84 (d, *J* = 3.3 Hz, 1H), 1.78 (s, 2H), 1.27 (dd, *J* = 15.6, 8.1 Hz, 2H), 1.14 (dd, *J* = 21.4, 11.1 Hz, 1H), 0.97 (dd, *J* = 20.8, 11.4 Hz, 1H). ^13^C NMR (101 MHz, DMSO-*d*_6_) *δ* 192.7, 173.2, 170.5, 168.0, 167.4, 165.6, 165.2, 164.9, 162.2, 155.7, 142.8, 135.2, 134.3, 130.9, 129.5, 128.9, 127.9, 125.3, 118.7, 118.2, 108.3, 107.0, 95.1, 92.1, 66.5, 63.9, 56.7, 53.1, 49.2, 47.3, 44.6, 41.8, 33.0, 31.4, 31.3, 30.5, 22.6, 22.5, 14.4. HRMS (ESI): C_41_H_43_N_5_O_9_ [M+H]^+^, *m/z*: calculated: 750.3134, found: 750.3129.

**8L:** yellow solid, 1.53 g, ^1^H NMR (600 MHz, DMSO-*d*_6_) *δ* 13.22 (s, 1H), 11.09 (s, 1H), 7.76 – 7.72 (m, 3H), 7.69 (d, *J* = 8.5 Hz, 1H), 7.65 (d, *J* = 15.7 Hz, 1H), 7.47 (t, *J* = 3.0 Hz, 1H), 7.45 (d, *J* = 1.8 Hz, 2H), 7.37 (d, *J* = 1.9 Hz, 1H), 7.28 (dd, *J* = 8.7, 2.2 Hz, 1H), 6.22 (d, *J* = 2.3 Hz, 1H), 6.11 (d, *J* = 2.3 Hz, 1H), 5.08 (dt, *J* = 14.6, 7.4 Hz, 1H), 4.72 (d, *J* = 15.1 Hz, 2H), 4.31 (t, *J* = 8.1 Hz, 1H), 4.16 (dd, *J* = 9.0, 4.9 Hz, 1H), 3.99 (dd, *J* = 9.6, 7.5 Hz, 1H), 3.91 (s, 3H), 3.86 – 3.81 (m, 1H), 3.48 (t, *J* = 4.8 Hz, 4H), 3.27 – 3.21 (m, 1H), 2.89 (ddd, *J* = 16.9, 13.9, 5.4 Hz, 1H), 2.64 – 2.53 (m, 2H), 2.50 – 2.43 (m, 4H), 2.06 – 1.99 (m, 1H). ^13^C NMR (151 MHz, DMSO-*d*_6_) *δ* 192.8, 173.2, 170.5, 168.0, 167.4, 167.2, 165.3, 164.3, 162.2, 155.6, 142.9, 135.2, 134.3, 130.9, 129.5, 128.9, 127.9, 125.3, 118.9, 118.3, 108.5, 107.3, 94.9, 92.0, 65.8, 56.7, 54.4, 54.1, 52.2, 49.2, 49.0, 47.0, 40.5, 31.4, 22.6. HRMS (ESI): C_38_H_37_N_5_O_9_ [M+H]^+^, *m/z*: calculated: 708.2665, found: 708.2666.

**8M:** yellow solid, 27 mg, ^1^H NMR (600 MHz, DMSO-*d*_6_) *δ* 13.17 (s, 1H), 11.06 (s, 1H), 8.21 (t, *J* = 5.7 Hz, 1H), 7.73 – 7.69 (m, 3H), 7.65 – 7.61 (m, 1H), 7.54 (d, *J* = 8.4 Hz, 1H), 7.46 (dd, *J* = 4.9, 1.8 Hz, 3H), 7.08 (t, *J* = 5.3 Hz, 1H), 7.00 (d, *J* = 1.3 Hz, 1H), 6.89 (dd, *J* = 8.4, 1.8 Hz, 1H), 6.23 (d, *J* = 2.2 Hz, 1H), 6.11 (d, *J* = 2.2 Hz, 1H), 5.03 (dd, *J* = 12.8, 5.4 Hz, 1H), 4.58 (d, *J* = 8.7 Hz, 2H), 3.90 (d, *J* = 8.6 Hz, 3H), 3.61 (t, *J* = 5.4 Hz, 2H), 3.52 (t, *J* = 5.8 Hz, 2H), 3.35 (dd, *J* = 12.3, 6.2 Hz, 4H), 2.87 (ddd, *J* = 17.0, 13.9, 5.4 Hz, 1H), 2.61 – 2.52 (m, 2H), 2.02 – 1.95 (m, 1H). ^13^C NMR (151 MHz, DMSO-*d*_6_) *δ* 192.8, 173.2, 170.6, 168.1, 167.6, 167.6, 165.2, 164.1, 162.1, 154.8, 142.9, 135.2, 134.6, 130.9, 129.5, 128.9, 127.9, 125.4, 116.6, 107.3, 95.2, 92.0, 69.3, 68.7, 67.4, 56.6, 49.0, 42.8, 38.6, 31.4, 22.7. HRMS (ESI): C_35_H_34_N_4_O_10_ [M+H]^+^, *m/z*: calculated: 671.2348, found: 671.2342.

**CAD-1**: yellow solid, 27 mg, ^1^H NMR (600 MHz, CDCl_3_) δ 13.56 (s, 1H), 7.91 – 7.80 (m, 2H), 7.64 (dd, *J* = 7.3, 2.0 Hz, 2H), 7.49 – 7.41 (m, 3H), 6.41 (d, *J* = 2.1 Hz, 1H), 6.25 (d, *J* = 2.1 Hz, 1H), 3.97 (s, 3H), 2.57 (t, *J* = 7.4 Hz, 2H), 1.89 – 1.77 (m, 2H), 1.08 (t, *J* = 7.4 Hz, 3H).

^1^H NMR spectrum and ^13^C NMR spectrum of 8A

^1^H NMR spectrum and ^13^C NMR spectrum of 8B

^1^H NMR spectrum and ^13^C NMR spectrum of 8C

^1^H NMR spectrum and ^13^C NMR spectrum of 8D

^1^H NMR spectrum and ^13^C NMR spectrum of 8E

^1^H NMR spectrum and ^13^C NMR spectrum of 8F

^1^H NMR spectrum and ^13^C NMR spectrum of 8G

^1^H NMR spectrum and ^13^C NMR spectrum of 8H

^1^H NMR spectrum and ^13^C NMR spectrum of 8I

^1^H NMR spectrum and ^13^C NMR spectrum of 8J

^1^H NMR spectrum and ^13^C NMR spectrum of 8K

^1^H NMR spectrum and ^13^C NMR spectrum of 8L

^1^H NMR spectrum of **CAD-1**

^1^H NMR spectrum and ^13^C NMR spectrum of **^1^**

HPLC-UV Spectra of **8A**

|  | RT (min) | Area | Height | Area% |
| --- | --- | --- | --- | --- |
| 1 | 9.036 | 366526 | 55027 | 96.327 |
| 2 | 10.427 | 13976 | 2349 | 3.673 |
| Total |  | 380503 | 57376 | 100.000 |

HPLC-UV Spectra of **8B**

|  | RT (min) | Area | Height | Area% |
| --- | --- | --- | --- | --- |
| 1 | 9.450 | 327076 | 50003 | 96.589 |
| 2 | 10.206 | 11323 | 2375 | 3.344 |
| 3 | 10.733 | 227 | 83 | 0.067 |
| Total |  | 338626 | 52462 | 100.000 |

HPLC-UV Spectra of **8C**

|  | RT (min) | Area | Height | Area% |
| --- | --- | --- | --- | --- |
| 1 | 8.791 | 12107 | 1840 | 2.336 |
| 2 | 10.029 | 497127 | 75967 | 95.926 |
| 3 | 11.191 | 4635 | 649 | 0.894 |
| 4 | 11.317 | 4373 | 527 | 0.844 |
| Total |  | 518242 | 78984 | 100.000 |

HPLC-UV Spectra of **8D**

|  | RT (min) | Area | Height | Area% |
| --- | --- | --- | --- | --- |
| 1 | 9.102 | 358378 | 53968 | 95.149 |
| 2 | 10.525 | 18271 | 3554 | 4.851 |
| Total |  | 376649 | 57523 | 100.000 |

HPLC-UV Spectra of **8E**

|  | RT (min) | Area | Height | Area% |
| --- | --- | --- | --- | --- |
| 1 | 9.151 | 353356 | 52803 | 95.709 |
| 2 | 10.499 | 15844 | 3000 | 4.291 |
| Total |  | 369200 | 55803 | 100.000 |

HPLC-UV Spectra of **8F**

|  | RT (min) | Area | Height | Area% |
| --- | --- | --- | --- | --- |
| 1 | 9.142 | 391289 | 57750 | 99.290 |
| 2 | 10.056 | 1171 | 197 | 0.297 |
| 3 | 10.505 | 1626 | 163 | 0.413 |
| Total |  | 394086 | 58111 | 100.000 |

HPLC-UV Spectra of **8G**

|  | RT (min) | Area | Height | Area% |
| --- | --- | --- | --- | --- |
| 1 | 7.60 | 432368 | 75405 | 100.000 |
| Total |  | 432368 | 75405 | 100.000 |

HPLC-UV Spectra of **8H**

|  | RT (min) | Area | Height | Area% |
| --- | --- | --- | --- | --- |
| 1 | 7.608 | 444670 | 77821 | 95.012 |
| 2 | 8.496 | 21181 | 4112 | 4.526 |
| 3 | 9.098 | 2163 | 563 | 0.462 |
| Total |  | 468014 | 82496 | 100.000 |

HPLC Spectra of **8I**

|  | RT (min) | Area | Height | Area% |
| --- | --- | --- | --- | --- |
| 1 | 8.968 | 433867 | 64008 | 100.000 |
| Total |  | 433867 | 64008 | 100.000 |

HPLC Spectra of **8J**

|  | RT (min) | Area | Height | Area% |
| --- | --- | --- | --- | --- |
| 1 | 8.045 | 284690 | 40516 | 98.189 |
| 2 | 9.208 | 3497 | 527 | 1.206 |
| 3 | 9.897 | 1754 | 225 | 0.605 |
| Total |  | 289941 | 41269 | 100.000 |

HPLC-UV Spectra of **8K**

|  | RT (min) | Area | Height | Area% |
| --- | --- | --- | --- | --- |
| 1 | 7.571 | 290975 | 51056 | 98.395 |
| 2 | 8.453 | 4746 | 1036 | 1.605 |
| Total |  | 295721 | 52092 | 100.000 |

HPLC-UV Spectra of **8L**

|  | RT (min) | Area | Height | Area% |
| --- | --- | --- | --- | --- |
| 1 | 8.322 | 498679 | 72563 | 100.000 |
| Total |  | 498679 | 72563 | 100.000 |

HPLC-UV Spectra of **8M**

|  | RT (min) | Area | Height | Area% |
| --- | --- | --- | --- | --- |
| 1 | 8.618 | 23509 | 4530 | 7.005 |
| 2 | 8.865 | 308274 | 47987 | 91.854 |
| 3 | 10.297 | 3829 | 840 | 1.141 |
| Total |  | 335612 | 53358 | 100.000 |

HRMS spectra of **8A**


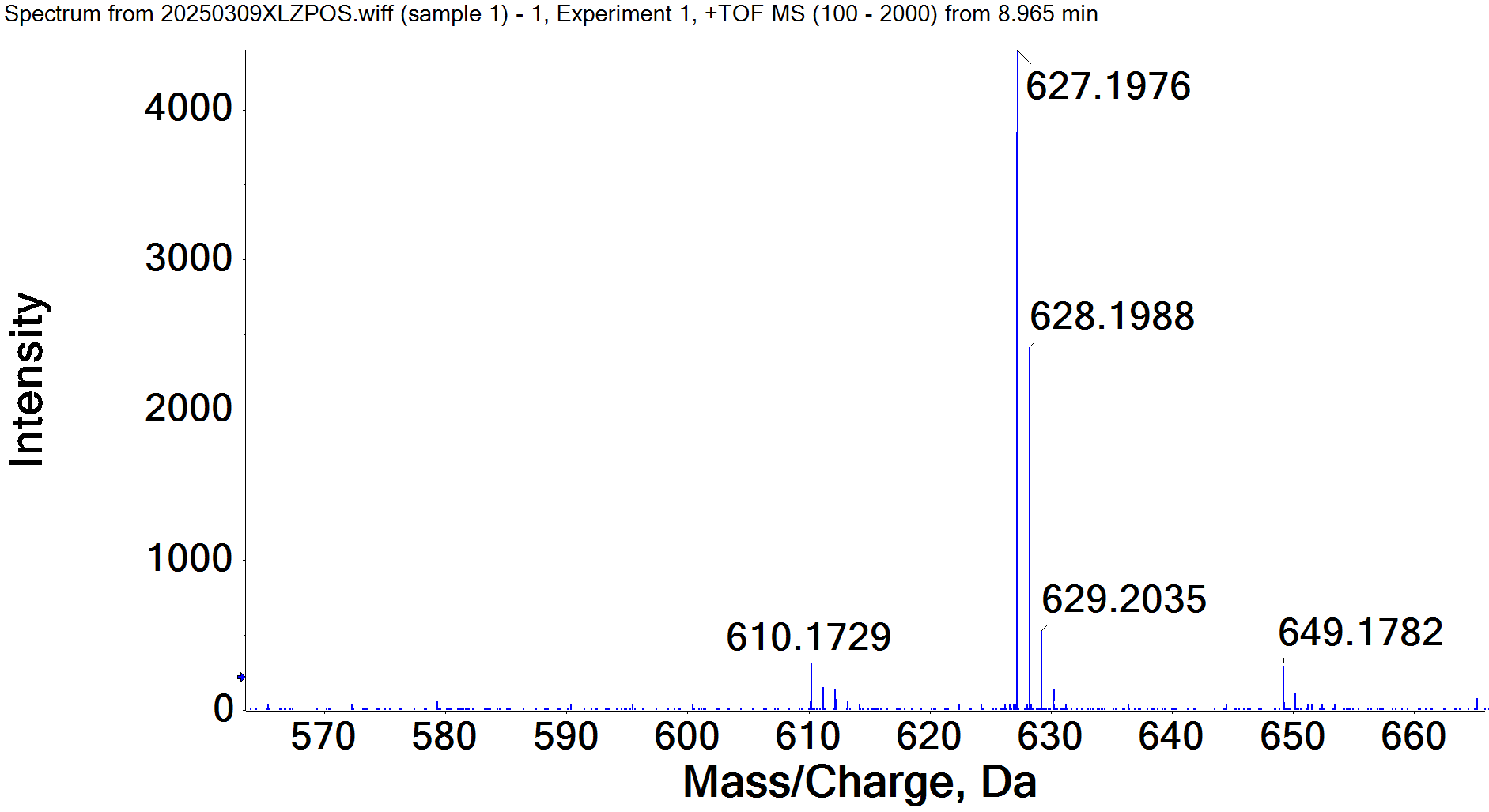


HRMS spectra of **8B**


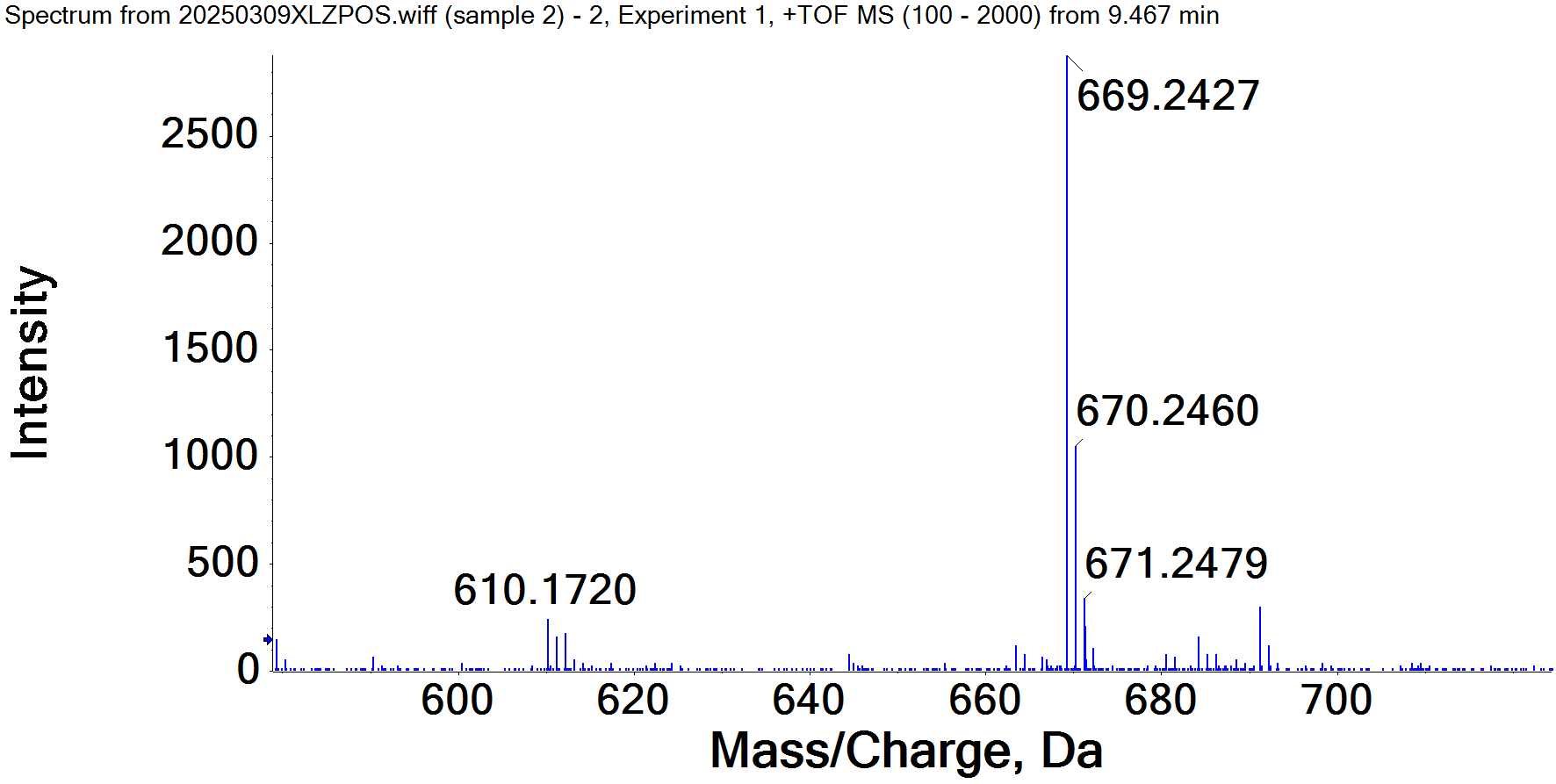


HRMS spectra of **8C**


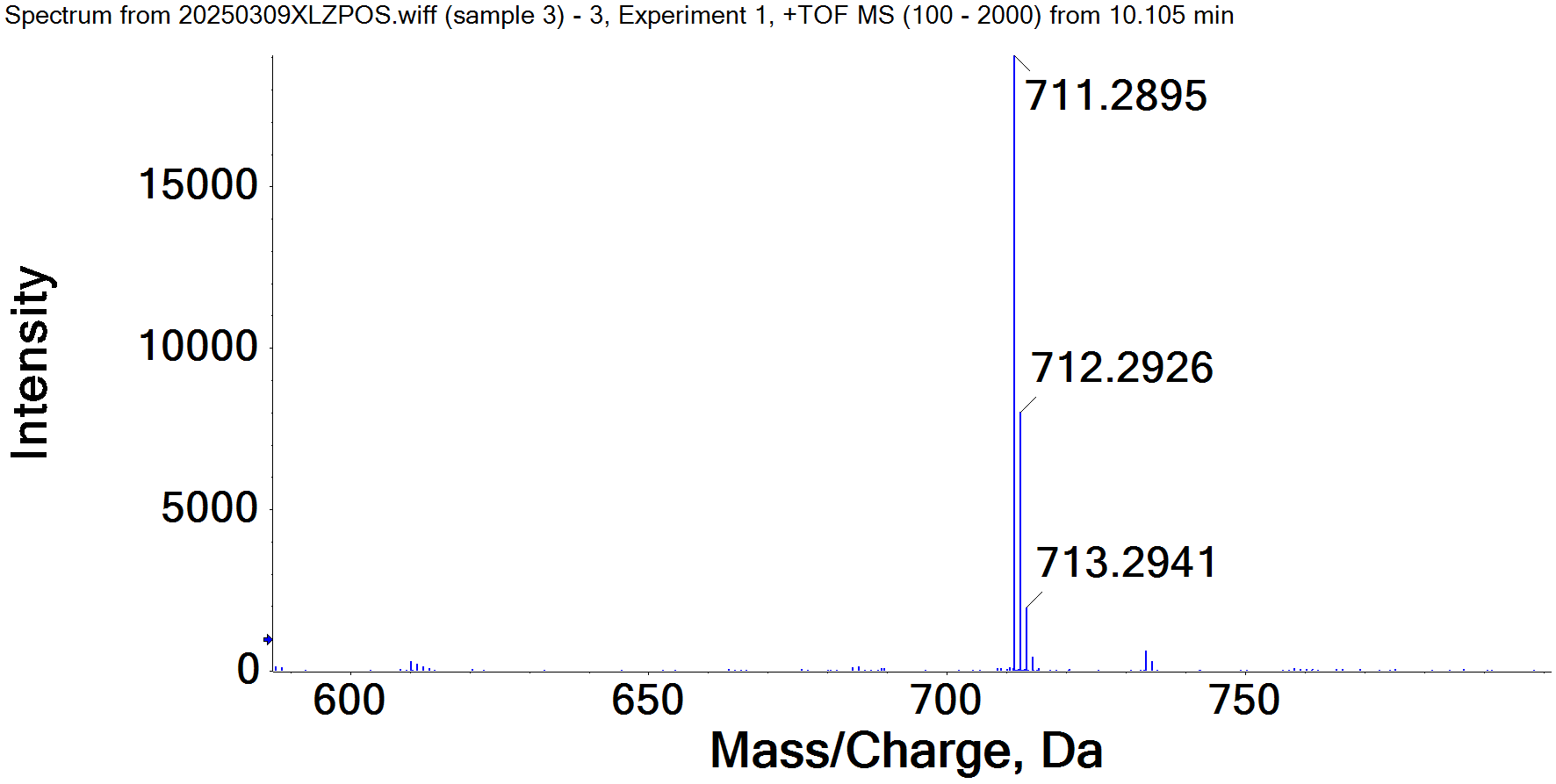


HRMS spectra of **8D**


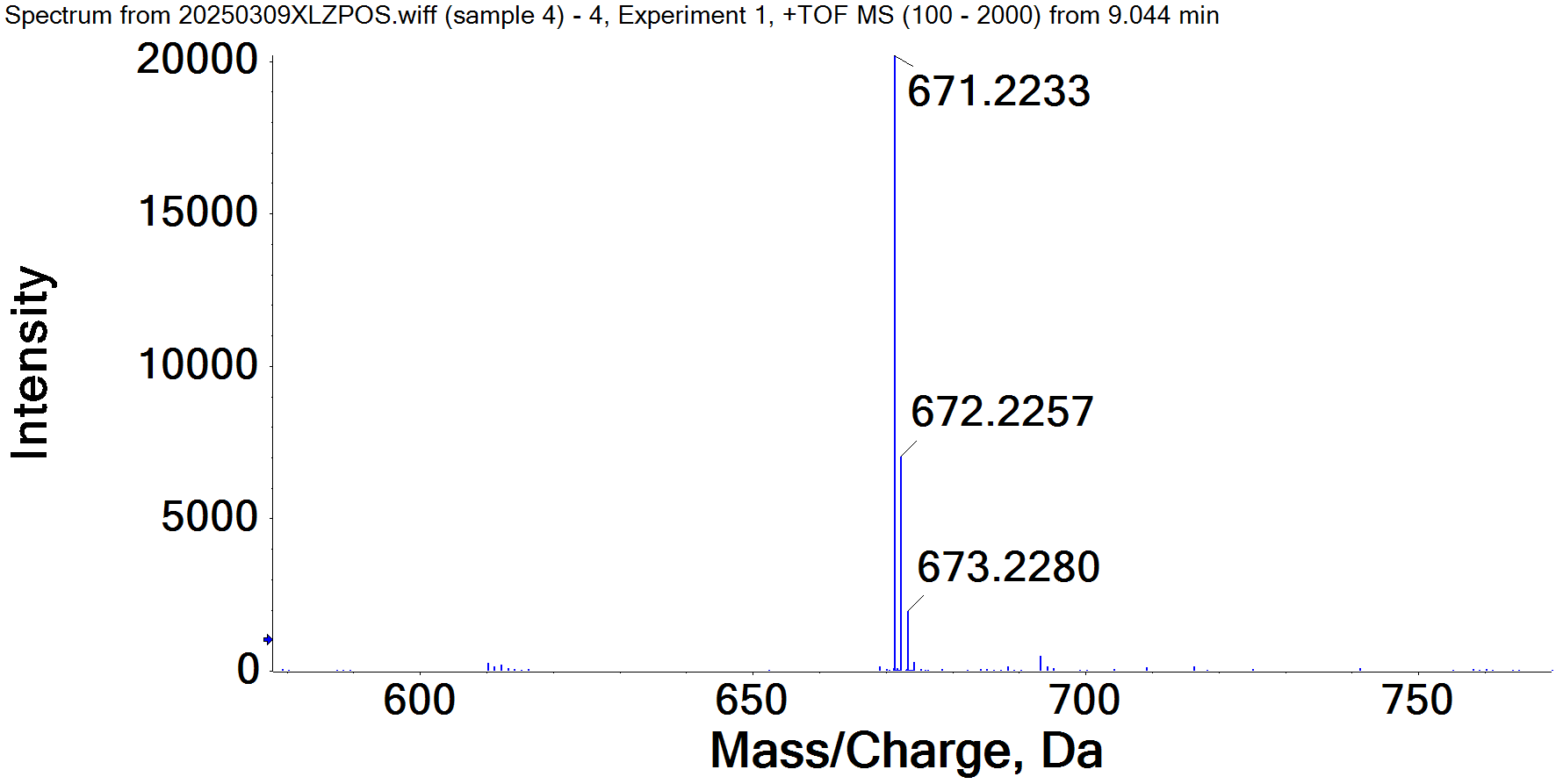


HRMS spectra of **8E**


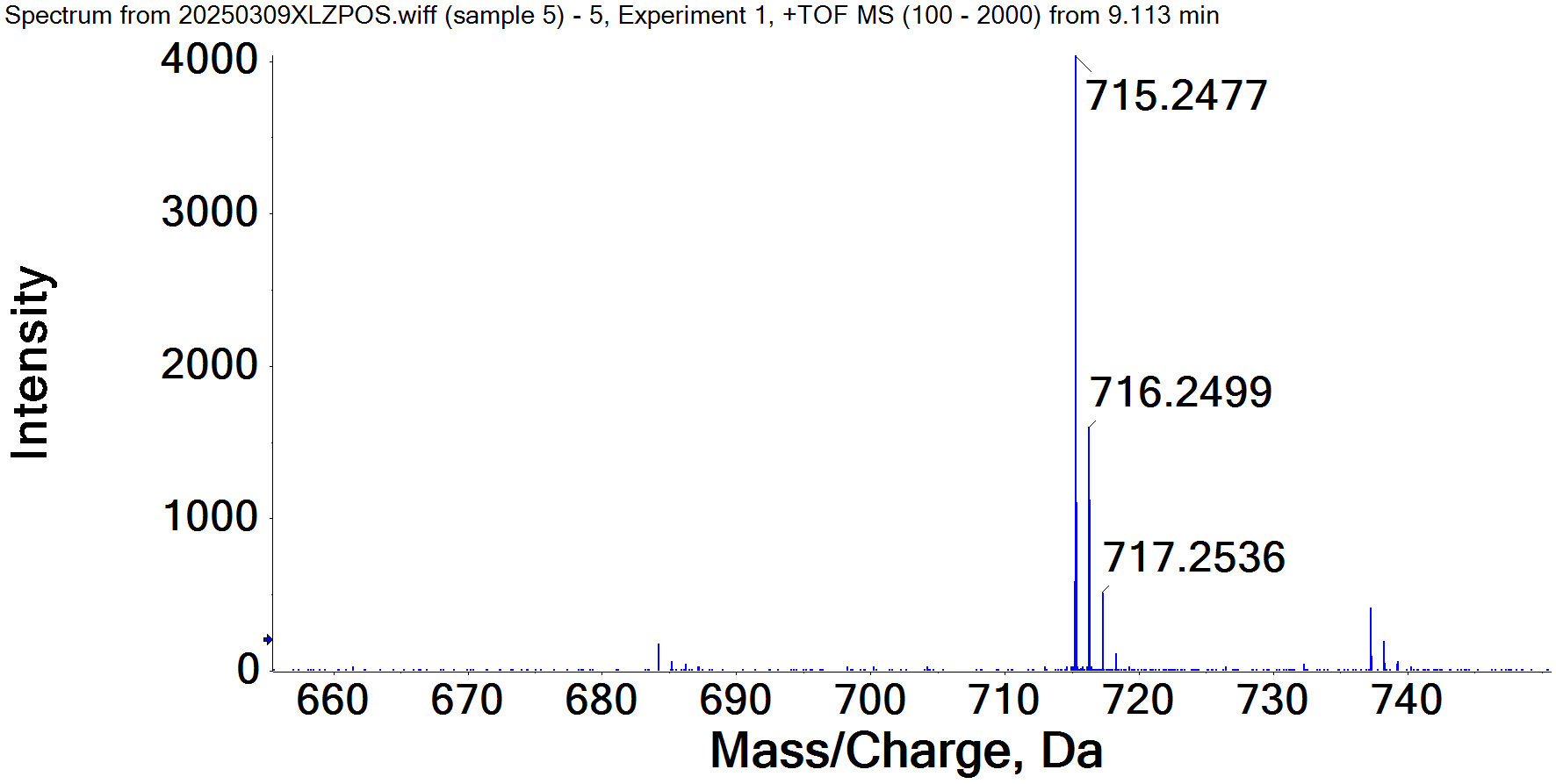


HRMS spectra of **8F**


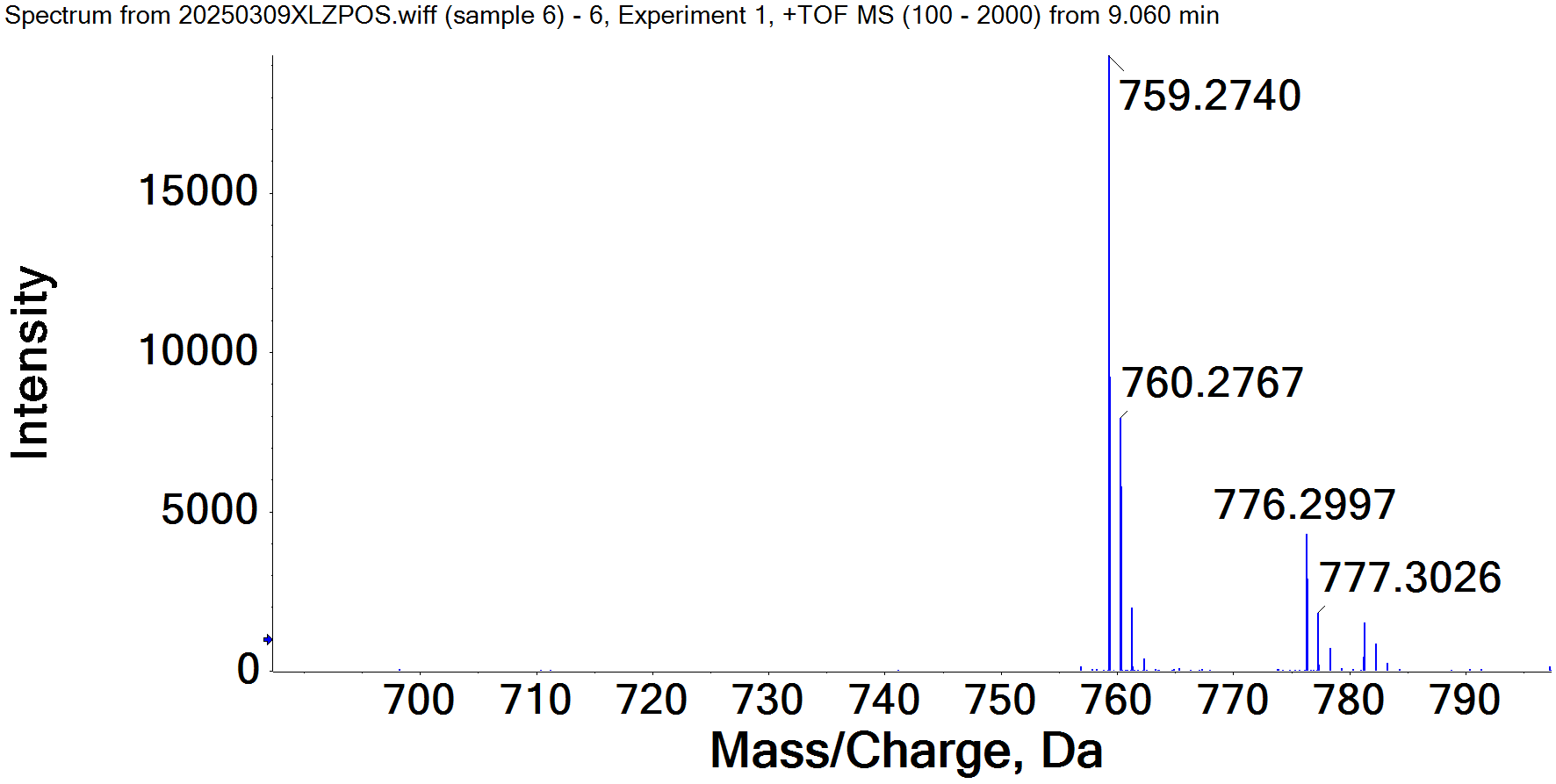


HRMS spectra of **8G**


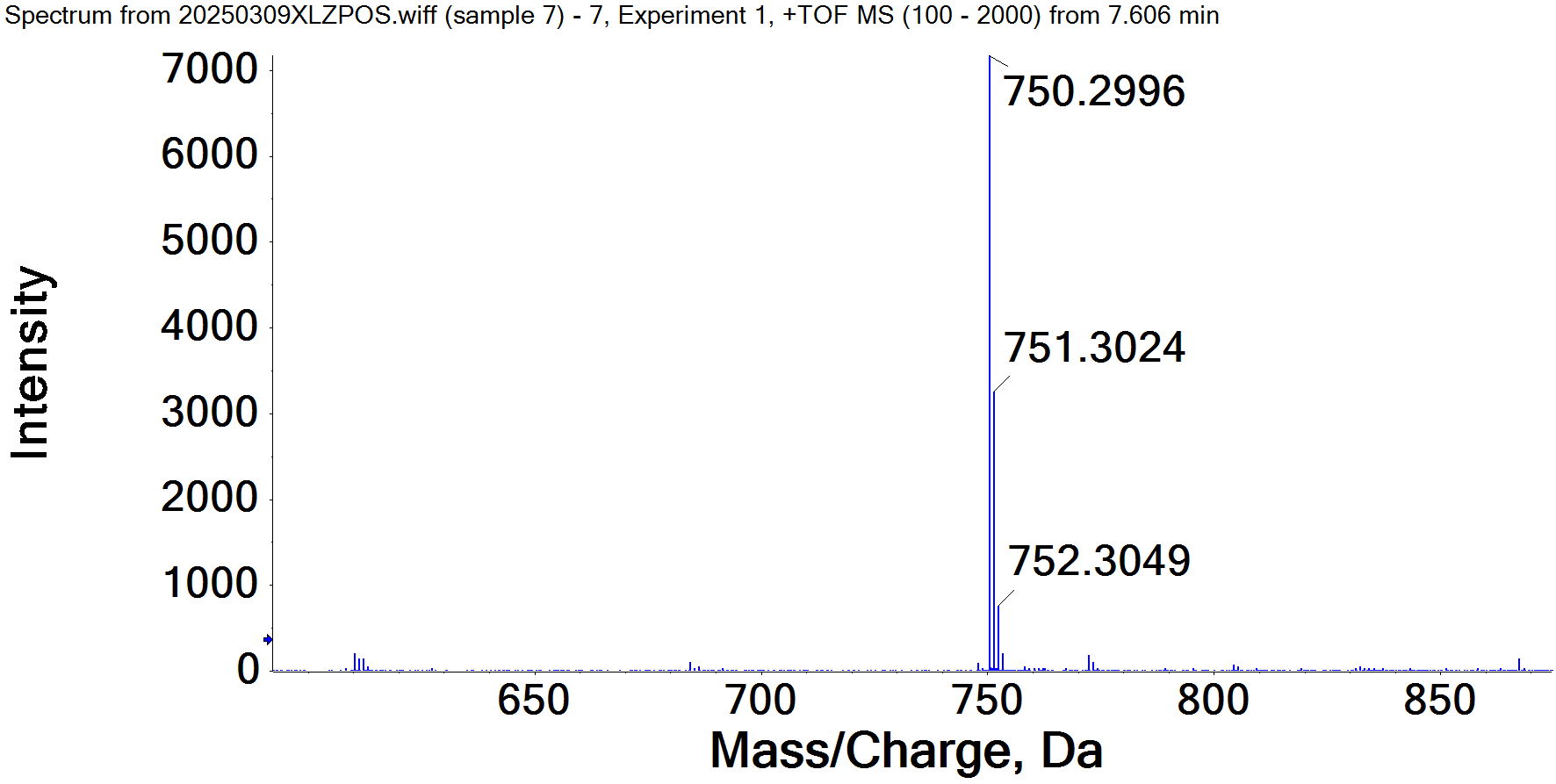


HRMS spectra of **8H**


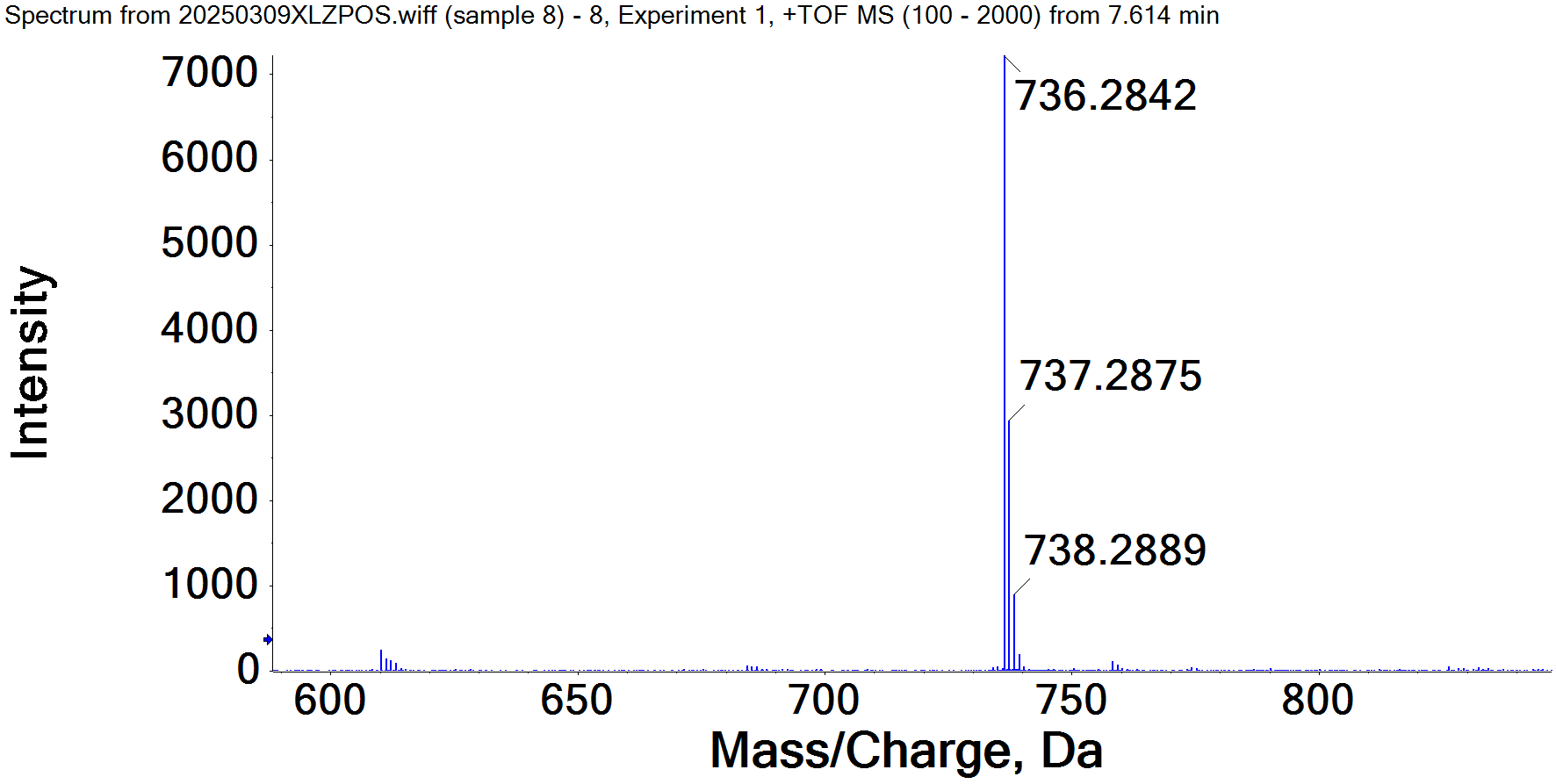


HRMS spectra of **8I**


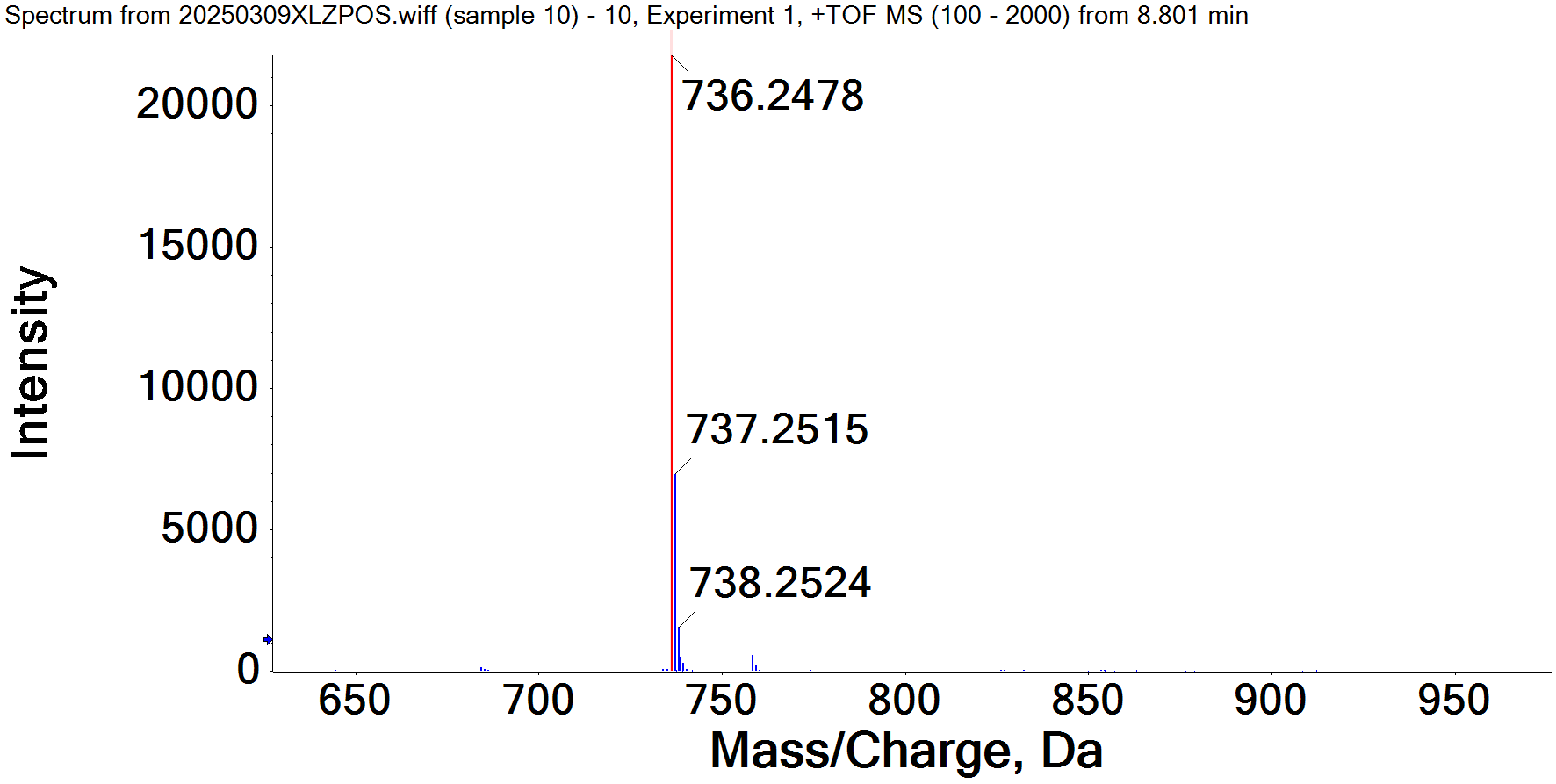


HRMS spectra of **8J**


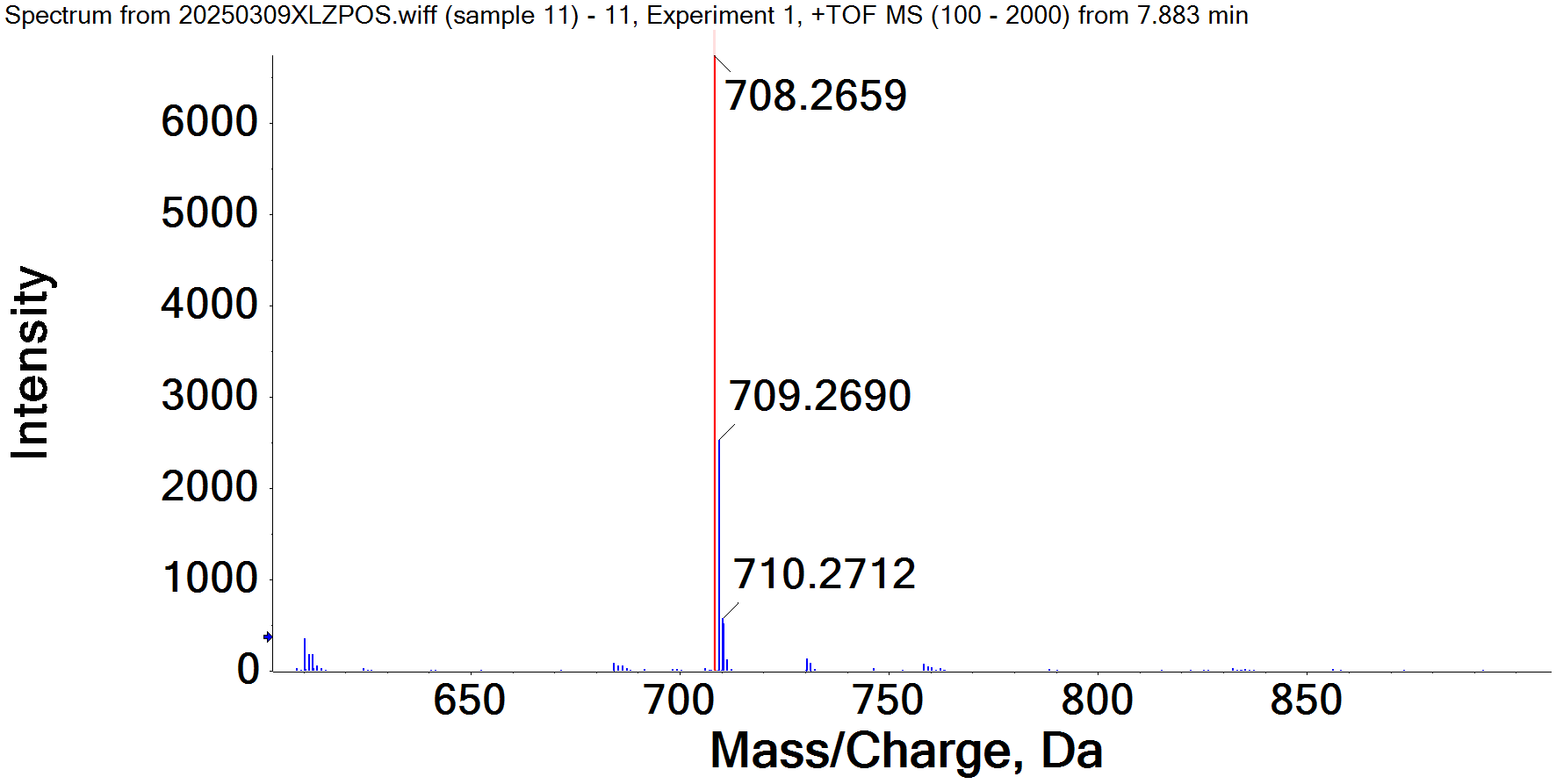


HRMS spectra of **8K**


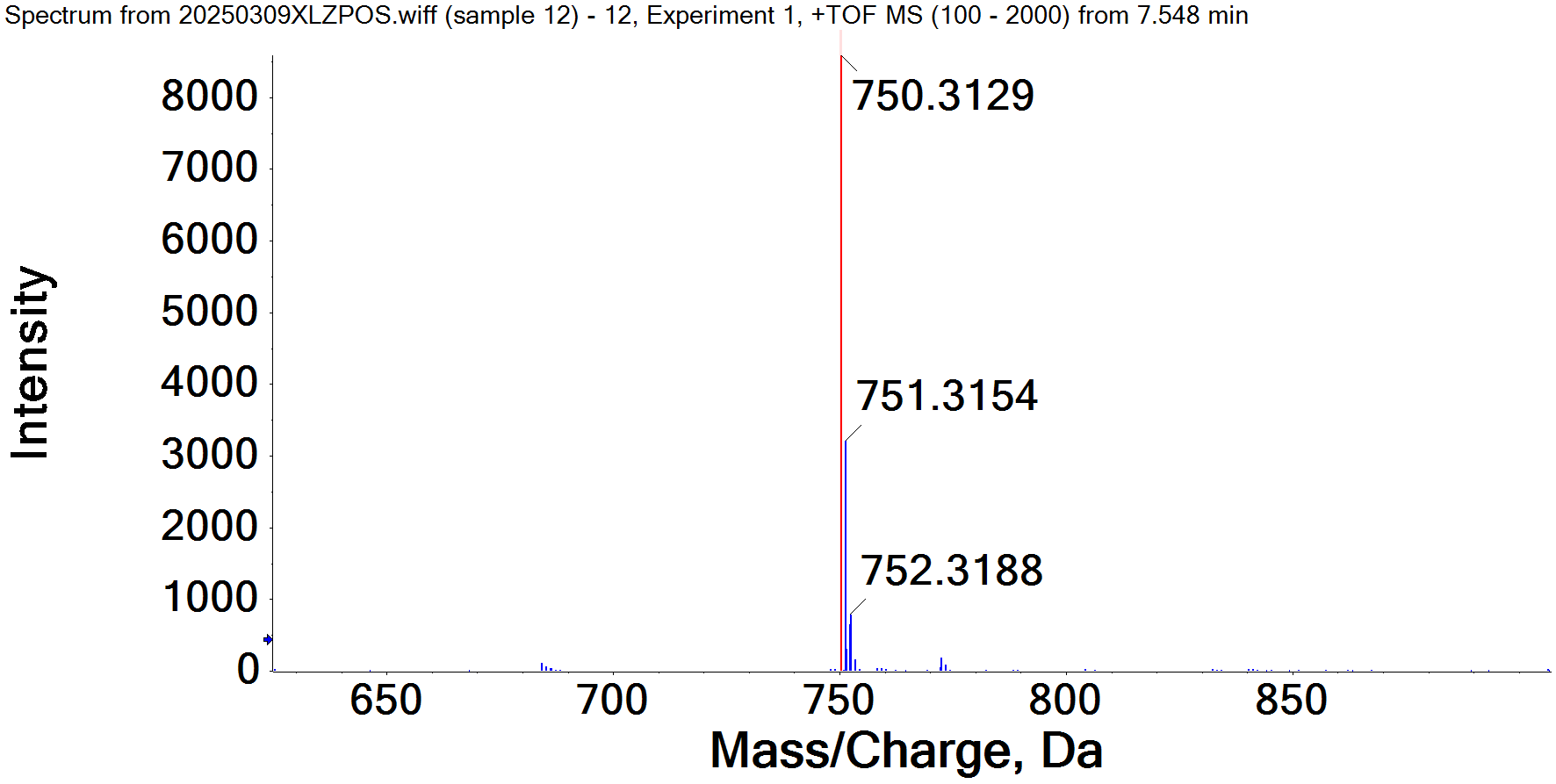


HRMS spectra of **8L**


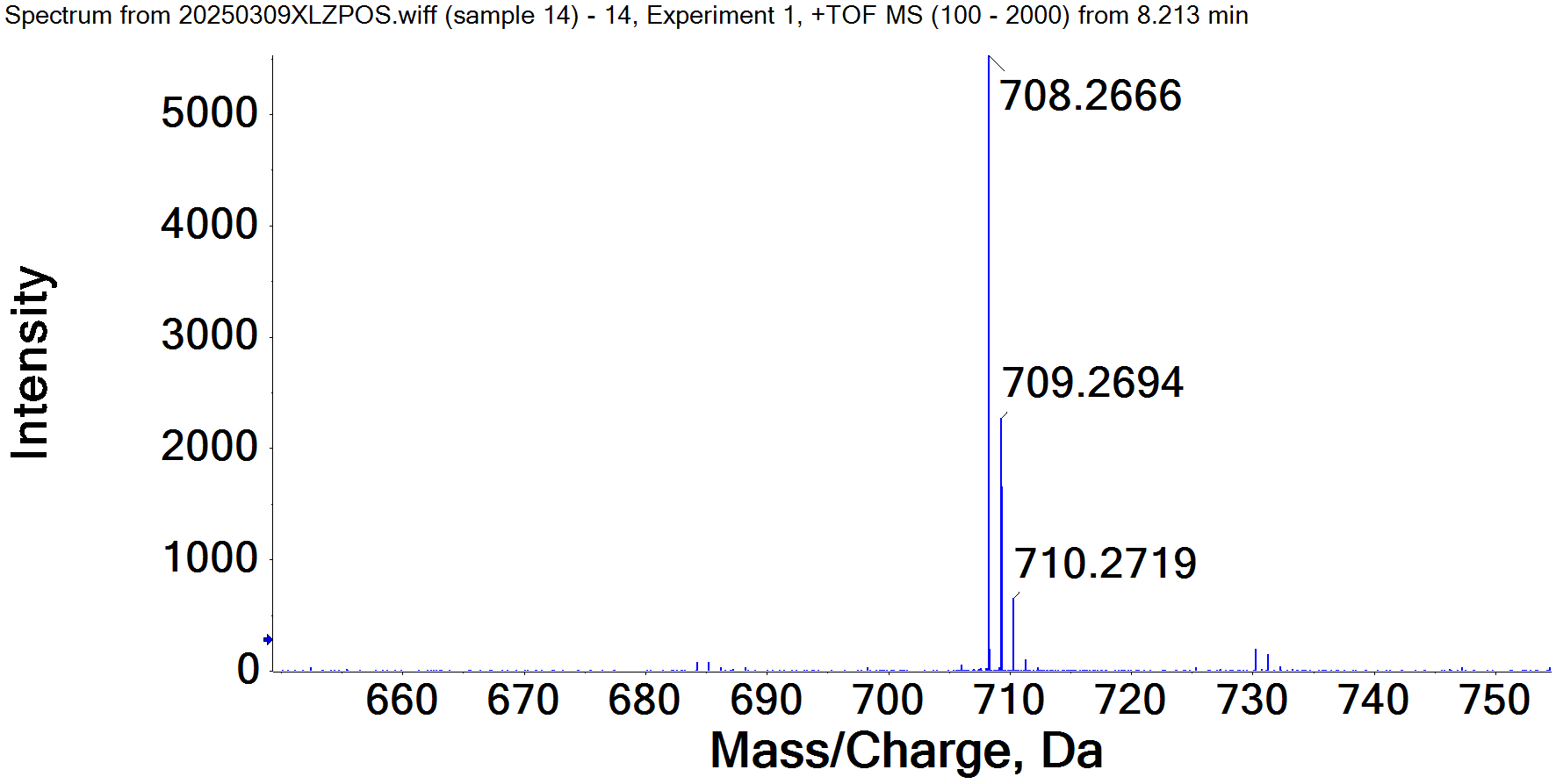


HRMS spectra of **8M**


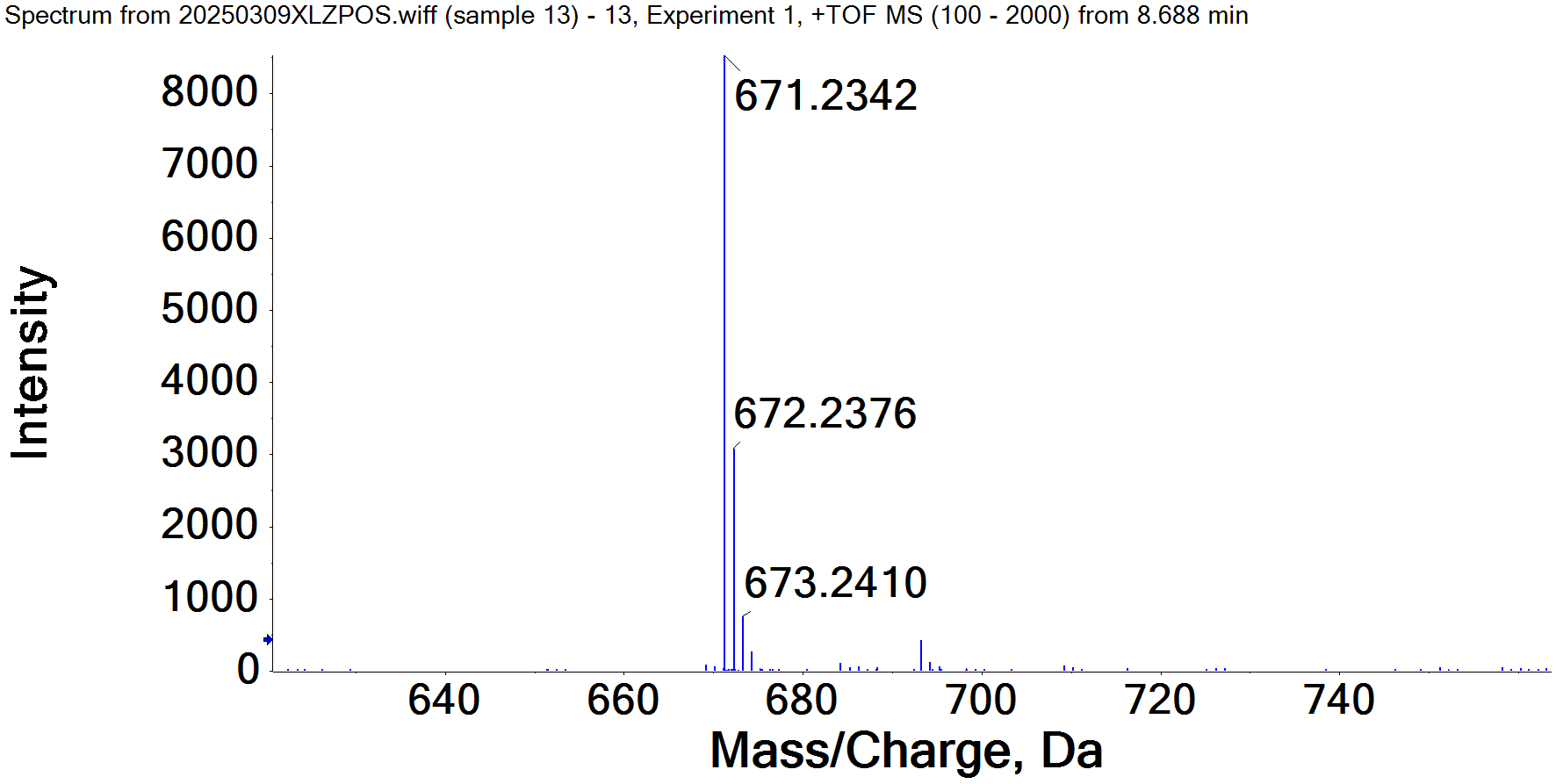

Supplement: Multimedia component 1 [file mmc1.docx]
